# An open-label single-arm phase 1/2a study to evaluate the safety and exploratory efficacy of a VM202 in patients with Charcot-Marie-Tooth disease 1A

**DOI:** 10.1186/s13023-026-04252-2

**Published:** 2026-03-05

**Authors:** Hye Mi Kwon, Hyun Su Kim, Sang Ah Chi, Soo Hyun Nam, Hye Jin Kim, Sang Beom Kim, Byung-Ok Choi

**Affiliations:** 1 Department of Precision Medicine, Sungkyunkwan University School of Medicine, Suwon, Republic of Korea; 2 Department of Radiology, Samsung Medical Center, Sungkyunkwan University School of Medicine, Seoul, Republic of Korea; 3 Biomedical Statistics Center, Research Institute for Future Medicine, Samsung Medical Center, Seoul, Republic of Korea; 4 Department of Health Sciences and Technology, Samsung Advanced Institute for Health Sciences & Technology, Sungkyunkwan University, Seoul, Republic of Korea; 5 Cell and Gene Therapy Institute, Samsung Medical Center, Seoul, Republic of Korea; 6 Department of Neurology, Kyung Hee University Hospital at Gangdong, Kyung Hee University School of Medicine, Seoul, Republic of Korea; 7 Department of Neurology, Samsung Medical Center, Sungkyunkwan University School of Medicine, 81 Irwon-ro, Gangnam-gu, Seoul, 06351 Republic of Korea

**Keywords:** Charcot-marie-tooth disease, Gene therapy, PMP22, Hepatocyte growth factor

## Abstract

**Background:**

This is the gene therapy trial in patients with Charcot-Marie-Tooth disease type 1A (CMT1A). Intramuscular injections of VM202, a plasmid DNA encoding human hepatocyte growth factor, was investigated for safe and exploratory effective in patients with CMT1A.

**Methods:**

This study was an open-label single-arm phase 1/2a trial. Thirteen patients with CMT1A were screened, and 12 consented and enrolled between September 2020 and November 2020. Patients received intramuscular injections of 14 mg of VM202 at baseline, and on days 14, 90, and 104 in both legs. Safety evaluations and clinical assessments using the CMT neuropathy score version 2 (CMTNSv2), CMT examination score (CMTES), Rasch-modified CMTNSv2 (CMTNSv2-R), Rasch-modified CMTES (CMTES-R), functional disability scale (FDS), overall neuropathy limitation score, and 10-meter walk test were performed throughout a 270-day follow-up period. A Wilcoxon signed-rank test was used for statistical comparisons of continuous variables.

**Results:**

The primary objective of this study was to assess the safety and tolerability of intramuscular injections of VM202 in patients with CMT1A. All participants tolerated VM202 well, without any serious adverse events related to the study drug. The secondary objective was to evaluate exploratory efficacy. CMTNSv2, CMTES, CMTNSv2-R, and CMTES-R decreased between baseline and day 270 with mean decreases of 2.17, 2.50, 2.08, and 2.33 points, respectively (*p* < 0.01). Also, FDS decreased with a mean percent decrease of 0.58 (*p* < 0.05). However, ONLS leg scale and 10-meter walk test were not statistically significant changes.

**Conclusions:**

Intramuscular injections of a VM202 appear to be safe and tolerated in CMT1A patients with an exploratory efficacy warrant confirmation in a phase 2b randomized controlled trial.

**Trial Registration Information:**

Name of the trial registry (VM202 for CMT1A), Registration number (NCT05361031), URL of the registry (http://www.clinicaltrials.gov) and date of registration (2022–03-31, retrospectively registered).

**Supplementary information:**

The online version contains supplementary material available at 10.1186/s13023-026-04252-2.

## Introduction

Charcot-Marie-Tooth disease (CMT) is one of the most common hereditary neuromuscular disorders, comprised of a group of clinically and genetically heterogeneous motor and sensory peripheral neuropathies [1]. Typical clinical manifestations include progressive distal muscle weakness, sensory loss, and areflexia, and more than 140 causative genes have been reported to date [1]. The most common form of the disease is CMT type 1A (CMT1A), which accounts for about half of all occurrences [2]. CMT1A is an autosomal dominant demyelinating polyneuropathy that results from overexpression of the peripheral myelin protein 22 (PMP22) gene on chromosome 17 [2]. PMP22 is one of the major constituents of myelin, which is present in the plasma membrane of Schwann cells [2]. Demyelination accompanied by hyperplasia of myelinating Schwann cells is the hallmark of CMT1A [3]. Although a substantial variability in the clinical course of the disease exists among patients, progressive neuromuscular deficit leading to significant clinical morbidity and impaired quality of life is common among the population [1, 4, 5]. Despite the advances in research made in understanding the genetic background of the disease, and promising results obtained from several approaches aiming at various pharmacological targets, there are no FDA-approved drugs for the treatment of CMT1A, and supportive measures remain the mainstay of the treatment [6, 7].

Hepatocyte growth factor (HGF) is a mesenchymal cell-derived regulator that was first discovered as a potent mitogen for hepatocytes and later found to contain various properties including neurotrophic, angiogenic, and antifibrotic activities [8]. These actions are mediated by the activation of its receptor, c-Met, which is expressed by various types of cells including Schwann cells, peripheral neurons, and muscle stem cells [9–11]. HGF has been reported to demonstrate trophic effects on peripheral sensory and motor neurons by enhancing axonal outgrowth and neuronal survival [11–13]. It acts through various mechanisms, including suppression of astrocytosis and microgliosis, reduction of cytotoxic cytokine release, facilitation of motor unit reinnervation, and limitation of cell death by inhibiting caspase signaling [14–16]. HGF has also been shown to improve neurogenic muscle atrophy by upregulating the expression of miR-206, a microRNA that is known to facilitate muscle differentiation by regulating the expression of myogenic regulatory factors [17].

VM202 (Engensis) is a novel nonviral plasmid DNA product that is designed to express two wild type isoforms of HGF: HGF_728_ and HGF_723_ [18]. Clinical trials of VM202 have demonstrated clinical efficacy for neurologic symptoms in patients with painful diabetic peripheral neuropathy and those with amyotrophic lateral sclerosis [11, 19]. Furthermore, the agent has proven to be safe and well tolerated in patients with a number of clinical conditions including ischemic heart disease and critical limb ischemia [11, 19–21]. The properties of HGF in regards to its interaction with peripheral nerve and muscle tissue, and previous clinical trial results make VM202 a promising for the treatment of CMT1A patients.

The primary objective of this study was to assess the safety and tolerability of intramuscular VM202 injections in patients with CMT1A. The secondary objective was to evaluate the potential clinical efficacy of the agent.

## Materials and methods

### Study design

This was an open-label single-arm phase 1/2a trial designed to evaluate the safety, tolerability, and exploratory efficacy of intramuscular injections of a VM202 in CMT1A patients. The primary endpoint was safety and tolerability. No single prespecified primary efficacy endpoint was defined because of the exploratory nature of the trial. CMTNSv2, CMTES, CMTNSv2-R, CMTES-R, FDS, ONLS, the 10-meter walk test, and electrophysiological parameters were analyzed as secondary or exploratory endpoints. No adjustment for multiplicity was performed, and all efficacy analyses should be interpreted as exploratory. The planned sample size of 12 was determined in consultation with a statistician based on feasibility considerations, including the rarity of CMT1A, the limited availability of genetically confirmed eligible patients from a single center, and the exploratory nature of this early-phase trial.

**Fig. 1:**
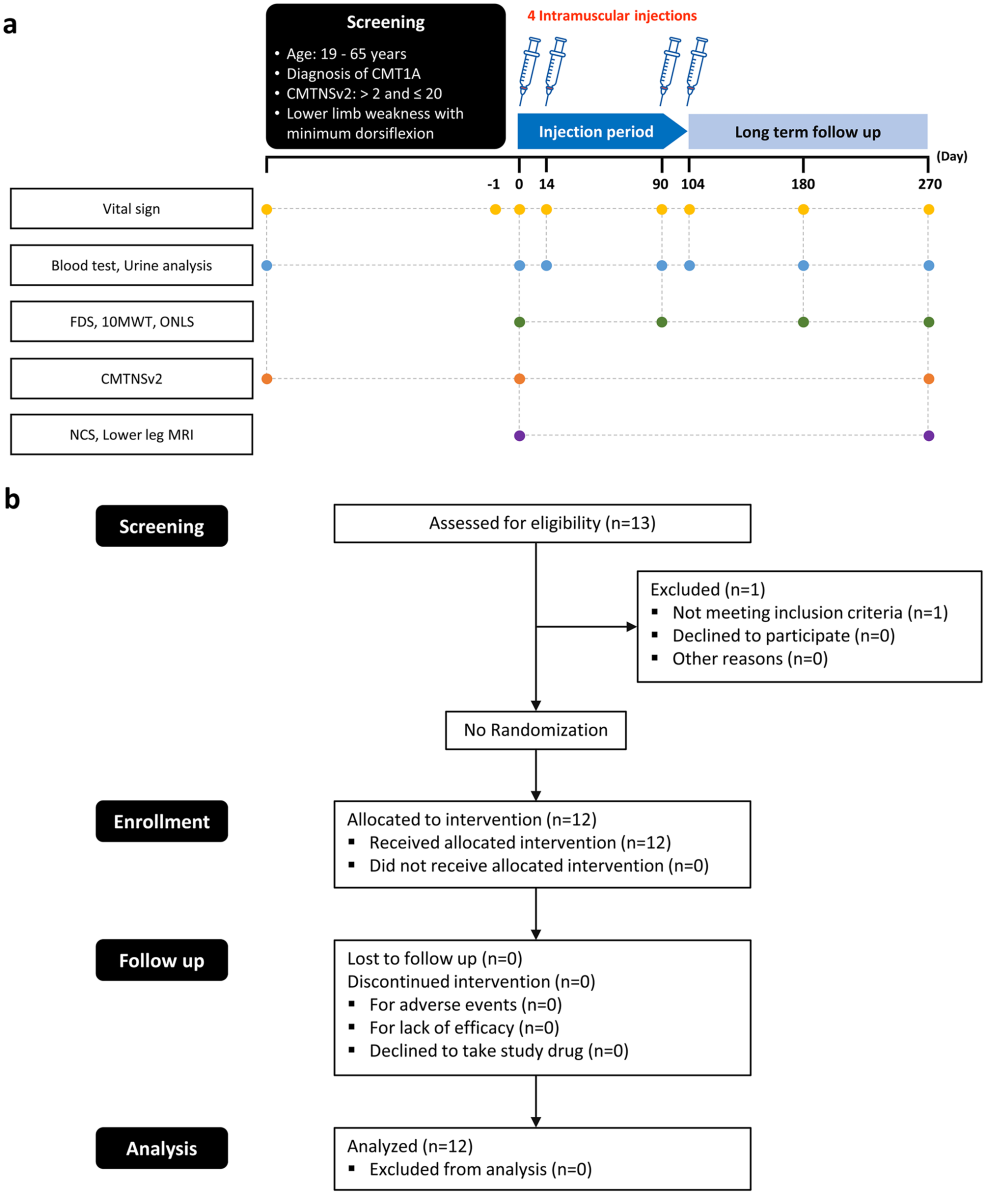
A trial design. b CONSORT diagram: participant enrollment, intervention allocation, and follow-up

Between September 2020 and November 2020, 13 patients with CMT1A were screened and 12 were consented and enrolled in the study. Patients received intramuscular injections of 14 mg of VM202 at baseline and on days 14, 90, and 104 in bilateral lower legs (Fig. 1a).

### Study drug

VM202 (pCK-HGF-X7) is a plasmid DNA designed to produce each of two isoforms of the HGF protein, consisting of either 723 or 728 amino acids; detailed information about VM202 has been described in previous studies [18, 21]. VM202 was supplied in a sterile vial and stored between 2 and 8 °C. It was reconstituted with 5.0 ml of sterile water for injections with a final VM202 concentration of 0.5 mg/ml. 14 mg of VM202 was administered via intramuscular injection using one-mL syringes with 29-gauge needles. Injections were given at the peroneus longus, gastrocnemius, and tibialis anterior muscles of bilateral lower legs at 6, 12, and 10 sites, with administered VM202 doses of 3, 6, and 5 mg, respectively.

### Patient eligibility

Eligible patients were non-pregnant individuals of ≥ 19 years to ≤ 65 years of age diagnosed with CMT1A through genetic analysis, had mild-to-moderate clinical severity with a CMT neuropathy score version 2 (CMTNSv2) between 3 and 20, and presented ankle dorsiflexion weakness. Female patients were advised to avoid becoming pregnant during the follow-up period (Fig. 1b).

Patients were excluded if they had comorbidities such as renal failure, chronic liver disease, inflammatory bowel disease, diabetes, other neuromuscular disorders, major psychiatric disorders, any conditions that could confound the study assessment; morbid obesity with a body mass index of ≥37; or uncontrolled hypertension with systolic blood pressure of ≥ 160 or diastolic blood pressure of ≥100. Also excluded were patients who had experienced a major cerebrovascular or cardiovascular event within the 12 months prior, ankle contracture, lower extremity surgery that could influence the evaluation of lower extremity muscle function; or an orthopedic procedure of the lower extremity within the 6 months prior. Patients were also considered exclusionary if they were receiving immunosuppressive medications, chemotherapy or radiation therapy; had evidence of malignancies other than fully resolved basal or squamous cell carcinoma of the skin; or had positive human immunodeficiency virus, hepatitis B, or hepatitis C serological test results.

This study was approved by the institutional review board at our institution (IRB No.SMC 2020–05-038–003). All patients gave written informed consent to participate in the study.

### Safety parameters

Safety was the primary end point and was assessed by evaluating all adverse events, conducting complete blood cell counts, serum chemistries, and urinalysis with microscopy at baseline, and on days 14, 90, 104, 180, and 270. Vital signs including systolic and diastolic blood pressure, body temperature, pulse rate, and respiration rate were also assessed likewise, and pre- and post-injection values were assessed on injection visits. Serum levels of anti-HGF antibodies were assessed at the baseline and at day 270 using an enzyme-linked immunosorbent assay (ELISA) developed by Helixmith Co., Ltd (Seoul, Korea).

### Clinical assessment

Clinical outcome measures for CMT1A including the CMTNSv2, CMT Examination Score (CMTES), Rasch-modified CMTNSv2 (CMTNSv2-R), and Rasch-modified CMTES (CMTES-R) were evaluated at baseline and at day 270 [22–24]. CMTNSv2 is a composite score consisting of clinical symptoms, signs, and neurophysiologic components. The CMTES represents the CMTNSv2 without the electrophysiological items. All items of CMTNSv2 and CMTES contribute identically to the total score. Otherwise, the CMTNSv2-R and CMTES-R consist of weighted category responses for representing more accurate estimates of the actual values measuring disease severity. The CMTNSv2 ranges from 0 (no deficit) to 36 (maximal deficit) [24]. This scale assigns mild impairment as scores 0–10, moderate as 11–20, and severe as 21–36. The maximum CMTES is 28 [23]. The CMTES assigns mild impairment as scores 0–7, moderate as 8–14, and severe as 15–28. In addition, disease severity based on the CMTNSv2-R was classified into three groups (mild 0–10, moderate 11–20, and severe 21–40), as it was for the CMTES-R (mild 0–9, moderate 10–18, and severe 19–32) [23]. CMTNSv2, CMTES, CMTNSv2-R and CMTES-R assessments were performed by two trained neurologists who were blinded to the assessment time points. Concomitant interventions including physiotherapy, orthotic use, and exercise programs were required to remain stable throughout the study period.

### Functional assessment

Lower extremity function of the patients was assessed using a functional disability scale (FDS), the ONLS (Overall Neuropathy Limitation Score) leg scale, and a 10-meter walk test (10MWT) at baseline and at day 270. A nine-point FDS was assessed to determine disease severity in terms of the ability to walk and run as follows: 0 = normal; 1 = normal, but with cramps and fatigability; 2 = inability to run; 3 = walking difficult but still possible unaided; 4 = able to walk with a cane; 5 = able to walk with crutches; 6 = able to walk with a walker; 7 = wheelchair bound; 8 = bedridden [22]. A seven-point ONLS leg scale was assessed to measure the limitations of the lower limbs as follows: 0 = walking/climbing stairs/running not affected; 1 = walking/climbing stairs/running affected, but gait does not look abnormal; 2 = walks independently but gait looks abnormal; 3 = requires unilateral support to walk 10 meters (stick, single crutch, one arm); 4 = requires bilateral support to walk 10 meters (sticks, crutches, crutch and arm, frame); 5 = requires wheelchair to travel 10 meters but able to stand and walk 1 meter with the help of one person; 6 = restricted to wheelchair, unable to stand and walk 1 meter with the help of one person, but able to make some purposeful leg movements; 7 = restricted to wheelchair or bed most of the day, unable to make any purposeful movements of the legs [25]. For the 10MWT, patients were asked to walk a 10-meter distance, and the time taken was measured in seconds. Three sets of measurements were taken to obtain an average value, which was measured at baseline and at day 270.

### Electrophysiologic studies

Electrophysiologic studies were performed to assess the nerve regeneration potential of the patients. Motor nerve conduction velocities (MNCVs), compound muscle action potentials (CMAPs), sensory nerve conduction velocities (SNCVs), and sensory nerve action potentials (SNAPs) were measured at baseline and at day 270. MNCVs and CMAPs were measured for bilateral median, ulnar, radial, tibial, and peroneal nerves. SNCVs and SNAPs were measured for bilateral median, ulnar, and sural nerves.

### Statistical analysis

Safety and tolerability were assessed for the safety set, defined as all participants who revised at least one dose of a VM202. Efficacy analyses were based on the intention-to-treat (ITT) population including all participants who completed baseline and at least one post-baseline efficacy assessment. All 12 enrolled participants were included in both the safety and exploratory efficacy analyses. A Wilcoxon signed-rank test for continuous variables was performed to assess the clinical outcomes between baseline and day 270 after intramuscular injection of VM202. Statistical significance was defined at a two-sided *p*-value of < 0.05. All statistical analyses were performed using R Statistical Software (version 3.6.3; Foundation for Statistical Computing, Vienna, Austria

## Results

### Safety and tolerability

Detailed demographics of the participants are shown in Table 1, and detailed incidents of adverse events are summarized in Table 2. A total of four adverse events were reported in three patients (25%). There were no serious adverse events attributable to VM202. Injection site pruritus and peripheral edema, which occurred in separate patients (2/12, 16.7%) and were mild in degree, were the only adverse events that were possibly related to the treatment. They resolved without additional treatment. Two serious adverse events were observed in one patient (1/12, 8.3%; they were pneumonia and uterine myoma. They were non-drug-related and no death was observed during the study. No clinically significant changes compared with those assessed at the baseline were demonstrated in serum, urine lab results, or vital signs. No patients developed antibodies to the HGF protein during the study period.

**Table 1:** Baseline demographics of the 12 participants

	Total (N = 12)
**Sex**	
Male	7 (58.3%)
Female	5 (41.7%)
**Age (years)**	
Mean±SD	40.2 ± 14.8
Median (range)	42 (19–60)
**Height (cm)**	
Mean±SD	168.8 ± 9.3
Median (range)	171.8 (153.8–180.1)
**Weight (kg)**	
Mean±SD	68.3 ± 15.5
Median (range)	68.7 (36.4–93.3)
**BMI (kg/m**^**2**^)	
Mean±SD	23.7 ± 3.7
Median (range)	23.2 (15.4–28.8)
**Smoking**	
Current smoker	1 (8.3%)
Former smoker	2 (16.7%)
Non-smoker	9 (75.0%)
**Alcohol**	
Current drinker	5 (41.7%)
Former drinker	1 (8.3%)
Non-drinker	6 (50.0%)

*BMI* body mass index, *SD* standard deviation

**Table 2:** Adverse events reported from the study

Screening No.	Adverse event	Severity	Relationship with gene therapy	Outcome
S01013	Injection site pruritus NOS	Mild	Possibly related	Resolved
S01006	Ankle edema	Mild	Possibly related	Resolved
S01008	Pneumonia	Severe	Not related	Resolved
S01008	Uterine myoma	Severe	Not related	Resolved

### Clinical severity evaluation

Statistical comparison results of the CMTNSv2, CMTES, CMTNSv2-R and CMTES-R assessed at the baseline and at day 270 are summarized on Table 3 and Additional file 1: Fig. S1a. The total CMTNSv2, CMTES, CMTNSv2-R, and CMTES-R decreased between the two timepoints, with mean decreases of 2.17 (*p <* 0.01), 2.50 (*p <* 0.01), 2.08 (*p <* 0.01), and 2.33 points (*p <* 0.01), respectively. Sign and symptom components of CMTNSv2 also showed numerical changes in exploratory analyses between the two timepoints (*p* = 0.0035 and 0.0069, respectively). These improvements did arise from sensory symptoms and sensory signs rather than motor symptoms and strength (Additional file 1: Table S1). Table 4 summarizes the score groups of CMTNSv2, CMTNSv2-R, CMTES, and CMTES-R between two time points. The patients in the severe or moderate groups of CMTNSv2-R, CMTES, and CMTES-R improved to mild or moderate groups by day 270.

**Table 3:** Changes in CMTNSv2, CMTES, CMTNSv2-R, CMTES-R, and CMTNSv2 components from baseline to 270 days. Comparison results of the functional disability scale, overall neuropathy limitation score leg scale, and 10-meter walk test time

	Baseline (N = 12)		Day 270 (N = 12)		Change between the two timepoints		p
	Mean±SD	Median	Mean±SD	Median	Mean±SD	Median	
**Functional assessments**							
CMTNSv2	15.6 ± 3.4	16.5	13.4 ± 2.7	14.5	−2.17±1.11	−2.0	0.0035^*^
CMTES	12.1 ± 2.8	12.5	9.6 ± 2.3	10.5	−2.50±1.31	−3.0	0.0036^*^
CMTNSv2-R	18.2 ± 3.4	18.5	16.1 ± 3.1	16.5	−2.08±1.31	−2.0	0.0036^*^
CMTES-R	14.4 ± 2.9	15.3	12.1 ± 2.4	12.1	−2.33±1.50	−2.5	0.0035^*^
FDS	2.67 ± 0.65	2.67	2.08 ± 0.79	2.08	−0.58±0.51	−1.00	0.0107^*^
ONLS leg scale	2.08 ± 0.29	2.08	1.75 ± 0.45	1.75	−0.33±0.49	0	0.0719
10MWT time (sec)	8.96 ± 1.96	9.02	8.34 ± 1.27	8.3	−0.62±1.75	−0.44	0.3013
**CMTNSv2 components**							
Signs	7.3 ± 1.7	7.5	5.8 ± 1.4	6.0	−1.50±0.80	−2.0	0.0035^*^
Symptoms	4.8 ± 1.3	5.0	3.8 ± 1.1	4.0	−1.00±0.74	−1.0	0.0069^*^
Neurophysiologic component	3.5 ± 0.9	4.0	3.8 ± 0.7	4.0	0.33 ± 0.49	0.33	0.0719
**CMAP summatory (mV)**^**†**^	41.9 ± 14.7	41.0	43.3 ± 17.4	40.26	1.42 ± 14.78	1.42	0.1424
**MNCV (m/s)**^‡^	19.6 ± 3.9	18.75	19.6 ± 3.4	19.13	0.06 ± 1.48	−0.13	0.9185

*CMAP* compound muscle action potential, *CMTES* CMT examination score, *CMTES-R* Rasch-modified CMTES, *CMTNSv2* CMT neuropathy score version 2, *CMTNSv2-R* Rasch-modified CMT neuropathy score version 2, *FDS* functional disability scale, *ONLS* overall neuropathy limitation score, *10MWT* 10-meter walk test

Indicates statistical significance

CMAP summatory: sum of compound action potential of the three motor nerves (ulnar, median, and peroneal nerves)

Mean from median and ulnar nerves

**Table 4:** Group changes between the CMT neuropathy score version 2 and CMT examination score

	Baseline (%) (n = 12)	Day 270%) (n = 12)
**CMTNSv2**		
Mild (0–10)	2 (16.7%)	2 (16.7%)
Moderate (11–20)	10 (83.3%)	10 (83.3%)
Severe (21–36)	0 (0%)	0 (0%)
**CMTNSv2-R**		
Mild (0–10)	0 (0%)	1 (8.3%)
Moderate (11–20)	9 (75%)	11 (91.7%)
Severe (21–40)	3 (25%)	0 (0%)
**CMTES**		
Mild (0–7)	1 (8.3%)	2 (16.7%)
Moderate (8–14)	9 (75%)	10 (83.3%)
Severe (15–28)	2 (16.7%)	0 (0%)
**CMTES-R**		
Mild (0–9)	2 (16.7%)	2 (16.7%)
Moderate (10–18)	9 (75%)	10 (83.3%)
Severe (19–32)	1 (8.3%)	0 (0%)

*CMTNSv2* CMT neuropathy score version 2, *CMTNSv2-R* Rasch-modified CMT neuropathy score version 2, *CMTES* CMT examination score, *CMTES-R* Rasch-modified CMT examination score

### Function of lower extremities

Table 3 and Additional file 1: Fig. S1b summarize the statistical comparison results of the FDS, ONLS, and 10MWT times assessed at the baseline and at 270 days. The FDS decreased between the baseline and day 270, with a mean decrease of 0.58 (*p* < 0.05). The ONLS leg scale and 10MWT times gradually decreased, but the changes were not statistically significant.

## Discussion

This prospective trial was the first clinical study to assess the safety and tolerability of intramuscular VM202 injections in patients with CMT1A. We observed acceptable short-term tolerability of intramuscular administration of plasmid DNA expressing two isoforms of human HGF in CMT1A patients. Moreover, we observed numerical decreases in several clinical scores following VM202 administration in CMT1A patients.

There were no serious adverse events attributable to VM202. The injection site pruritus and peripheral edema that occurred in a small number of patients were the only adverse events that were possibly related to the agent, and both resolved without treatment. VM202 was well tolerated without any reports of serious adverse events associated with the agent in studies performed on painful diabetic peripheral neuropathy, amyotrophic lateral sclerosis, and critical limb ischemia patients [11, 19, 20]. The safety profile of VM202 can be explained by the fact that HGF expression from VM202 is short-lived and tends to be restricted to the area of injection [11, 19]. Heparan sulfate, which is abundant in the extracellular matrix, limits the diffusion of secreted HGF by binding to the N-terminal of HGF [11]. Even though plasmid DNA and the HGF protein can reach circulation, the possible biologic effect would be insignificant, as their half-lives in the bloodstream are only a few minutes long [26, 27]. Furthermore, no patients developed antibodies to the HGF protein in our study. Taken together with the safety results of previous studies that this clinical trial support the acceptable short-term tolerability and safety of intramuscular VM202 in a small cohort of CMT1A patients, however, larger or longer studies are needed to establish long-term safety.

The pathologic hallmark of CMT1A patients is demyelination associated with length-dependent axonal degeneration of both sensory and motor nerves [3, 28]. Disability in CMT1A patients has been reported to correlate with the degree of axonal degeneration [29]. Thus, it has been suggested that therapeutic approaches to ameliorate disability in CMT1A patients should be directed toward preventing axonal degeneration and promoting axonal regeneration [28]. However, current treatment options are mostly palliative, rather than targeting the mechanisms underlying CMT1A. Animal studies have revealed that the intramuscular injection of VM202 leads to HGF production and that its interaction with the c-Met receptor present in Schwann cells and sensory neurons via ERK and AP-1 signaling pathways, respectively, promotes axon outgrowth [30, 31].

Clinical severity assessed by the CMTNSv2 showed numerical improvement at the last follow-up with a mean decrease of 2.17 points (*p* < 0.01). This was attributable to improvements of signs (*p* = 0.0050) and symptoms (*p* = 0.0147). Clinical disability scores including CMTES, CMTNSv2-R, and CMTES-R also showed numerical improvements between timepoints with mean decreases of 2.50, 2.08, and 2.33 points, respectively (*p <* 0.01). Although statistically significant numerical decreases of approximately 2–2.5 points were observed in CMTNSv2 and CMTES, the magnitude of these changes was modest. A score group comparison was conducted to find the clinical effects of the VM202 injections. The patients in the severe group of CMTNSv2-R, CMTES, and CMTES-R showed numerical changes in comparison to the mild and moderate groups. This suggests that VM202 injections had clinical benefits in CMT1A patients. In addition, function as assessed by the FDS decreased between the baseline and day 270 (*p* < 0.05).

In addition, function as assessed by the FDS decreased between the baseline and day 270 (*p* < 0.05). In the present study, improvements were observed mainly in clinical symptom and sign components. In the electrophysiological parameters results, CMAP summatory showed a slightly increased finding, but MNCV did not change. This discrepancy may reflect the limitations inherent to an open-label, single-arm design, including potential placebo effects, and learning effects in repeated functional assessments.

Although the true effect of transient gene expression via VM202 injection in CMT1A patients is not completely understood, theoretical background regarding the trophic effects of HGF on peripheral sensory, motor nerves, and muscle cells may explain the positive results of our study [11–13, 17].

One concern raised before initiating the study was that the relatively large number of intramuscular injections (56 sites of injection per visit) would not be tolerable to some patients. It was originally thought that VM202 injections would provide short-term therapeutic benefits, thus requiring repeated treatment, because plasmid DNA would be short-lived in vivo and give only transient gene expression. As such, it was initially expected that the discomfort caused by the multiple injections would be an issue. However, the fact that there were no withdrawals from this study suggests that the injection scheme could be well tolerated.

## Conclusions

This is the first phase 1/2a gene therapy trial performed for patients with CMT1A. Our data intramuscular administration of VM202 in CMT1A patients. Exploratory clinical signals were observed by improved clinical scores over the study period. Considering the high unmet medical need in CMT1A patients, performance of a phase 2b randomized, double-blind clinical trial in these patients is warranted.

## Electronic supplementary material

Below is the link to the electronic supplementary material.

Supplementary Material 1. Additional file 1: Table S1: The CMT neuropathy score version 2 and CMT examination score at baseline and day 270 for all PATIENTS. Figure S1: The statistical comparison results of the CMTNSv2, CMTES, CMTNSv2-R, CMTES-R, FDS, ONLS leg scale, and 10MWT time.

Supplementary Material 2

## Data Availability

The datasets used and/or analysed during the current study are available from the corresponding author on reasonable request.

## Abbreviations

10MWT: 10-meter walk test
CMAPs: Compound muscle action potentials
CMT: Charcot-Marie-Tooth disease
CMTES: CMT examination score
CMTES-R: Rasch-modified CMT examination score
CMTNSv2: CMT neuropathy score version 2
CMTNSv2-R: Rasch-modified CMT neuropathy score version 2
FDS: Functional disability scale
HGF: Hepatocyte growth factor
MNCVs: Motor nerve conduction velocities
NCS: Nerve Conduction Study
ONLS: Overall Neuropathy Limitation Score
SNAPs: Sensory nerve action potentials
SNCVs: Sensory nerve conduction velocities
