# Supplementary material for: An open-label single-arm phase 1/2a study to evaluate the safety and exploratory efficacy of a VM202 in patients with Charcot-Marie-Tooth disease 1A
Source: Orphanet J Rare Dis. 2026 Mar 5;21:148. doi: 10.1186/s13023-026-04252-2 (PMC13072572; doi:10.1186/s13023-026-04252-2)
Supplement: Supplementary file 1 — Supplementary Material 1. Additional file 1: Table S1: The CMT neuropathy score version 2 and CMT examination score at baseline and day 270 for all PATIENTS. Figure S1: The statistical comparison results of the CMTNSv2, CMTES, CMTNSv2-R, CMTES-R, FDS, ONLS leg scale, and 10MWT time. [file 13023_2026_4252_MOESM1_ESM.docx]

**Fig. S1** The statistical comparison results of the CMTNSv2, CMTES, CMTNSv2-R, CMTES-R, FDS, ONLS leg scale, and 10MWT time. (**a**) Changes in FDS, ONLS leg scale, 10MWT time, and the total CMTNSv2 between baseline and at day 270. (**b**) Changes in the total CMTNSv2, CMTNSv2-R, CMTES, and CMTES-R between baseline and day 270. Error bars indicate standard deviation. Asterisk indicates a statistically significant difference from the value at the baseline.


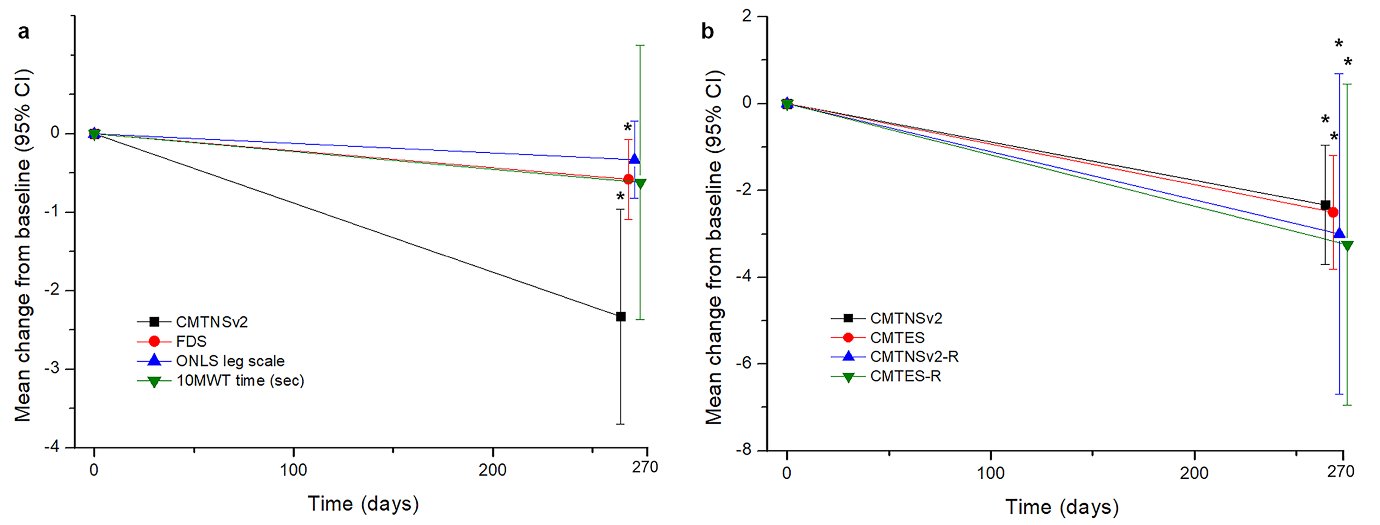


# **Table S1** The CMT neuropathy score version 2 and CMT examination score at baseline and day 270 for all patients

| **Screening number** | **Visit** | **Symptoms** | | |  | **Signs** | | | |  | **Neurophysiologic component** | |  | **Total Score^[10]^** |
| --- | --- | --- | --- | --- | --- | --- | --- | --- | --- | --- | --- | --- | --- | --- |
|  |  | **Sensory symptoms^[1]^** | **Motor symptoms (legs)^[2]^** | **Motor symptoms (arms)^[3]^** |  | **Pinprick sensibility^[4]^** | **Vibration^[5]^** | **Strength (legs)^[6]^** | **Strength (arms)^[7]^** |  | **Ulnar CMAP ^[8]^** | **Radial SNAP (antidromic)^[9]^** |  |  |
| S01001 | Baseline | 1 | 1 | 0 |  | 1 | 1 | 1 | 1 |  | 0 | 3 |  | Mild |
|  | Day 270 | 1 | 1 | 0 |  | 1 | 1 | 1 | 1 |  | 0 | 3 |  | Mild |
| S01003 | Baseline | 4 | 1 | 1 |  | 2 | 4 | 1 | 1 |  | 0 | 4 |  | Moderate |
|  | Day 270 | **3** | 1 | 1 |  | **1** | **3** | 1 | 1 |  | 0 | 4 |  | Moderate |
| S01004 | Baseline | 2 | 2 | 1 |  | 2 | 2 | 1 | 1 |  | 0 | 4 |  | Moderate |
|  | Day 270 | **1** | 2 | 1 |  | **1** | 2 | 1 | 1 |  | 0 | 4 |  | Moderate |
| S01005 | Baseline | 2 | 1 | 0 |  | 1 | 2 | 1 | 1 |  | 0 | 2 |  | Mild |
|  | Day 270 | 2 | **0** | 0 |  | **0** | **1** | 1 | 1 |  | 0 | 3 |  | Mild |
| S01006 | Baseline | 3 | 1 | 1 |  | 2 | 3 | 1 | 1 |  | 0 | 3 |  | Moderate |
|  | Day 270 | **1** | 1 | 1 |  | **1** | **2** | 1 | 1 |  | 0 | 4 |  | Moderate |
| S01007 | Baseline | 3 | 2 | 1 |  | 2 | 3 | 1 | 1 |  | 0 | 4 |  | Moderate |
|  | Day 270 | **2** | 2 | 1 |  | **1** | **2** | 1 | 1 |  | 1 | 4 |  | Moderate |
| S01008 | Baseline | 3 | 1 | 1 |  | 3 | 4 | 2 | 1 |  | 1 | 4 |  | Moderate |
|  | Day 270 | **2** | 1 | 1 |  | **2** | **3** | 2 | 1 |  | 1 | 4 |  | Moderate |
| S01009 | Baseline | 3 | 1 | 1 |  | 3 | 3 | 1 | 1 |  | 0 | 2 |  | Moderate |
|  | Day 270 | **1** | 1 | 1 |  | **2** | **2** | 1 | 1 |  | 0 | 3 |  | Moderate |
| S01010 | Baseline | 4 | 1 | 2 |  | 1 | 4 | 2 | 2 |  | 0 | 4 |  | Moderate |
|  | Day 270 | **2** | 1 | 2 |  | **0** | **3** | 2 | 2 |  | 0 | 4 |  | Moderate |
| S01011 | Baseline | 2 | 1 | 1 |  | 2 | 4 | 1 | 1 |  | 0 | 4 |  | Moderate |
|  | Day 270 | 2 | 1 | 1 |  | **1** | 4 | 1 | 1 |  | 0 | 4 |  | Moderate |
| S01012 | Baseline | 2 | 2 | 1 |  | 2 | 4 | 1 | 1 |  | 0 | 4 |  | Moderate |
|  | Day 270 | 2 | 2 | 1 |  | **1** | **3** | 1 | 1 |  | 0 | 4 |  | Moderate |
| S01013 | Baseline | 3 | 1 | 1 |  | 2 | 3 | 1 | 1 |  | 0 | 3 |  | Moderate |
|  | Day 270 | **2** | 1 | 1 |  | 2 | 3 | 1 | 1 |  | 0 | 3 |  | Moderate |

[1] 0:None 1:Symptoms below or at ankle bones 2:Symptoms up to the distal half of the calf 3:Symptoms up to the proximal half of the calf, including knee 4:Symptoms above knee (above the top of the patella)
[2] 0:None 1:Trips, catches, toes, slaps feet, shoe inserts 2:Ankle support or stabilization (AFOs) Foot surgery 3:Walking aids (cane, walker) 4:Wheelchair

[3] 0:None 1:Mild difficulty with buttons 2:Severe difficulty or unable to do buttons 3:Unable to cut most food 4:Proximal weakness (affect movements involving the elbow and above)

[4] 0:Normal 1:Decreased below or at ankle bones 2:Decreased up to the distal half of the calf 3:Decreased up to the proximal half of the calf, Including knee 4:Decreased above knee (above the top of the patella)

[5] 0:Normal 1:Reduced at great toe 2:Reduced at ankle 3:Reduced at knee (tibial tuberosity) 4:Absent at knee and ankle

[6] 0:Normal 1:4+, 4, or 4- on foot dorsiflexion or plantar flexion 2: ≤ 3 on foot dorsiflexion or ≤ 3 on foot plantar flexion 3:≤ 3 on foot dorsiflexion and ≤ 3 on plantar flexion 4:Proximal weakness

[7] 0:Normal 1:4+, 4, or 4- on intrinsic hand muscles 2: ≤ 3 on intrinsic hand muscles 3:≤5 on wrist extensors 4:Weak above elbow[8] 0:≥ 6mV (≥ 4mV) 1:4 ~ 5.9mV (2.8 ~ 3.9mV) 2: 2 ~ 3.9mV (1.2 ~ 2.7mV) 3:0.1 ~ 1.9mV (0.1 ~ 1.1mV) 4:None (None)

[8] 0:≥ 6mV (≥ 4mV) 1:4 ~ 5.9mV (2.8 ~ 3.9mV) 2: 2 ~ 3.9mV (1.2 ~ 2.7mV) 3:0.1 ~ 1.9mV (0.1 ~ 1.1mV) 4:None (None)
[9] 0:≥ 15 μV 1:10-14.9 μV 2: 5-9.9 μV 3:1-4.9 μV 4:< 1 μV

[10] Mild:≤10, Moderate:≤20, Severe≤36

Values in bold indicate score improvement.
