# Supplementary material for: An open-label single-arm phase 1/2a study to evaluate the safety and exploratory efficacy of a VM202 in patients with Charcot-Marie-Tooth disease 1A
Source: Orphanet J Rare Dis. 2026 Mar 5;21:148. doi: 10.1186/s13023-026-04252-2 (PMC13072572; doi:10.1186/s13023-026-04252-2)
Supplement: Supplementary file 2 — Supplementary Material 2 [file 13023_2026_4252_MOESM2_ESM.pdf]

Single Center, Open label, Repeat Intramuscular Administration, 270days, Phase I/2a Clinical Trial to Evaluate Safety and Tolerability of Investigational Product (Engensis: VM202) in Patients with Charcot-Marie-Tooth Disease subtype 1A (CMT1A)

Helixmith Co., Ltd.
21, Magokjungang 8-ro 7-gil, Gangseo-gu
Seoul 07794, South Korea

**Protocol No.**	:	**VMCMT-001**
**Investigational Product**	:	**Engensis:VM202**
**Version**	:	**V4.0**
**Version Date**	:	**2021. 06.07**

Confidential Statement
All information contained in this document is proprietary to Helixmith Co., Ltd and should be kept confidential unless written consent is provided. The contents of this clinical trial protocol may be disclosed only to the regulatory authorities, clinical trial investigators or personnel who are related to the Institutional Review Board (IRB) of the study center or the conduct of this clinical trial. This protocol cannot be used for any other purpose than the clinical trial.

Table of Contents

SIGNATURE PAGE 1

CONSENT FORM FOR INVESTIGATOR 2

SYNOPSIS 3

SUMMARY OF STUDY SCHEDULE 12

GLOSSARY OF ABBREVIATIONS 16

1 TITLE AND PHASE OF CLINICAL STUDY 19

1.1 TITLE OF CLINICAL STUDY: PHASE 1/2A 19

2 INFORMATION OF SPONSOR AND CRO, NAME AND TITLE OF PRINCIPAL INVESTIGATOR 20

2.1 SPONSOR 20

2.2 PRINCIPAL INVESTIGATOR AND OTHER CLINICAL STUDY PARTICIPANTS 20

2.3 CONTRACT RESEARCH ORGANIZATION 20

3 INTRODUCTION 21

3.1 INCIDENCE AND PREVALENCE OF CHARCOT-MARIE-TOOTH DISEASE 21

3.2 PATHOPHYSIOLOGY OF CHARCOT-MARIE-TOOTH DISEASE 21

3.3 LATEST TREATMENT METHODS FOR CHARCOT-MARIE-TOOTH DISEASE 23

3.4 LIMITATIONS OF EXISTING THERAPIES AND NECESSITY FOR NEW THERAPIES 25

3.5 TREATMENT OF CHARCOT-MARIE-TOOTH DISEASE USING HEPATOCYTE GROWTH FACTOR (HGF) 26

3.6 ENGENSIS; VM202 29

3.7 NONCLINICAL STUDY DATA 31

3.8 RESULTS OF PREVIOUSLY CONDUCTED CLINICAL STUDIES 34

3.8.1 Phase 1 Clinical Study in Patients with Critical Limb Ischemia 34

3.8.2 Phase 2 Clinical Study on Critical Limb Ischemia 36

3.8.3 Phase 1/2 Clinical Study on Painful Diabetic Peripheral Neuropathy 38
3.8.4 Phase 2 Clinical Study on Painful Diabetic Peripheral Neuropathy 39
3.8.5 Phase 1/2 Clinical Study on Amyotrophic Lateral Sclerosis 41
3.8.6 Phase 1 Clinical Study on Angina Pectoris 42
3.9 RATIONALE FOR ESTABLISHING DOSE OF INVESTIGATIONAL PRODUCT 43

4 GOOD CLINICAL PRACTICE 47

5 CLINICAL STUDY PLAN 48

5.1 PURPOSE OF CLINICAL STUDY 48
5.2 CLINICAL STUDY DESIGN 49
5.3 SUBJECTS 49
5.3.1 Target Number of Subjects and Rationale for Calculation 49
5.3.2 Inclusion Criteria 50
5.3.3 Exclusion Criteria 50
5.3.4 Assignment of Subject Numbers 52
5.4 CONTRAINDICATED MEDICATIONS 53
5.5 TERMINATION OF CLINICAL STUDY 58
5.5.1 Study Termination for Subjects 58
5.5.2 Subject Drop-out 58
5.5.3 Early Termination of Clinical Study 59
5.6 INFORMATION ABOUT AND MANAGEMENT OF INVESTIGATIONAL PRODUCT 61
5.6.1 Information About and Management of Investigational Product 61
5.6.2 Dose, Administration Route, and Administration Method 61
5.6.3 Labeling and Packaging 62
5.6.4 Handling and Preparation of Investigational Product 63
5.6.5 Accountability Management, Collection and Disposal of Investigational Product 63
5.7. MANAGEMENT OF CORONAVIRUS DISEASE-19 (COVID-19) 64

5.7.1 Selection and enrollment of clinical study subjects 64
5.7.2 Management of clinical study subjects 64
5.7.3 Records of Coronavirus Disease-19 (COVID-19) 65
5.8 Retrospective Biomarker Study 65
5.8.1 Purpose of the study 65
5.8.2 Method of the study 65
5.8.3 Methods for anonymizing human materials and measures to protect personal information 66
6 CLINICAL STUDY PROCEDURES AND ASSESSMENTS 67
6.1 Visit Schedule and Observation Items 67
6.1.1 Visit 1 (screening, from Day -30 to Day -1) 71
6.1.2 Visit 2 (Administration 1, Day 0) 76
6.1.3 Visit 3 (Second Administration, Day 14 ± 5) 81
6.1.4 Day 30 ± 7 (shall be performed only if follow-up testing is required) 84
6.1.5 Visit 4 (Third Administration, Day 90 ± 7) 87
6.1.6 Visit 5 (Fourth Administration, Day 104 ± 7) 92
6.1.7 Visit 6 (Interim Visit, Day 180 ± 7) 95
6.1.8 Visit 7 (Termination Visit, Day 270 ± 7) and Early Termination 98
6.1.9 Unscheduled Visits 103
6.2 Assessment Items 105
7 ADVERSE EVENT 106
7.1 Definition 106
7.2 Expected Adverse Event 107
7.2.1 Adverse events that may be caused by Charcot-Marie-Tooth, the underlying disease 108
7.2.2 Adverse events that may be caused by administration of investigational product 109
7.2.3 Summary of AEs that occurred in clinical studies on other indications using the investigational product 111
7.3 Precautions 114

7.4 CRITERIA AND METHODS FOR ASSESSING AEs 114
7.4.1 Causality with investigational product 115
7.4.2 Severity of AE 116
7.4.3 Reporting, collection, and recording of AEs 117
7.4.3.1 Collection, and recording of AESI(Adverse Events of Special Interest) 118
7.4.4 Reporting and recording of SAEs 118
7.4.5 Handling AE 120
7.4.6 Handling related to investigational product 121
7.4.7 Pregnancy report 122
8 STATISTICAL METHOD 123
8.1 ANALYSIS SET 123
8.1.1 Subject Set to Be Included in Safety and Tolerability Assessment Analyses: Safety set 123
8.1.2 Subject Set to Be Included in Efficacy Assessment Analysis: Intention-to-treat (ITT) set 123
8.1.3 Subject Set to Be Included in Efficacy Assessment Analysis: Per-protocol (PP) set 123
8.1.4 Subgroup Analysis Population 123
8.2 HANDLING OF MISSING VALUES 124
8.3 CLASSIFICATION OF SUBJECTS 124
8.4 STATISTICAL ANALYSIS METHOD 124
8.4.1 General Principles of Statistical Analysis 125
8.4.2 Basic Information on Subjects and Disease 125
8.4.3 Primary Endpoints 125
8.4.4 Secondary Endpoints 126
9. DOCUMENT MANAGEMENT 128
9.1 CASE REPORT FORM 128
9.2 RECORDING AND COLLECTING 128
9.3 ACCESS TO, PROTECTION OF, AND STORAGE OF RECORDS 129

10 QUALITY CONTROL AND ASSURANCE 131

11 INFORMED CONSENT 132

12 APPROVAL OF CLINICAL STUDY PROTOCOL 133

13 CONFIDENTIALITY OF SUBJECTS' RECORDS 134

14 MONITORING OF CLINICAL STUDY 135

15 MEASURES FOR PROTECTION OF SUBJECTS' SAFETY 136

16 AMENDMENT OF CLINICAL STUDY PROTOCOL 137

17 CLINICAL STUDY REPORT (CSR) 138

18 PRESENTATION AND PUBLICATION OF CLINICAL STUDY REPORT 139

19 REFERENCES 140

APPENDIX 1. INFORMATION OF SPONSOR, NAME AND TITLE OF PRINCIPAL INVESTIGATOR

APPENDIX 2. CONTRAINDICATED DRUGS

APPENDIX 3. ADMINISTRATION METHOD OF INVESTIGATIONAL PRODUCT

APPENDIX 4. CMTNS-V2

APPENDIX 5. ONLS LEG SCALE

APPENDIX 6. 10-METER WALK TEST (10MWT)

APPENDIX 7. NERVE CONDUCTION STUDY (NCS)

Appendix 8. Algorithm for Causal Relationship Assessment Method

Appendix 9. Summary of Adverse Events That Occurred in Previous Clinical Studies

List of Tables

Table 1 Plasmid component factors 31

Table 2 Summary of Clinical Studies Conducted on Engensis (VM202) 34

Table 3. Toxicity study results 44

Table 4. Administration dose per visit in past clinical studies 45

Table 5. Contraindicated medications 53

Table 6. Information about and storage conditions of investigational product 61

Table 7. Total number of investigational product vials, doses, and injections per subject at each visit 62

Table 8. Number of injections and administration dose per target muscle (injection site) 62

Table 9. Neurologic exam items and procedures for CMTNS-v2 measurement 74

Table 10. Neurologic exam items and procedures for CMTNS-v2 measurement 78

Table 11. Neurologic exam items and procedures 83

Table 12. Neurologic exam items and procedures 86

Table 13. Neurologic exam items and procedures 90

Table 14. Neurologic exam items and procedures 93

Table 15. Neurologic exam items and procedures 98

Table 16 Adverse events that may be caused by Charcot-Marie-Tooth 108

Table 17 Adverse events caused by intramuscular injection [78-83] 110

Table 18 Predicted adverse events described in the Investigator's Brochure 110

Table 19 AEs that were reported as having causality in CLI phase1 111

Table 20 AEs that were reported as having causality in CLI phase2 112

Table 21 AEs that were reported as having causality in painful DPN phase1/2 113

Table 22. AEs that were reported as having causality in painful DPN phase 2 113

Table 23 AEs that were reported as having causality in ALS phase 1/2 114

Table 24 Causality with investigational product 115

Table 25 General classification in NCI-CTCAE V5.0 117

List of Figures

Figure 1. Study schematic diagram 6

Figure 2 Pathophysiology of CMT 23

Figure 3 Molecular mechanism of neuroprotective effect of HGF 29

Figure 4 Structure of Engensis (VM202) 30

Figure 5 Engensis (VM202) plasmid map 31

Figure 6 Toxicity study data (see IB) 33

Figure 7. A schematic diagram of clinical study 49

Figure 8. Summary of investigational product packaging 63

Figure 9 AEs that may occur during clinical study 108

Establishment and Amendments History

No	Version No.	Version Date	Amendment Details
1	Version 1.0	31/JAN/2020	Establishment
2	Version 1.1	30/Mar/2020	Amendment
3	Version 1.2	10/APR/2020	Amendment
4	Version 1.3	11/MAY/2020	Amendment
5	Version 1.4	22/JUN/2020	Amendment
6	Version 1.5	02/JUL/2020	Amendment
6	Version 2.0	31/JUL/2020	Amendment
7	Version 2.1	06/AUG/2020	Amendment
8	Version 2.2	05/OCT/2020	Amendment
9	Version 3.0	04/FEB/2021	Amendment
10	Version 4.0	04/JUN/2021	Amendment

SIGNATURE PAGE

Sponsor's Approval:

The VMCMT-001 clinical study protocol has been approved by Helixmith Co., Ltd.

Responsible Medical Officer:

Eun Seon Shin

Clinical Development

Helixmith Co., Ltd.

Sponsor's Authorized Officer:

Review:

______________________________	______________________________
Eun Seon Shin<br>Clinical Development<br>Helixmith Co., Ltd.	Date

Approval:

______________________________	______________________________
Young Joo Park, PhD<br>Clinical Development<br>Helixmith Co., Ltd.	Date

Consent Form for Investigator

I shall perform all of my responsibilities while conducting this clinical study at this institution, and I hereby agree to comply with the following details:

- I am well aware that this clinical study protocol is confidential. I shall not disclose the information contained in this document to anyone without a prior written permission of the sponsor, Helixmith Co., Ltd., except for the purpose of conducting this clinical study or obtaining approval for this clinical study from the Institutional Review Board or other committees and I hereby agree to the above.
- I have gained full knowledge of the details of this clinical study protocol. I shall conduct this clinical study in accordance with the approved clinical study protocol, details of protocol amendments, the International Conference on Harmonisation-Good Clinical Practice (ICH-GCP), all requirements of regulatory agencies related to clinical studies, and domestic laws.
- Except for the purpose of taking actions against emergency situations that have occurred in the subjects, I shall not arbitrarily change the details of this clinical study protocol without a prior written permission of Helixmith Co., Ltd. or a preliminary review and written approval of the Institutional Review Board or another separate committee.
- I have gained full knowledge of the details of the investigator's brochure.
- For the duration of the clinical study, which has been agreed upon with Helixmith Co., Ltd., I shall conduct and complete this clinical study in accordance with regulations. I constitute the authorized person who is qualified to conduct this clinical study properly and safely, and shall set up the relevant equipment.
- I shall ensure that appropriate training in how to conduct the study, the clinical study protocol, and the duties of each investigator is performed for all investigators related to this clinical study at this institution. If a previous investigator is to delegate his/her duties to a new investigator, a letter of delegation shall be provided to Helixmith Co., Ltd.
- If required for the protection of rights and interests of the subjects enrolled in the clinical study, I am well aware that Helixmith Co., Ltd., the institution, or the principal investigator may temporarily stop recruiting subjects or terminate the clinical study.

Principal Investigator (Name) ______________________________

Institution/Position ______________________________

Address ______________________________

Signature/Date ______________________________

Synopsis

**Sponsor**	Helixmith Co., Ltd.<br>21, Magokjungang 8-ro 7-gil, Gangseo-gu, Seoul 07794, South Korea
Study title	*A Single-Center, Open-Label, Phase 1/2a Clinical Study to Evaluate the Safety and Tolerability for 270 Days Following Repeated Intramuscular Administration of Investigational Product (Engensis: VM202) in Patients With Charcot-Marie-Tooth Disease Subtype 1A (CMT1A)*
**Study design**	Single center, repeat dose, open label, phase I/IIA clinical study
**Phases of study**	Phase I/IIA clinical study
**Principal investigator, institution**	**Principal investigator:**<br>Prof. Byung Ok Choi / Neurology<br>**Institution:**<br>Samsung Medical Center / 81 Irwon-ro, Gangnam-gu, Seoul, South Korea
**Duration of study**	48 months after clinical study protocol approval by the Korean Ministry of Food and Drug Safety and IRB (Study completion: date of last patient's last visit)
**Investigational product**	**Code name:** Engensis (VM202)<br>**Dosage form and appearance**: White (or close to white) lyophilized powder contained in a colorless, clear vial; clear liquid when dissolved with water for injection<br>**Storage of investigational product**: refrigerated storage (2-8 ℃)
**Study subjects**	Charcot-Marie-Tooth disease subtype1A (CMT1A) patients with mild-to-moderate disease severity assessed by Charcot-Marie-Tooth Neuropathy Score version 2 (CMTNS-v2) with a score >2 and ≤20;
**Number of subjects**	12
**Purpose of study**	To assess the safety and tolerability of the investigational product (VM202) injected in the weakened lower limb muscles of CMT1A patients

**Inclusion criteria**	**<u>Subjects enrolled in this clinical study must satisfy all of the following inclusion criteria:</u>**<br>1) Male or female, aged 19 to 65 years;<br>2) Patients with confirmed diagnosis of CMT1A by genetic testing;<br>3) Patients with mild-to-moderate severity assessed by Charcot-Marie-Tooth Neuropathy Score version 2 (CMTNS-v2) with a score > 2 and ≤ 20;<br>4) Individuals with lower limb muscle weakness with minimum dorsiflexion or more;<br>5) Individuals who voluntarily consent to participate in this study and sign the IRB-approved informed consent form after understanding a description on the characteristics of this clinical study prior to all screening tests;<br>6) Individuals who can comply with the requirements in the clinical study;<br>7) In case of females of childbearing potential, those who test negative in a urine or serum pregnancy test at screening;<br>8) Individuals who practice <u>medically approved contraceptive methods*</u> throughout the clinical study.<br>* Definition<br>- Drugs: Oral contraceptives, skin patches, or progestin formulations (implants or injections)<br>- Barrier methods: Condoms, diaphragms, intrauterine devices (IUDs), vaginal suppositories<br>- Abstinences: Complete abstinence (However, periodic abstinence (e.g., calendar method, ovulation method, and sympto-thermal method) and self-restraint are not considered as acceptable methods of contraception.)
**Exclusion criteria**	**<u>Subjects will be excluded from this clinical study if any one of the following criteria is met:</u>**<br>1) Patients with significant respiratory, circulatory, renal, gastrointestinal, hepatic, endocrine, hematologic, psychiatric disorders or other severe diseases, or alcohol or drug addiction who may develop safety issues or cause confusion in the interpretation of the clinical study results as determined by the principal investigator;<br>2) Patients with other neuromuscular diseases or neuropathy-inducing factors: Patients with chronic alcohol addiction, undergoing anticancer chemotherapy, or taking neurotoxic drugs;<br>3) Patients diagnosed with diabetes;<br>4) Patients diagnosed with inflammatory bowel disease;<br>5) Patients with a history of stroke or cerebral ischemic attack within 12 months prior to the screening date;<br>6) Patients with a history of coronary artery disease, such as myocardial infarction

and unstable angina pectoris within 12 months prior to the screening date;

7) Morbidly obese patients with body mass index (BMI) ≥ 37;

8) Patients who underwent orthopedic surgery (corrective surgery for bone and ligament, artificial joint implantation, osteosynthesis, osteotomy, arthroscopic surgery) in the lower limbs within 6 months prior to the screening date;

9) Patients who may be affected by the muscle strength measurement test due to ankle contracture or surgery;

10) Patients with uncontrolled hypertension (if systolic blood pressure is ≥ 160 mmHg or diastolic blood pressure is ≥ 100 mmHg at screening);

11) Patients or patient's immediate family members (parents, siblings, offspring) with a history of malignant tumors within the last 5 years prior to the screening date, excluding basal cell carcinoma or squamous cell carcinoma that occurs on the skin (if it is determined that there is no possibility of relapse after resection), or with a family history of familial adenomatous polyposis (FAP) or hereditary nonpolyposis colorectal cancer (HNPCC);

12) Patients who have not completed a national cancer screening program applicable to their sex and age (if it cannot be confirmed that the relevant test was received at a national cancer screening center or a recognized screening center).

However, if it is confirmed that the relevant test was received at a national cancer screening center or a recognized screening center during the screening period, and that the results were within normal range, the patients may participate in the clinical study;

Common to males and females: If a patient is ≥ 50 years of age, the results of a colonoscopy within 5 years prior to the screening must be determined as being within normal range, and if adenomatous polyps are evident, the results of a colonoscopy within 1 year must be determined as being within normal range (inflammatory polyps or hyperplastic polyps are included in the normal range). If a patient is ≥ 40 years of age, the results of a gastroscopy within 2 years prior to the screening must be within normal range. If a patient is ≥ 54 years of age and has a 30 pack-year history of smoking or more, the results of a low-dose chest CT within 2 years prior to the screening must be within normal range. In case of liver cancer, carriers of hepatitis B or hepatitis C virus and patients with hepatic cirrhosis fall under the exclusion criteria.

Females: For females ≥ 40 years of age, normal range findings must be confirmed in a mammogram within 2 years. For females ≥ 20 years of age, normal range findings must be confirmed in a Pap smear within 2 years.

	13) Patients diagnosed with active pulmonary tuberculosis;<br>14) Patients with HBV or HCV;<br>15) Patients who test positive in human immunodeficiency virus (HIV) antibody test;<br>16) Patients in an immunosuppressive state due to treatments such as immunosuppressants, chemotherapy, and radiotherapy;<br>17) Patients with a history of mental disease within 6 months prior to the screening date, which may interfere with participation in the study;<br>18) Patients who must take medications, that are known to have significant drug interactions within 14 days after the first administration of the investigational product or deemed unsuitable by the investigator's judgment;<br>19) Individuals who participated in another clinical study* within 6 months before the time of screening<br>* Definition<br>• Drug: Those who participated in another clinical study within 6 months before the time of screening shall be excluded;<br>• Medical device: Those who participated in a noninvasive clinical study may participate in this clinical study if the principal investigator determines that the safety or pharmacodynamic assessment will not be affected<br>20) Individuals who have shown significant adverse events such as hypersensitivity reactions to the investigational product<br>21) Pregnant or breastfeeding females<br>22) Other individuals determined ineligible by the principal investigator to participate in the clinical study due to other reasons including clinical laboratory test results
**Study design and study method**	The procedures and methods of this clinical study are as follows:<br>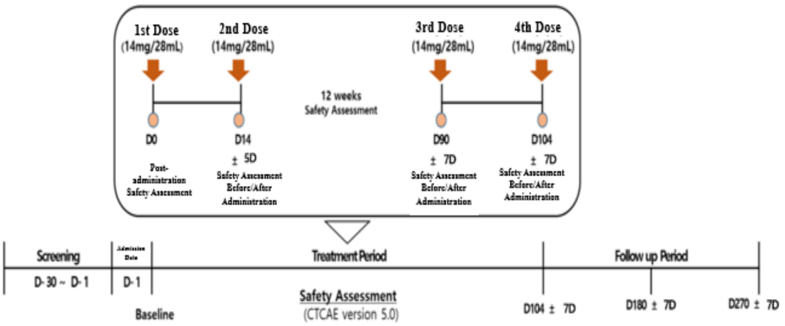<br><br>**Figure 1. Study schematic diagram**

If subjects who have voluntarily signed the study consent form are enrolled in the clinical study, they are tested for eligibility to this study during the screening period. Those who meet the inclusion/exclusion criteria are given a total of 4 doses of the investigational product for 104 days (2 doses over 2 weeks, followed by 3-month safety and tolerability assessment, and then another 2 doses over 2 weeks). The drug's safety and tolerability are assessed at each visit, and efficacy is assessed at the 2nd visit (baseline, Day 0) and 7th visit (last visit, Day 270).

Although this study is a phase 1/2a, considering that CMT1A (Charcot-Marie-Tooth) disease is a rare disease that has no treatment yet, this clinical study is designed to conduct an assessment of clinically meaningful factors in relation to efficacy.

Administration of investigational product

Administration of investigational product: Intramuscular injection

Preparation of investigational product:

Each vial contains 2.5 mg of "pCK-HGF-X7," the main ingredient of the investigational product, in a sterilized/lyophilized condition. The investigator uses 5.0 mL of water for injection to dilute the investigational product (0.5 mg/mL) and then administers a total of 56 intramuscular injections into three muscles each in the left and right lower limbs of the study subject. The quantity of the investigational product, the number of injections, dosage, and target muscles by each subject at each visit are shown in Table 4 and Table 5 below.

Caution:

Each diluted vial shall be used for one subject only.

Using thin needles suitable for intramuscular injection (e.g., 29 gauge, 1/2 inch or 1inch in length according to muscle type and subcutaneous fat thickness), injections shall be evenly distributed on the target muscle as shown below, avoiding the fascia.

Table 4. Total number of vials, dosage, and number of injections per visit

Number of vials per visit	Number of injections per visit	Total dose per visit
7	56	14mg/28mL

Table 5. Number of injections and dosage for each muscle (injection site)

Target muscle	Dosage (mg) per target muscle (number of	Total dosage

		injections per muscle: left/right)				(mg), (total number of injections: left/right)
		1st administration	2nd administration	3rd administration	4th administration	
		D0	D14	D90	D104	
Lower leg	Peroneus longus	3, (6/6)	3, (6/6)	3, (6/6)	3, (6/6)	12, (24/24)
	Gastrocnemius	6, (12/12)	6, (12/12)	6, (12/12)	6, (12/12)	24, (48/48)
	Tibialis anterior	5, (10/10)	5, (10/10)	5, (10/10)	5, (10/10)	20, (40/40)
**Final dosage (number of injections: left/right)**		**14, (28/28)**	**14, (28/28)**	**14, (28/28)**	**14, (28/28)**	**56 (112/112)**

Primary endpoint

Safety and tolerability are evaluated in the following items, including adverse events collected from study subjects following the administration of the investigational product (VM202).

*** Safety and tolerability assessment**

(1) Adverse event

- All adverse events that manifest after administration of the investigational product shall be collected.
- At Visits 3, 4, and 5 (2nd, 3rd, and 4th administration sessions of the investigational product), adverse events shall be assessed and collected before and after administration of the investigational product.

(2) Laboratory tests (complete blood cell count/general blood chemistry/urinalysis tests)

(3) Vital signs

Secondary endpoint

Considering that CMT1A (Charcot-Marie-Tooth) disease is a rare disease that has no treatment yet, this clinical trial is designed to evaluate clinically meaningful factors in relation to efficacy.:

(1) Change in severity of disease

- CMTNS-v2 (Charcot-Marie-Tooth Neuropathy Score version 2)
- FDS (functional disability scale)

(2) Changes in lower limb function

- ONLS (overall neuropathy limitation score) leg scale
- 10MWT (10-meter walk test)

(3) Changes in fatty infiltration level of lower limb muscles

- MRI leg

(4) Nerve regeneration potential

- CMAP (compound motor nerve action potential)

	- SNAP (compound sensory nerve action potential)<br>- NCV (nerve conduction velocity)<br>(5) HGF antibody generation by VM202
**Statistical analysis of primary endpoint**	**Definition of subjects for primary endpoint analysis**<br>**Safety set**<br>Of all subjects who were decided suitable during screening, the analysis includes those who were administered the investigational product and can be assessed for safety. The study subjects included in the safety and tolerability analysis are analyzed based on information on the investigational product that was actually administered. Furthermore, the safety set is analyzed for demographic data (gender, age, etc.) and background factors (medical history, previous drug treatment, etc.)<br><br>**Statistical analysis method for primary endpoint**<br>**(1) Adverse event:**<br>: Summary and analysis of adverse events are conducted on Treatment-Emergent Adverse Events (TEAEs) that occur after the administration of the investigational product.<br>Frequency and percentage of TEAEs, adverse drug reactions (ADRs), and serious adverse events (SAEs), occurring after investigational product administration, are provided.<br>TEAEs, ADRs, and SAEs are coded using MedDRA (Medical Dictionary for Regulatory Activities, latest version) according to the System Organ Class (SOC) and Preferred Terminal (PT). The number of subjects with coded adverse events, the occurrence rate, and the number of the cases are presented.<br><br>**(2) Laboratory tests and vital signs**<br>: For continuous variables, descriptive statistics (average, standard deviation, median value, minimum value, maximum value) are presented for each visit. For categorical variables, frequency and percentage are presented. For each visit, frequency and percentage of normal changes, not clinically significant (NCS) abnormal changes, and clinically significant (CS) changes are presented for each visit, and a list of subjects assessed as abnormal (CS) for each visit is presented.
**Statistical analysis of secondary endpoint**	**Definition of subjects for secondary endpoint analysis**<br>**Intention-To-Treat (ITT)**<br>Regardless of whether the protocol was breached or the visit schedule was adhered to, all subjects who have been administered with the investigational product once or more and can be assessed for efficacy are included in the ITT.<br><br>**Per-Protocol Set (PPS)**<br>PPS applies to the ITT subjects who have completed the study in accordance with

the protocol without any major protocol violation, and the subjects corresponding to the following are defined as PPS. Compliance with No. ① eligibility shall be determined in a data review meeting prior to datalock.

① Subjects who complied with the inclusion/exclusion criteria (eligible patients);
② Subjects who completed all visits.

Statistical analysis method for secondary endpoint

Although this study is phase 1/2a that is not intended for efficacy assessment, considering that this is a rare disease that has no treatment yet, assessment will be conducted on clinically meaningful factors in relation to efficacy.

(1) Severity of disease

Measured by CMTNS-v2 (Charcot-Marie-Tooth Neuropathy Score version 2) and FDS (functional disability scale)

(2) Walking function

Change in walking function is assessed by ONLS (overall neuropathy limitation score) leg scale, and 10MWT (10-meter walk test).

(3) Fatty infiltration rate in lower limb muscle

Using leg MRI scans, the degree of fatty infiltration of the leg muscle that has received the investigational product is measured and evaluated as the fat content value (%) at each muscle level.

(4) Potential of nerve regeneration

Nerve conduction tests are conducted, including CMAP (compound motor nerve action potential), SNAP (compound sensory nerve action potential), and NCV (nerve conduction velocity).

(5) Formation of HGF antibody caused by Engensis (VM202)

The formation of anti-HGF Ab in the blood is checked. Preliminary assessment is made of whether antibody production is correlated between the subject group who are considered to have increased muscle mass and improved function and those who are not. The summary table presents the frequency and ratio of antibody generation in the blood.
However, if no antibody in the blood is produced in any single subject, no results are presented.

For the secondary endpoint, for values before and after treatment, continuous variables present average, standard deviation, median value, minimum value, and

	maximum value; categorical variables present frequency and percentage. To compare differences of values before and after treatment, continuous variables use a paired t-test or Wilcoxon sign rank test; categorical variables use McNemar's test.
**Small group analysis**	When conducting the adverse events and efficacy assessments, small group analysis is carried out considering the following items:<br><br>• Gender (male, female)<br>• Age (≤ median, > median)<br>• Baseline BMI (≤ median, > median)<br>• Presence or absence of medical history<br>• Presence or absence of concomitant medications<br>• Disease severity (CMTNS v2) (mild, moderate)<br><br>If the number of subjects that fit into the above subgroups is small in each group, the analysis shall not be conducted on that subgroup. For example, if male subjects are less than 30% of the total subjects, no analysis is performed on gender and whether to analyze shall be determined in a data review meeting prior to datalock.
**Retrospective biomarker study(To be conducted separately)**	Changes in peripheral neuropathy biomarker (p62, p75, NCAM)<br>- Serum p62 (p62/sequestosome-1) concentration<br>- Serum p75 (p75 neurotrophin receptor) concentration<br>- Serum NCAM (neural cell adhesion molecule 1) concentration

Summary of study schedule

Schedule	Day -30 to Day -1	Day -1	Day 0		Day 14 ± 5		Day 30 ± 7	Day 90 ± 7		Day 104 ± 7		Day 180 ± 7	Day 270 ± 7	Early Termination	Unscheduled Visit
Visit No.	Visit 1	Visit 2 (Hospitalization)			Visit 3		Outpatient follow-up if abnormal test results are present on Day 14 (Second Administration)*	Visit 4		Visit 5		Visit 6	Visit 7		
Specific Notes	Screening	Hospitalization Day[15)]	First Administration		Second Administration			Third Administration		Fourth Administration		Outpatient Follow-up	Outpatient Follow-up		
			Before Administration	After Administration[16)]	Before Administration	After Administration[16)]		Before Administration	After Administration[16)]	Before Administration	After Administration[16)]				
Informed consent form	X														
Subject background survey[1)]	X														
Medical history survey[2)]	X														
Physical examination[3)]	X														
Body measurement (weight measurement)[4)]	X		X		X		X*	X		X		X	X	X	X
Virus serology test[5)]	X														
Complete blood cell count and general blood chemistry tests[6)]	X		X		X		X*	X		X		X	X	X	X
Retrospective biomarker study[7)]			X					X				X	X	X	
Chest X-ray (PA)[8)]	X														
Urinalysis (U/A)[9)]	X		X		X		X*	X		X		X	X	X	X
Urine or serum pregnancy test[10)]	X														

Schedule	Day -30 to Day -1	Day -1	Day 0		Day 14 ± 5		Day 30 ± 7	Day 90 ± 7		Day 104 ± 7		Day 180 ± 7	Day 270 ± 7	Early Termination	Unscheduled Visit
Visit No.	Visit 1	Visit 2 (Hospitalization)			Visit 3		Outpatient follow-up if abnormal test results are present on Day 14 (Second Administration)*	Visit 4		Visit 5		Visit 6	Visit 7		
Specific Notes	Screening	Hospitalization Day[15]	First Administration		Second Administration			Third Administration		Fourth Administration		Outpatient Follow-up	Outpatient Follow-up		
			Before Administration	After Administration[16]	Before Administration	After Administration[16]		Before Administration	After Administration[16]	Before Administration	After Administration[16]				
Electrocardiogram[11]	X														
Anti-HGF Ab			X										X	X	
Vital signs	X	X[15]	X	X	X	X	X*	X	X	X	X	X	X	X	X
Concomitant medications survey[12]	X		X		X		X*	X		X		X	X	X	X
Neurologic exam	X		X		X		X*	X		X		X	X	X	
CMTNS-v2	X		X										X	X	
MRI leg[13]			X										X	(X)	
FDS, ONLS (leg), 10MWT			X					X				X	X	X	
CMAP, SNAP, NCV			X										X	X	
Use of assistive device	X												X	X	
IP administration**			X		X			X		X					
Adverse event assessment[14]				X	X	X	X*	X	X	X	X	X	X	X	X

* PI shall determine whether test results are abnormal and whether Day 30 follow-up testing is required.

** IP administration: Administration shall be performed after proceeding with all pre-administration assessment items.

1) Subject background survey: A survey shall be performed on demographic information and history of alcohol and tobacco use, etc.

2) Medical history survey: A survey shall be performed on medical history within 6 months before Visit 1 (screening). However, medical history/treatment history related to cancer shall

be surveyed regardless of the time period. Clinically significant medical conditions or abnormalities observed during the period from the obtainment of the informed consent form until the administration of the investigational product shall be deemed as medical history. It shall be surveyed whether the national cancer screening (gastric cancer, colon cancer, liver cancer, lung cancer, cervical cancer, breast cancer) examinations relevant to the patient's age are taken and the results are within the normal range. In case of cervical cancer, if it cannot be confirmed that the national cancer screening examinations were taken within 2 years and the results are within the normal range, an examination and a pap smear shall be performed at the institution to verify normal range results.

3) Physical examination: Information shall be collected for examination items consisting of external appearance, skin, head/neck, chest/lungs, heart, abdomen, urinary/reproductive system, limbs, musculoskeletal system, nervous system, lymph nodes, and other items.
4) Body measurement (weight measurement): Height, weight, and BMI shall be measured. Height and BMI shall be measured only at screening (Visit 1).
5) Virus serology test: HIV, HBsAg, Anti-HBs, Anti-HCV
6) Complete blood cell count and general blood chemistry tests: The laboratory test items are as follows:
   - Complete blood cell count: WBC, RBC, Hb, Hct, MCV, MCH, MCHC, PLT, ESR, MPV, differential count of WBC (Band neutrophil, Segmented neutrophil, Eosinophil, Basophil, Lymphocyte, Monocyte)
   - General blood chemistry test: total protein, albumin, globulin, A/G ratio, cholesterol, total bilirubin, AST, ALT, fasting glucose, BUN, creatinine, estimated GFR, Ca^{2+}, phosphate, Na^{+}, K^{+}, Cl^{-}, CRP, triglyceride, HDL-cholesterol, LDL-cholesterol
7) Retrospective biomarker study : Blood samples are collected at Day 0, Day 90, Day 180 and Day 270 (or early termination visit) for retrospective biomarker study of CMT. Collected samples will be processed according to detailed procedure in a separate protocol. CMT
8) Chest X-ray (PA): At Visit 1 (screening), chest PA X-ray shall be performed to verify whether active tuberculosis is present. The results within 1 month (30 days) before Visit 1 may be used.
9) Urinalysis: The laboratory test items are as follows:
   - Color, turbidity, specific gravity, pH, albumin, glucose, ketones, bilirubin, blood, urobilinogen, nitrite, leukocyte esterase, microscopy (RBC, WBC, casts)
10) Urine or serum pregnancy test At Visit 1 (screening), urine or serum pregnancy tests shall be performed in females of childbearing potential (from post-menarche females to females ≥ 50 years of age within 1 year of menopause, or from post-menarche females to females < 50 years of age within 2 years of menopause). However, patients with surgical menopause (hysterectomy, bilateral oophorectomy, etc.) or who underwent sterilization surgery (bilateral tubal ligation, bilateral tubectomy) may be excluded. Menopause refers to the state after 1 year of amenorrhea.
11) Electrocardiogram: The electrocardiogram to be performed at Visit 1 (screening) may use results within 4 weeks before Visit 1 (screening).
12) Concomitant medications survey: All medications and treatments administered within 6 months before Visit 1 (screening) shall be surveyed. Previous medications and previous treatments shall be defined as all previously collected medications and treatments before Visit 2 (first administration of investigational product). Concomitant medications and treatments shall refer to all medications that have been administered at least once starting from Visit 2 (first administration of investigational product) and throughout the clinical study. The categories of collected medications and treatments shall follow this definition. Whether the medications and treatments being administered should be continued shall be

investigated at each visit.

13) MRI leg: The muscles of lower limbs shall be imaged, and the fatty infiltration level of the leg muscles injected with the investigational product shall be measured and evaluated as fat content value (%) at one level for each muscle. Considering the schedule, etc., of the institution, it shall be performed optionally at the early termination visit, and if it is not performed, the reasons shall be recorded in the case report form.

14) Adverse event assessment: At Visits 3, 4, and 5, an assessment of adverse events that have occurred since the last visit shall be performed prior to administering the investigational product, and localized adverse events shall be assessed at 2 ± 1 hours after administering the investigational product, as well as on the day after administration.

15) Hospitalization: Subjects shall be hospitalized on the day before administration of the investigational product and their vital signs shall be measured. If they are not hospitalized, vital signs may be omitted, and the hospitalization day in the case report form shall be recorded the same as the test day prior to administration.

16) After administration: Vital signs and adverse events shall be assessed at 2 ± 1 hours after administering the investigational product. The presence or absence of localized adverse events shall be verified on the day after administering the investigational product.

Glossary of Abbreviations

**AE**	Adverse Event
**AESI**	Adeverse Events of Special Interest
**AFO**	Ankle Foot Orthosis
**ALS**	Amyotrophic Lateral Sclerosis
**ALT**	Alanine Transaminase (SGTP)
**Anti-HCV**	Hepatitis C Antibodies
**AST**	Aspartate Transaminase (SGOP)
**BMI**	Body Mass Index
**BUN**	Blood Urea Nitrogen
**CBC**	Complete Blood cell Count
**cDNA**	Complementary Deoxynucleic Acid
**CLI**	Critical Limb Ischemia
**CMAP**	Compound Muscle Action Potential
**CMT**	Charcot-Marie-Tooth disease
**CMT1A**	Charcot-Marie-Tooth disease subtype 1A
**CMTNS-v2**	Charcot-Marie-Tooth Neuropathy Score Version 2
**COX-1**	Cyclooxygenase-1
**COX-2**	Cyclooxygenase-2
**CRF**	Case Report Form
**CRO**	Contract Research Organization
**DM**	Data Management
**DMP**	Data Management Plan
**DNA**	Deoxyribonucleic Acid
**DPN**	Diabetic Peripheral Neuropathy
**DSMB**	Data Safety Monitoring Board
**EDC**	Electronic Data Capture
**EKG**	Electrocardiogram
**FDS**	Functional Disability Scale
**GCP**	Good Clinical Practice
**HbcAb**	Hepatitis B core antibody
**HbsAb**	Hepatitis B surface antibody
**HbsAg**	Hepatitis B surface antigen
**HBV**	Hepatitis B Virus
**HCG**	Human Chorionic Gonadotrophin
**HCT**	Hematocrit

**HCV**	Hepatitis C Virus
**HED**	Human Equivalence Dose
**Hgb**	Hemoglobin
**HGF**	Hepatocyte Growth Factor
**HIV**	Human Immunodeficiency Virus
**HMSN**	Hereditary Motor and Sensory Neuropathy
**HTLV**	Anti-Human T-Cell Lymphotropic Virus
**IHD**	Ischemic Heart Disease
**IL-6**	Interleukin-6
**IND**	Investigational New Drug
**IRB**	Institutional Review Board
**ISR**	Injection Site Reaction
**ITT**	Intent-to-Treat
**KGCP**	Korea Good Clinical Practice
**LOCF**	Last Observation Carried Forward Analysis
**MCH**	Mean Corpuscular Hemoglobin
**MCHC**	Mean Corpuscular Hemoglobin Concentration
**MCV**	Mean Corpuscular Volume
**MedDRA**	Medical Dictionary for Regulatory Activities
**MPV**	Mean Platelet Volume
**MRI**	Magnetic Resonance Imaging
**mRNA**	Messenger Ribonucleic Acid
**NCI-CTCAE**	National Cancer Institute-Common Terminology Criteria for Adverse Events
**NCV**	Nerve Conduction Velocity
**NCAM**	Neural cell adhesion molecule 1
**NOAEL**	No-Observed-Adverse-Effect Level
**ONLS**	Overall Neuropathy Limitation Scale
**PA**	Posterior to Anterior
**PNS**	Peripheral Nervous System
**PP**	Per Protocol
**PT**	Preferred Term
**p62**	p62/sequestosome-1
**p75**	p75 Neurotrophin receptor
**RBC**	Red Blood Cell
**SAE**	Serious Adverse Event
**SNAP**	Sensory Nerve Action Potential
**SOC**	System Organ Class
**SOP**	Standard Operating Procedure

**TEAE**	Treatment-Emergent Adverse Event
**TNF**	Tumor Necrosis Factor
**10MWT**	10-meter walk test
**WBC**	White Blood Cell

1 Title and Phase of Clinical Study

1.1 Title of Clinical Study: Phase 1/2a

Single Center, Open label, Repeat Intramuscular Administration, 270days, Phase I/2a Clinical Trial to Evaluate Safety and Tolerability of Investigational Product (Engensis: VM202) in Patients with Charcot-Marie-Tooth Disease subtype 1A (CMT1A)

2 Information of Sponsor and CRO, Name and Title of Principal Investigator

2.1 Sponsor

Helixmith Co., Ltd.
Sun Young Kim, CEO
21, Magokjungang 8-ro 7-gil, Gangseo-gu, Seoul, South Korea

2.2 Principal Investigator and Other Clinical Study Participants

Principal investigator: Professor Byung Ok Choi
Department of Neurology
Samsung Medical Center
81, Irwon-ro, Gangnam-gu, Seoul, South Korea

See "Appendix 1. Information of Sponsor, Name and Title of Principal Investigator" for other clinical study participants.

2.3 Contract Research Organization

Dt&SanoMedics Co., Ltd.
CEO: Chae Gyu Park
15F, Daegong Building, 126, Teheran-ro, Gangnam-gu, Seoul, South Korea

3 Introduction

3.1 Incidence and Prevalence of Charcot-Marie-Tooth Disease

Hereditary neuropathy includes a variety of peripheral neuropathies that are clinically and genetically different. Charcot-Marie-Tooth disease (hereinafter CMT), also known as hereditary motor and sensory neuropathy (HMSN), is the most common disease of these hereditary neuropathies. The prevalence of CMT is approximately 1 person in every 2,500.[1]

It is known that there are approximately 2.9 million patients worldwide, and the number of patients in South Korea is estimated to be approximately 20,000. CMT is divided into several subtypes, and the most common is CMT subtype 1A (hereinafter CMT1A), the target disease of this clinical study, which accounts for 40% of all CMT. There are approximately 9,000 patients with CMT1A in South Korea, and 1.4 million CMT1A patients worldwide. CMT1A is caused by the duplication of the PMP22 gene.[2]

3.2 Pathophysiology of Charcot-Marie-Tooth Disease

CMT is a hereditary peripheral neuropathy, which is a disease caused by damage to motor and sensory nerves by gene mutations. Various clinical patterns are shown depending on the causative gene, and clinical symptoms such as amyotrophy in the upper and lower limbs, foot deformity, sensory loss, blindness, hearing loss, and areflexia are commonly observed, which show a pattern of gradually progressing over time.[3]

The peripheral nervous system (PNS) extends out from the central nerves (brain, spinal cord) and is distributed to each part of the body. It serves the role of transmitting external stimuli to the central nervous system and delivering commands of the central nervous system to muscles or each organ. The peripheral nervous system is generally divided into the somatic nervous system and the autonomic nervous system. The somatic nervous system is further divided into motor and sensory nerves. Motor nerves are efferent, and they induce muscle contraction by transmitting excitation from the center to the periphery (skeletal muscles). Sensory nerves are afferent, and they induce sensation by transmitting signals from the periphery (sensory organs) to the center. Peripheral nerves that emanate from the central nerves gradually divide and spread throughout the body to reach the peripheral organs, which are divided into the cranial nerves (12 pairs) and the spinal nerves (31 pairs) depending on where they emanate from.

Each axon within the peripheral nerves exists inside a type of tube made of myelin sheaths produced by special neuroglia called Schwann cells. An axon and myelin sheaths combined are called a nerve fiber. A number of nerve fibers form a bundle to constitute one nerve, but the number of nerve fibers varies. Approximately 1/3 of all nerve fibers are wrapped quite well. In the growth phase, Schwann cells wrap around an axon in several layers. This results in the formation of a myelin sheath made of a mixture of lipids and proteins in between the axon

and the Schwann cells. As such, a cross-section of a nerve fiber resembles an electrical wire surrounded by a thick insulation layer. A nerve fiber insulated in this way is called a myelinated or medullated fiber. The nerve conduction velocity of a myelinated nerve fiber is much faster than that of an unmyelinated nerve fiber.

CMT can be largely classified into two types based on nerve conduction studies and histological features, and these are CMT1 characterized by demyelination and CMT2 characterized by damages to axon cells. Recently, more detailed classifications have become possible as more studies on the causative gene have become active due to advances in molecular genetics technology. With over 90 genetic mutations discovered to date, they have been a great help to pathophysiological studies as well as in the classification of complex clinical types and genotypes.[4]

CMT can be divided into several subtypes based on the causative gene, and the most common and typical subtype is CMT1A, the target disease of this clinical study. CMT1A develops due to the duplication of chromosome 17p11.2-p22 that contains peripheral myelin protein 22 (PMP22), and it is an autosomal dominant inheritance.[5] In electrophysiology tests, CMT1A is characterized by a uniform decrease in nerve conduction velocity. This is a characteristic of hereditary demyelinating neuropathy that contrasts with the conduction block and temporal dispersion shown in inflammatory demyelinating neuropathy.[6]

The onset age of patients with CMT1A is usually before 10 years of age, and the symptoms start in distal regions of limbs and progress to the proximal regions. As the disease progresses after typically manifesting first in the lower limbs, its symptoms are also shown in the upper limbs. Sensory loss, weakening of muscle strength, amyotrophy, etc., start to manifest in both feet as well as in the leg below the knees, and after several years, the symptoms also manifest in the hands and arms.[7]

Fats accumulate in the atrophied muscle tissues, and the fatty infiltration level within the muscles can be identified using MRI imaging. At this time, the fat content value (%) shall be measured and evaluated at one level for each muscle. [8]

The pathogenesis of CMT1A is known to be due to an increased quantity of mRNA caused by the duplication of the PMP22 gene, which in turn produces a structurally unstable myelin sheath.[9] It has been verified that the symptoms shown in mutated rats whose duplication of the PMP22 gene had been artificially induced were similar to those shown in humans.[10] [11] Demyelination of a nerve fiber occurs due to damages in a structurally unstable myelin sheath, and the nerve conduction velocity decreases because of this.

Numerous nonmyelinating or dysmyelinating Schwann cells are produced in CMT1A due to abnormal differentiation of immature Schwann cells, and thus cause a demyelinating neuropathy. A recent study has shown that the serum concentrations of p75 neurotrophin receptor (p75) and neural cell adhesion molecule 1 (NCAM) appear different depending on the

pathologic differences of Schwann cells in various peripheral neuropathies. Both NCAM and p75 are exosome proteins that are extracellularly secreted by immature Schwann cells. [12]

p62/seqestosome-1 (p62) is an autophagy receptor, which is essential in removing intracellular organelle or denatured protein using autophagy.[13] Functional change of inctracellular autophage occurs in various ways in many cellular environments. Schwann cells, which make peripheral nerve myelin, accumulate p62 due to the suppression of autophagy in a hereditary demyelinating disease environment.[14] Not only is the p62 level significantly higher in patients with inflammatory peripheral demyelination than in control, but a greater amount of serum p62 is observed in patients with hereditary peripheral demyelation disease (CMT1A) than in patients with inflammatory demyelination. In addition, the amount of p62 in CMT1A patient's serum is considered to be a very important serum indicator for CMT1A severity, as it is positively correlated with the duration of the disease and the decrease in motor neuron function.

In a separate retrospective biomarker study, we aim to evaluate the correlation between quantitative change of serum p62, p75 and NCAM and symptom changes in CMT1A patient group.

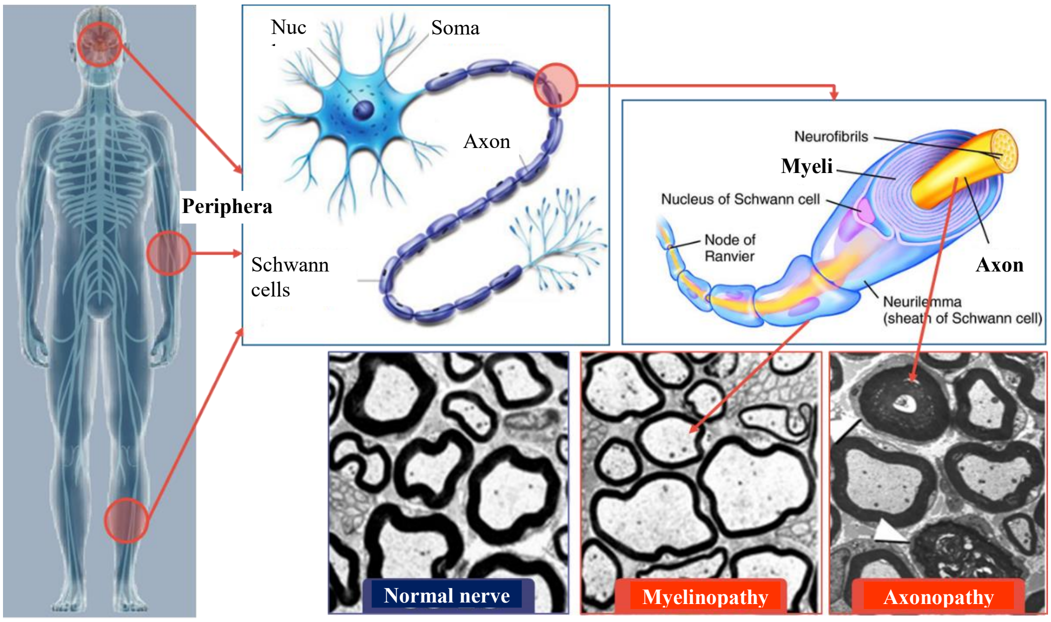

Figure 2 Pathophysiology of CMT

3.3 Latest Treatment Methods for Charcot-Marie-Tooth Disease

There are no therapeutic agents for CMT to date. Attempts were made to develop therapeutic agents, but efficacy could not be demonstrated in clinical studies. Most of the attempted developments for therapeutic agents targeted alleviating the symptoms of CMT1A by regulating the expression of PMP22.[15]

A study that administered Onapristone, a progesterone antagonist, showed potential in animal experiments, but meaningful results were not achieved in clinical studies. When progesterone is administered daily in CMT1A rats, the PMP22 concentration in blood increases in the sciatic nerve, which results in demyelinating pathologic correlation. In contrast, when the progesterone receptor antagonist, Onapristone, is administered, it was shown that the CMT phenotype of transgenic rats improved without side effects as the overexpression of the PMP22 mRNA quantity is reduced.[16]

The same was true in an ascorbic acid study. Ascorbic acid has been demonstrated to be an essential substance for the formation of myelin sheaths in the peripheral nervous system through experiments that were cultured Schwann cells along with dorsal root ganglion cells. When ascorbic acid was administered in CMT1A transgenic rats based on this rationale, re-formation of myelin sheaths and improvement of the CMT phenotype were observed, and at the same time, it was verified that the overexpression of the PMP22 mRNA quantity was inhibited to the extent required for improvement of symptoms.[17] However, meaningful results still could not be obtained in a clinical study.[18]

A curcumin study also showed efficacy in animal experiments. Apoptosis occurs when myelin protein zero (MPZ) mutation protein, which has been identified as the cause in 10% of CMT1B patients, accumulates in the endoplasmic reticulum (ER), but this can be prevented with a pretreatment of curcumin. Curcumin, a component of turmeric, serves as a sarcoplasmic/ER Ca^{2+} APTase inhibitor that suppresses what remains in the endoplasmic reticulum. When curcumin is orally administered, the phenotype of trembler J (TrJ) mouse, which has spontaneously occurring demyelination, is partially alleviated.[19]

In addition, a study had been conducted on whether curcumin can be a candidate drug for the CMT subtype caused by the accumulation of misfolded protein in the endoplasmic reticulum by orally administering curcumin in an MPZ-mutation-gene knock-in CMT1B mouse model.[20] However, this study also did not lead to a meaningful clinical study.

There have also been experiments that evaluated the effects of neurotrophin-3 (NT-3). NT-3 is expressed in Schwann cells that form the myelin sheath in peripheral nerves, and it promotes nerve regeneration. NT-3 promotes the growth of Schwann cells in nerve endings in CMT1A and creates a favorable environment for nerve regeneration, as well as synergizing with insulin-like growth factor (IGF) and platelet-derived growth factor-BB (PDGF-BB). Schwann cells which had mutations occurring in a xenograft experiment responded to NT-3, and axonal regeneration as well as the myelination process showed meaningful improvements. In a study that evaluated the effects of NT-3, axonal regeneration was promoted in the animal model, and sensory symptoms improved in the CMT1A patient group with an increase in myelinated nerve fibers. Since this was a small-scale study with four subjects each in the patient and control groups, follow-up studies are required.[21]

The PXT3003 study is the most recent study to date. PXT3003 is a combination of three drugs,

namely baclofen, naltrexone, and D-sorbitol. Baclofen is a $GABA_B$ receptor-specific agonist, and it reduces the expression of PMP22 by lowering the concentration of intracellular cAMP by decreasing the activity of adenylate cyclase. Naltrexone is an opioid receptor antagonist, and at nontoxic low doses, it lowers the concentration of intracellular cAMP by strengthening cellular signals by binding to inhibitory G alpha protein subunits. D-sorbitol is a natural metabolite, and it serves an important role in energy production/storage. As a combination of these drugs, PXT3003 is involved in inhibiting the onset of CMT1A, and this is based on the hypothesis that it improves myelination and nerve functions by reducing the toxic actions due to the overexpression of the PMP22 gene. In an animal experiment that orally administered PXT3003 in a CMT1A transgenic rat model, PMP22 transcript was downregulated in the sciatic nerve, and improvements were shown in motor functions and sensory functions (heat sensitivity) as well as in histological and electrophysiological test results. Positive results were also shown in a subsequent clinical study. The results of conducting a phase 2 clinical study in 80 subjects with CMT1A for 12 months showed improvements of 8%, 12.1%, and 20.1%, respectively, in CMTNS, ONLS, and sensory nerve conduction velocity in the high-dose group among four groups (a placebo group and three study drug groups at different doses). A phase 3 clinical study was conducted in 323 subjects for 15 months, and it was shown that the results for ONLS and the 10-meter walk test were improved.[22] Although positive results were obtained in the clinical study, the mechanism by which synergistic effects are shown when these three drugs are used in combination is yet to be identified, and it remains as a task to be undertaken in the future.[23]

3.4 Limitations of Existing Therapies and Necessity for New Therapies

Despite the fact that CMT is a disease that persists throughout a patient's lifetime with worsening symptoms, no therapeutic drugs have been developed to date that can improve outcomes in CMT patients. Since there are no aggressive treatment methods, patients undergo only conservative treatments.[24] As time passes, walking gradually becomes more difficult and living a normal daily life also becomes difficult due to worsening pain.[25] The only available option is either wearing an assistive device for walking,[26] or avoiding aggravating factors that accelerate the speed of disease progression.

Not only are various types of drugs included in the aggravating factors of CMT, but routine events that can be experienced by anyone including stress such as trauma and surgery,[27] as well as remaining in a stationary position for an extended duration are also included. Numerous types of anticancer drugs including vincristine, antibiotics, antiarrhythmic drugs, gout medications, antiviral agents, alcohol dependency medications, antirheumatic drugs, anesthetic gases, fatty acid synthase inhibitors, vitamin B6, immune-modulating agents, and the medicine for Sleeping Sickness can aggravate CMT, and thus, CMT patients may find it difficult to choose

medications when they contract other diseases.[28] Caution is necessary since endocrine diseases such as diabetes are also included in the aggravating factors.[29]

Patients suffering from the disease experience more difficulties since many elements such as these that cannot be avoided by their carefulness or effort alone are included in the aggravating factors.

Adequate exercise, physical therapy, occupational therapy, etc., are helpful in preserving their ability to perform daily activities, but special caution is necessary since excessive exercise actually aggravates the disease.[26]

3.5 Treatment of Charcot-Marie-Tooth Disease Using Hepatocyte Growth Factor (HGF)

The hepatocyte growth factor (HGF) is a secretory protein derived from mesenchyme, and c-Met is known as its unique receptor. The c-Met receptor activated by HGF shows various functions. HGF is known as a strong angiogenic growth factor and anti-apoptosis factor, and promotes the growth of vascular endothelial cells and the migration of vascular smooth muscle cells.[30][31][32][33] It is known that HGF/c-Met pathway acts dose-dependently to stimulate the synthesis of DNA, RNA, and protein in the vascular endothelial cells, and helps in angiogenesis by increasing the expression of various secretory proteins including the vascular endothelial growth factor (VEGF). Furthermore, it is known that HGF contributes to tissue regeneration with its anti-inflammatory and antifibrotic activities.[34][35]

HGF has been thought of as an angiogenic factor, but it has been recently identified as serving the role of a neurotrophic factor.[36][37][38][39][40][41][42] Moreover, it has been reported that it can contribute to muscle tissue regeneration by targeting on muscles.[43][44] Considering the pathologic mechanism of CMT, the potential of the biological mechanism of HGF to show therapeutic effects is quite high for the following reasons:

a) Targeting on peripheral nerve tissues

   According to the results of a recent study, the expression/activity of HGF and c-Met receptor increases significantly when damages to peripheral nerves occur. If the activity of c-Met is impeded in mice after peripheral nerve damage, the spontaneous regeneration process of nerve tissue is greatly hindered, and it can be observed that the conditions of myelin sheath tissue and axon are aggravated more in particular. This suggests that HGF/c-Met may be involved in the regeneration process of peripheral nerves.

   To discover more detailed mechanism, the role of HGF/c-Met in each cell that constitutes peripheral nerve tissues has been studied.

   First, Schwann cells are important cells that form the myelin sheath that surround peripheral nerves, and they serve a crucial role in maintaining the functions of peripheral nerves.[45] The c-Met receptor is present in a Schwann cell, and if HGF is

bound to this, the Schwann cell changes to be able to repair the damaged nerve. The activity of key transcription factors increases in this process, and improved expression of factors related to neuroprotection, such as the glial cell-derived neurotrophic factor (GDNF), was identified. Through this, it could be seen that HGF contributes to the reconstruction process of the myelin sheath by targeting on the Schwann cells.

Next, the effects on peripheral nerves were investigated. The c-Met receptor is also present in peripheral nerves, and it was verified that axonal growth is promoted significantly when HGF is bound to this. Not only did the activity of key transcription factors increase in this process as in Schwann cells, but the mitochondrial activity also increased, which showed that contribution is made to energy metabolism.

b) Targeting on muscle tissues

One of the major symptoms of CMT disease is muscle atrophy. Atrophy occurs in the muscles if the nerve signals transmitted to muscles are reduced due to abnormalities in nerve functions, and this in turn leads to decreased muscle function, but HGF and c-Met receptor can act toward alleviating muscle atrophy under these circumstances. The expression/activity of HGF and c-Met receptor increases when amyotrophy occurs, and the increased HGF/c-Met contributes to the alleviation of amyotrophy. In particular, it has been verified that HGF increases microRNA called miR-206 in myocytes, and thereby inhibits the expression of key amyotrophy genes. In addition, HGF induces regeneration of muscle fibers by having an impact also on the muscle satellite cells, which are stem cells present in muscles. Finally, HGF can regulate the inflammatory response shown after muscle damage, and it can show anti-inflammatory activity by reducing the secretion of inflammatory cytokines in particular.

c) Pain reduction effects

HGF can reduce pain by targeting on the peripheral nerves. In neuropathic pain-induced animal models, pain-inducing factors such as CSF1, ATF3, and calcium channel subunit $\alpha 2\delta 1$ are expressed in high levels in the spinal nerve dorsal root ganglia (DRG), and it has been verified that HGF inhibits the expression of these pain factors. Furthermore, it has been verified that HGF serves the role of inhibiting division and activation of microglia and astrocytes that are involved in neuroinflammatory response. Neuroinflammatory response mainly causes neuropathic pain. Thus, not only has the mechanism of HGF in reducing neuropathic pain been revealed in terms of molecular biology and histopathology, but it has also been revealed that the Engensis (VM202) intramuscular injection can even remodel the central nervous system circuit for neuropathic pain.[46]

d) Other actions

The neuroprotective actions of HGF have already been revealed in a study on an ALS animal model. W. Sun et al. (2002) have discovered that HGF which is continuously

produced locally in the nerve tissues of SODG93A mice, the transgenic amyotrophic lateral sclerosis (ALS) experiment model, alleviates the symptoms of ALS by activating a direct neurotrophic action on the motor nerves and having an indirect action on the glial cells.[47] In other words, the they have found that HGF performs neuroprotective actions.

The motor function improved in mice whose HGF was continuously produced, and compared with mice that did not produce it, their paresis started later and their survival was longer. These results suggest that HGF can help maintain the function of motoneurons. Thus, this shows that HGF has the potential to contribute to the improvement of motoneuron function even under CMT.[48][49][50]

When some axon cells die, the surviving nerve fibers create a network to again dominate the motor unit (muscle group) whose network had been lost due to the dead cells. As a result, a larger motor unit is created. HGF slows the progression of disease by promoting the nerve reconnection process. HGF promotes neurogenesis, angiogenesis, and synapse formation, and blocks the occurrence of fibrosis at ischemic sites.[51] In addition, HGF also has a defensive effect against excitotoxic damage. It is known to weaken axonal degeneration as excitotoxicity is reduced by regulating the expression of scaffolding protein of the NMDA receptor, and this suggests that HGF can impede neuronal degeneration under the pathologic conditions of CMT.[52]

Finally, HGF prevents cell death in motoneurons, and stops cascading damage of neighboring cells by directly inhibiting caspase signals.[52][53] Since caspase remains active in each neuron for an extended length of time (can be from several weeks to several months), cellular dysfunction can be reduced and cell death can be delayed if caspase is inhibited under these conditions.[54]

Figure 3 shows the mechanism of HGF on motoneurons. HGF binds to c-Met on the cell surface, and induces the autophosphorylation response of intracellular tyrosine residues of c-Met.

Subsequently, HGF inhibits the activation of caspase-1, and effectively prevents the caspase cascading reactions that follow, such as caspase-3, -7, and -9, by inducing x-linked inhibitor of apoptosis protein (XIAP). Thus, at least a part of the neurotrophic action of HGF in motoneurons is promoted by preventing caspase-mediated cell death signals. Considering this series of actions, if HGF is applied to CMT, there is sufficient potential for HGF to show therapeutic effects by affecting degenerating neurons.

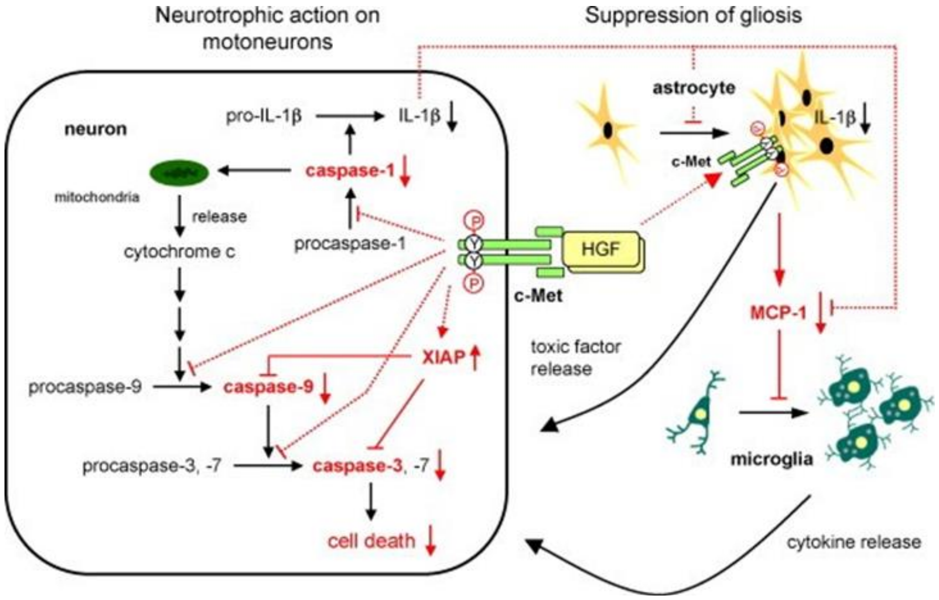

Figure 3 Molecular mechanism of neuroprotective effect of HGF

From: Kadoyama, K., Funakoshi. H, et al. (2007) [45]

3.6 Engensis; VM202

Hepatocyte growth factor is known to bind to the c-Met receptor on the cell membrane having tyrosine kinase activity and promoting various cell division, migration, and angiogenesis, as well as inhibiting apoptosis.[55]

Two difficulties must be overcome to deliver a target quantity of exogenous HGF. The first difficulty is that HGF is unstable in the bloodstream, and the second is that it is rapidly metabolized in the liver; the in vivo half-life of HGF is 15 minutes.[56][57]

Thus, a way to increase the available HGF in neurons is to develop a gene delivery strategy that allows continuous expression of HGF protein in vivo. Although plasmid DNA has the lowest efficiency among the gene transfer systems that have recently used, using local targeted delivery is a highly attractive choice (especially in skeletal muscles) due to the facts that its persistence in vivo is limited and that gene insertion is unnecessary.

Engensis (VM202) is the drug developed to achieve this very objective, and it is the study drug that will be used in this clinical study. Engensis (VM202) is a DNA plasmid containing a new recombinant HGF gene (HGF-X7) that has been made to simultaneously express two isoforms (HGF_{723}, HGF_{728}) of the hepatocyte growth factor (HGF).

The main feature of HGF-X7 is that it has been designed to efficiently express two isoforms of HGF simultaneously in the same way as in the human body by inserting a portion of the intron base sequence into a specific site of HGF cDNA. In addition, since there are no changes in the coding regions of HGF, the HGF proteins expressed by Engensis (VM202) are identical to the proteins produced in vivo.

Engensis (VM202) is efficiently delivered to even those cells that have completed division such as skeletal muscles or neurons. The HGF protein is expressed locally only at the administration site, and other organs are not affected. These facts have been demonstrated in the previous study. Thus, effecting neurotrophic factor actions by delivering the HGF gene to the target muscle tissue by using the plasmid and then expressing proteins is a highly attractive and safe treatment method. Moreover, since the Engensis (VM202) plasmid remaining in vivo after the intramuscular injection exists outside the chromosome, the probability of it being inserted into the chromosome of the patient is almost negligible.[28-30] These local effects of plasmids are already well known from previous studies that injected plasmid DNA.[58][59]

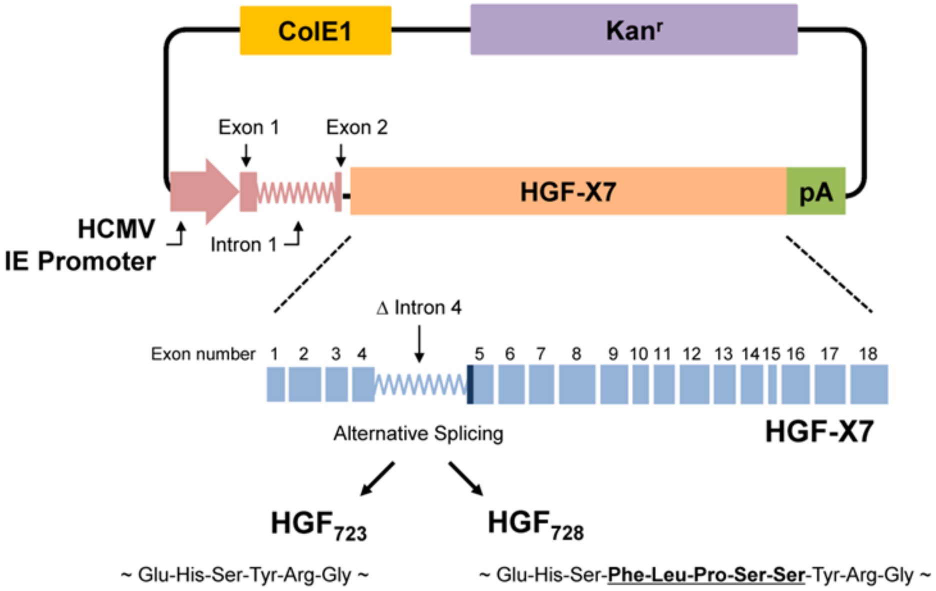

Figure 4 Structure of Engensis (VM202)

Since pCK is being used as the transporter of HGF gene, Engensis (VM202) is also called pCK-HGFX7. The plasmid of Engensis (VM202) is made up of 7,377 base pairs, and in addition to the HGF coding sequence in the pCK framework, it is composed of HCMV enhancer/promoter, exon1 of HCMV IE gene, intron A, 5'-noncoding sequences of exon2, polyA of bovine growth hormone (BGH), ColE1, kanamycin resistance gene, etc.

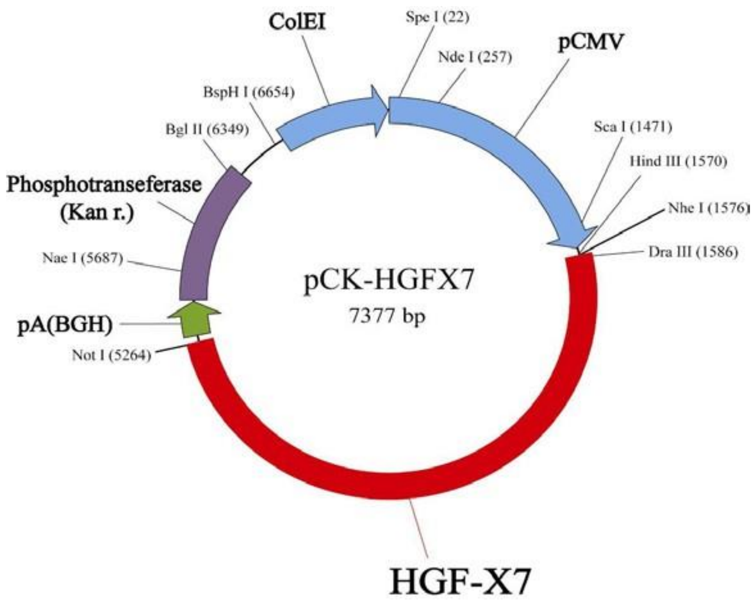

Figure 5 Engensis (VM202) plasmid map

Table 1 Plasmid component factors

1 Gene	2	Locatio n	3 Functi on
4 HCMV<br>5 enhancer/promot er, IE1 exon1, intron A, 5′-noncoding sequences of exon2	6	1 ~ 1568 bp	7 It is a promoter derived from the major immediately early gene of human cytomegalovirus, and it regulates the expression of the lower target gene (HGF-X7).
8 HGF-X7	9 bp	1584 ~ 5262	10 It is a fusion of the cDNA of the human hepatocyte growth factor and genomic DNA, and it induces angiogenesis by promoting the growth of vascular endothelial cells and the migration of vascular smooth muscle cells.
11 polyA (BGH)	12 bp	5300 ~ 5514	13 It is a polyadenine sequence of the bovine growth hormone gene, and it is used to terminate the transcription of the upper HGF-X7.
14 Phosphotransferase<br>15 (Kanr.)	16 bp	5523 ~ 6317	17 It is a gene resistant to kanamycin, and<br>18 it is used to selectively culture the E. coli with this plasmid DNA.
19 ColE1	20	6703 – 7376 bp	21 It is a plasmid DNA replication sequence, and it regulates plasmid replication in E. coli.

3.7 Nonclinical Study Data

(1) Toxicity study

In nonclinical studies, the safety of Engensis (VM202) was assessed with an intramuscular single-dose toxicity study in rats as well as an intravenous single-dose toxicity study. Additionally, safety was also assessed in intermittent intramuscular repeat-dose toxicity studies (weekly or monthly) in rabbits and rats used.

The possibility of genomic integration at the injection site or the possibility of distribution and persistence of Engensis (VM202) in reproductive tissues were evaluated in experiments conducted in rats. In the intramuscular administration of mice, the possibility of Engensis (VM202) to induce humoral immune response was evaluated through an experiment that either administered or did not administer an adjuvant concomitantly.

In conclusion, the results of various toxicity studies showed that Engensis (VM202) was well-tolerated in all studies, and the only toxicity was mild and transient injection site irritation. Evidence of systemic toxicity was not shown at all in any of the studies, and there was also no evidence of human HGF detected in the serum of rats or rabbits after intramuscular injections. [lower limit of quantitation (LLOQ) = 125 pg/mL]. Genomic integration, germ cell transmission, immunostimulatory effects, etc., were not shown at all when Engensis (VM202) was administered intramuscularly.

Table 3. Engensis Toxicology Studies

Study Design<br>Animal Model	Key Findings	VM202 Dose (mg/kg)<br>Delivery Route	Injection(s)<br>Assessment Time(s)
Dose-Related Toxicity			
**Single Dose**<br>Normal rat	No systemic toxicity<br>Mild, transient injection site irritation on Days 2, 7<br>No observed AE level > 1.2 mg/kg	0, 1, 2<br>Intramuscular	1<br>Day 2, 7, or 28
**Repeat Dose**<br>Normal rabbit	No local or systemic toxicity<br>Mild, transient injection site irritation on Days 2, 7<br>No observed AE level > 1.2 mg/kg	0, 0.3, 0.6, 1.2<br>Intramuscular	1 per week x 5<br>8 weeks
**Transcatheter**<br>Chronic ischemia vs normal pig	No difference in safety between groups<br>Needle-related trauma at Day 3 in all groups	0, 1, 4 (total doses)<br>Intramyocardial	8, 16<br>Day 3, 30, or 60
**Single Dose**<br>Normal rat	No toxicity<br>No observed AE level > 6.84 mg/kg	0, 0.068, 0.684, 6.84<br>Intramuscular	1<br>Day 14
**Single Dose**<br>Normal rat	No systemic toxicity<br>Mild decreased RBC, Hb, Hct at 0.684, 6.84 mg/kg (males)<br>No observed AE level > 6.84 mg/kg	0, 0.068, 0.684, 6.84<br>Intravenous	1<br>Day 14
**Repeat Dose**<br>Normal rat	No systemic toxicity<br>No observed AE level > 3.42 mg/kg	0.034, 0.342, 3.42<br>Intramuscular	1 per week x 4<br>Day 14
Genomic Integration			
**Genomic Integration and Reproductive Tissue Distribution**<br>Normal rat	No systemic toxicity<br>No VM202 genomic integration<br>No distribution to or expression in reproductive tissues	3.42<br>Intramuscular	2:1 at 0, 4 weeks<br>Day 7 or 42
Immunotoxicity			
**Autoimmune**<br>BALB/c mouse	No systemic toxicity<br>No antibody secreting cells in spleen<br>No serum antibodies detected	0, 0.57, 0.57 + FA; or ovalbumin + FA or ovalbumin alone<br>Intramuscular	2:1 at 0, 4 weeks<br>Day 14
**Reproductive, Developmental Toxicity**<br>Pregnant rabbit	No maternal systemic toxicity<br>No effects on mating, fertility, ovarian, or uterine parameters or natural delivery<br>No Engensis-related effects on external, soft tissue or skeletal abnormalities of fetus<br>No Engensis-related effects on external, soft tissue<br>No teratogenesis	0, 0.6, 1.2<br>Intramuscular	7:1 at Days 14 and 7 before mating and on gestation<br>Days 0, 7, 1 4, 21 and 28

AE, adverse effects; FA, Freund's complete adjuvant; Hb, hemoglobin; Hct, hematocrit; RBC, red blood cells

Figure 6 Toxicity study data (see IB)

(2) Efficacy study

After administering Engensis, the following efficacy assessments were performed.

- Nerve regeneration effects: After administering Engensis (VM202), the reconstruction of myelin sheath by Schwann cells was promoted, and regeneration of the degenerated axon was promoted as well.
- Muscle regeneration effects: After administering Engensis (VM202), nerve damage-induced amyotrophy was improved. The muscle regeneration process after muscle

damage was promoted.

- Pain reduction effects: After administering Engensis (VM202), neuropathic pain was reduced.
- Cardiovascular function improvement effects: After administering Engensis (VM202), blood flow was improved as angiogenesis was promoted, and cardiac function was improved as well.

Thus, these study results for efficacy and safety from nonclinical studies provide a sufficient rationale for conducting an Engensis (VM202) clinical study in CMT patients.

3.8 Results of Previously Conducted Clinical Studies

Starting with a domestic phase 1 study on angina pectoris in 2007, six clinical studies in total have been conducted on the investigational product (Engensis [VM202]) in South Korea and the US as of 2020 (see Table 2), and the following is a summary of the information on each clinical study.

Table 2 Summary of Clinical Studies Conducted on Engensis (VM202)

Indication	Phase	Number of Subjects	Country	Start Date*	End Date**
Critical limb ischemia	Phase 1	12	USA	March 2007	October 2009
Critical limb ischemia	Phase 2	50	USA, South Korea	June 2010	July 2013
Diabetic peripheral neuropathy	Phase 1/2	12	USA	May 2010	February 2012
Diabetic peripheral neuropathy	Phase 2	103	USA, South Korea	September 2012	March 2014
Amyotrophic lateral sclerosis	Phase 1/2	18	USA	February 2014	September 2015
Angina pectoris	Phase 1	9	South Korea	January 200[illegible]	February 2010

*. Start date: Enrollment date of first subject in the clinical study/ **. End date: Last visit date of last subject in the clinical study

3.8.1 Phase 1 Clinical Study in Patients with Critical Limb Ischemia

A phase 1 clinical study (ascending dose, single-center) of Engensis (VM202) in subjects with critical limb ischemia (CLI) was approved by the US FDA in November 2006 (approval no.: BB IND 13,158), and it was conducted at the Minneapolis Heart Institute from March

2007 to October 2008.[60] (clinicaltrials.gov identifier: NCT00696124)
The subjects of the clinical study were patients with critical limb ischemia who had been diagnosed as Rutherford class 4 or 5, and who were in a condition that was untreatable with existing therapies (drugs, surgeries, procedures). Safety, tolerability, and preliminary efficacy were assessed by administering Engensis (VM202) to the ischemic site (calf muscle) of the subjects. The clinical study was conducted with 12 subjects in total with three subjects assigned to each of the four cohorts. A different dose of Engensis (VM202) was administered to each cohort (cohort 1: 2 mg; cohort 2: 4 mg; cohort 3: 8 mg; cohort 4: 16 mg). The drug was administered twice in total at an interval of two weeks, and follow-up was performed for 12 months. Evaluation of the main assessment criterion (safety) was performed on all subjects who had been administered the drug, and the adverse events (including serious adverse events and adverse events that can lead to discontinuation of administration) that occurred during the 12-month follow-up period were described according to their severity and relation to the investigational product or the administration procedure.
In addition, the safety endpoints were specified by using a descriptive statistical method (number, mean, median, standard deviation, minimum, and maximum). For the 12-month follow-up period after administering Engensis (VM202), the preliminary efficacy was assessed with the changes in transcutaneous partial pressure of oxygen (TcPO2), the changes in pain severity measured using a visual analogue scale (VAS), and the changes in ulcer size.

Results of safety assessment: The tolerated dose of Engensis (VM202) administered intramuscularly in CLI subjects was 16 mg, and 41 cases of adverse events occurred in a total of 12 subjects. One of these adverse events was bruising of the injection site, which was caused by the administration procedure. The remaining 40 cases of adverse events were determined as not related to Engensis (VM202). A total of 10 cases of severe adverse events occurred in six subjects, and there were none related to Engensis (VM202) among them. There were no subjects who died during the 12-month follow-up period, and on subject underwent a lower limb amputation.

Results of preliminary efficacy assessment: The ankle-brachial index (ABI) of 0.35 prior to administration increased to 0.52 at the follow-up 12 months after administration ($p = 0.005$). The toe-brachial index (TBI) of 0.15 prior to administration also increased to 0.24 at the follow-up 12 months after administration ($p = 0.01$). TcPO2 levels also showed a tendency of improvement. In addition, the results of performing pain assessment with a visual analogue scale (VAS) six months after administration of Engensis (VM202) showed effects of pain improvement in 9 out of 11 subjects (before administration vs. 6 months

after administration: 58 mm vs. 16 mm, p = 0.03).
In the phase 1 clinical study on CLI subjects, it was verified that the tolerated dose of Engensis (VM202) was 16 mg, and adverse events that were directly related to Engensis (VM202) were not observed. Although one subject underwent lower limb amputation due to osteomyelitis, it was determined as not related to Engensis (VM202) since it was due to the worsening of an underlying disease. These results showed that the method of administering Engensis (VM202) into the ischemic muscle is safe for CLI patients, and also showed the potential of the drug to fundamentally improve the pain following ischemia by improving blood flow and blood pressure through the induction of angiogenesis in the injected muscle.

3.8.2 Phase 2 Clinical Study on Critical Limb Ischemia

A phase 2 clinical study (multicenter, placebo-controlled, randomized, double-blind) of Engensis (VM202) in subjects with critical limb ischemia was approved by the US FDA and the MFDS of South Korea in July 2009 and April 2010 (approval no.: US FDA-BB IND 13,158; South Korea MFDS No. 15224), and it was conducted at a total of 13 institutions in the US and South Korea until March 201[61] (clinicaltrials.gov identifier: NCT01064440)
The purpose of the clinical study was to assess the efficacy and safety of Engensis (VM202) administered in the ischemic lower limb (calf muscle) of poor-option CLI subjects (Rutherford class 4 or 5). The study group was composed of two Engensis (VM202) administration groups (low dose: 8 mg; high dose: 16 mg) and a placebo control group (normal saline). The study drug was administered only in one calf muscle, and it was administered four times in total at an interval of two weeks. Afterward, follow-up was performed for 12 months. A total of 50 subjects were randomized with 20 subjects in the Engensis (VM202) low-dose group, 20 subjects in the high-dose group, and 10 subjects in the placebo control group.
Safety assessment was performed on all subjects who had been administered the study drug (Engensis [VM202] or placebo), and the adverse events (including serious adverse events and adverse events that can lead to discontinuation of administration) that occurred during the 12-month follow-up period were described according to their severity and relation to the investigational product or the administration procedure. In addition, the safety endpoints were specified by using a descriptive statistical method (number, mean, median, standard deviation, minimum, and maximum). Efficacy assessment was compared the differences in the therapeutic effects between the administration groups and the placebo control group by evaluating the changes in pain severity and ulcer size through VAS measurements prior to study drug administration and nine months after administration, as well as measurements of ulcer size (changes in the complete healing

level of ulcer, ulcer size, and number) and transcutaneous partial pressure of oxygen.

Results of safety assessment: Among the adverse drug reactions, those that were directly related to Engensis (VM202) were not observed. A total of 67 cases of serious adverse events occurred in 26 subjects, and there were no significant differences between the administration groups. Most of the serious adverse events were due to the worsening of underlying diseases, and there were none that were directly related to the study drug. One case of deep vein thrombosis was determined as possibly related to the administration procedure of the study drug. There were no subjects diagnosed with cancer or proliferative retinopathy during the follow-up period. There were two subjects that died. One subject belonged to the control group (death due to MRSA infection), and the other subject died 294 days after administering the low dose of Engensis (VM202). The cause of death was verified as worsening of an underlying disease (end-stage renal failure). The results of measuring the residual quantity of Engensis (VM202) in blood for all subjects showed that Engensis (VM202) exists as a quantity at or below the lower limit of quantitation within one week after administration, and that the HGF protein quantity in blood is maintained at a stable level of 0.7 to 8.7 ng/ml without any special changes during the follow-up period after administration.

Results of efficacy assessment: It was verified that the complete healing effect on ulcers was higher in the low-dose group (51.9%, 14/27 ulcers, $p = 0.0514$) and the high-dose group (61.5%, 8/13 ulcers, $p = 0.0306$) compared to the control group (11.1%, 1/9 ulcers). It was also verified that the ≥ 50% diameter reduction effect on ulcers was higher in the high-dose group (9/13 ulcers, $p = 0.0115$) and the low-dose group (19/27 ulcers, $p = 0.0046$) compared to the control group (1/9 ulcers). Significant differences ($p < 0.05$) in TcPO2 were shown between the three groups at 12 months after administration of Engensis (VM202), and it was verified that TcPO2 increased at a statistically significant level ($p < 0.05$) in the high-dose group compared to the placebo control group. The results of VAS, ABI, and TBI measurements were not statistically significant between the Engensis (VM202) administration groups and the placebo administration group, but it was verified that the improvement effect in the high-dose group was more evident compared to the low-dose group and placebo group.

In conclusion, at 12 months after administering 16 mg of Engensis (VM202), it was verified that the improvement effects on the ulcer size, complete healing of ulcers, and TcPO2 were statistically significant compared to the control group. These results showed that the method of administering Engensis (VM202) into the ischemic muscle is safe for CLI patients, and also showed the potential of reduction effect on the size of ulcers through the induction of angiogenesis in the injected muscle.

3.8.3 Phase 1/2 Clinical Study on Painful Diabetic Peripheral Neuropathy

A phase 1/2 clinical study (ascending dose, multicenter) of Engensis (VM202) in subjects with painful diabetic peripheral neuropathy was approved by the US FDA in July 2009 (BB IND 13,938), and it was conducted at two institutions in total in the US.[62] (clinicaltrials.gov identifier: NCT01002235)

The purpose of the clinical study was to assess the safety, tolerability, and preliminary efficacy of Engensis (VM202) administered in one lower limb (calf muscle) of subjects with painful diabetic peripheral neuropathy.

The study group was composed of a total of three cohorts. There were 12 subjects in total after recruiting four subjects into each cohort (cohort 1: 4 mg; cohort 2: 8 mg; cohort 3: 16 mg), and the study drug was administered twice at an interval of two weeks in one calf muscle of the subjects. Afterward, follow-up was performed for 12 months. As a main assessment criterion, safety assessment was performed on all subjects who had been administered the study drug (Engensis [VM202] or placebo), and the adverse events (including serious adverse events and adverse events that can lead to discontinuation of administration) that occurred during the 12-month follow-up period were described according to their severity and relation to the investigational product or the administration procedure. In addition, the safety endpoints were specified by using a descriptive statistical method (number, mean, median, standard deviation, minimum, and maximum). Preliminary efficacy was assessed with the changes in various pain assessment indicators (VAS, SF-MPQ, BPI-DPN) during the 12-month follow-up period after the administration of Engensis (VM202).

Results of safety assessment: The tolerated dose of Engensis (VM202) administered intramuscularly in the calf muscle of subjects with diabetic peripheral neuropathy was 16 mg, and no serious adverse events or unexpected severe adverse events were observed during the follow-up period. The results of measuring the residual quantity of Engensis (VM202) in blood for all subjects during the follow-up period showed that Engensis (VM202) exists as a quantity at or below the lower limit of quantitation at 90 days after administration. In addition, the quantity of HGF protein in blood was maintained at a stable level without any special changes during the follow-up period. These results suggest that Engensis (VM202) performs effective actions while remaining locally only at the injection site.

Results of preliminary efficacy assessment: Pain reduction effects were verified in 10 (83%) out of 12 subjects (based on the final follow-up day). The mean pain reduction assessed by VAS was > 56%. The same pattern of pain reduction effect was verified by the brief

pain inventory-diabetic peripheral neuropathy (BPI-DPN), a pain assessment method different from VAS. These results show that Engensis (VM202) up to 16 mg is safe for patients with painful diabetic peripheral neuropathy, and that it can effectively reduce pain.

3.8.4 Phase 2 Clinical Study on Painful Diabetic Peripheral Neuropathy

A phase 2 clinical study (multicenter, placebo-controlled, randomized, double-blind) of Engensis (VM202) in subjects with painful diabetic peripheral neuropathy was approved by the US FDA and the MFDS of South Korea in November 2011 and September 2012 (approval no.: US FDA-BB IND 13,938; South Korea MFDS - No. 15224), and it was conducted at a total of 17 institutions in the US and South Korea from October 2012 to March 2014.[63] (clinicaltrials.gov identifier: NCT1475786)

The purpose of the clinical study was to assess the efficacy and safety of Engensis (VM202) administered in both lower limbs (calf muscle) of subjects with painful diabetic peripheral neuropathy.

The study group was composed of two Engensis (VM202) administration groups (low dose: 16 mg; high dose: 32 mg) and a placebo control group (normal saline). The study drugs were administered in the calf muscle of both legs, and the administration was performed twice in total at an interval of two weeks. The follow-up to assess the efficacy and safety of Engensis (VM202) was performed for 9 months after the administration of Engensis (VM202), and total of 103 subjects were randomized with 40 subjects in the Engensis (VM202) low-dose group, 42 subjects in the high-dose group, and 21 subjects in the placebo control group. The efficacy assessment criterion was the comparison of the difference in pain severity between the Engensis (VM202) administration groups and the placebo control group at six months after the administration of the study drug. The Daily Pain and Sleep Interference Diary was used to measure the severity of pain. Evaluations for pain assessment indicators such as BPI-DPN and pain VAS were performed to further verify the pain reduction effects. Changes in neuropathy symptoms and sensory nerves were assessed with the Michigan Neuropathy Screening Instrument (MNSI), monofilament, and skin tissue biopsy, while changes in the quality of life were assessed with the Patient's Global Impression of Change (PGIC). Safety assessment was performed on all subjects who had been administered the study drug (Engensis [VM202] or placebo), and the adverse events (including serious adverse events and adverse events that can lead to discontinuation of administration) that occurred during 9-month follow-up period were described according to their severity and relation to the study drug or the administration procedure. In addition, the safety endpoints were specified by using a descriptive statistical method (number, mean, median, standard deviation, minimum, and maximum).

Results of safety assessment: There were no deaths or serious adverse events related to the drug. Thirteen cases of serious adverse events were confirmed in 13 subjects, and 202 cases of adverse events were confirmed in 69 subjects, but there were no adverse events related to the drug among them. The results of measuring the residual quantity of Engensis (VM202) as an assessment of the pharmacodynamics of Engensis (VM202) showed that it was not detected at three months after administration, and changes in the quantity of HGF protein in serum was maintained at a stable level (1 to 2 ng/ml) during the nine-month follow-up period. There were no subjects diagnosed with cancer or proliferative retinopathy during the follow-up period.

Results of efficacy assessment: The results of evaluating the pain reduction effects and the level of sleep deprivation due to pain as an assessment of efficacy showed that pain was reduced to a significant level ($p < 0.05$) both clinically and statistically compared to the placebo control group at three months after administering Engensis (VM202) in the Engensis (VM202) low-dose group, and it was verified that this effect was maintained for up to nine months. Compared to the Engensis (VM202) high-dose group and placebo control group, it was verified that the improvement effects on the level of sleep deprivation due to pain was more evident in the Engensis (VM202) low-dose group ($p < 0.05$). Even when the severity of pain and the improvement effects on daily life interference due to pain were assessed using BPI-DPN, it was verified that the level of improvement in the Engensis (VM202) low-dose group was both clinically and statistically significant ($p < 0.05$). These results were also consistent with the VAS, MNSI, and PGIC assessment indicators. In addition, it was verified in the monofilament test of MNSI that there was a statistically significant level of sensory improvement effect in the Engensis (VM202) low-dose group compared to the control group ($p < 0.05$). The density of sensory nerves in the biopsied skin tissue did not show significant differences between the three groups.
In the various types of pain assessment indicators, the maximum analgesic effect of Engensis (VM202) was shown at 8 mg/leg rather than 16 mg/leg, and it was verified that the dose response of Engensis (VM202) was hormetic (U-shaped) rather than S-shaped. In addition, the pain improvement effects on subjects that had not taken Lyrica/Neurontin, the existing drugs for diabetic neuropathy, were more evident than those on subjects that had taken these drugs ($p < 0.01$). Furthermore, adverse events directly related to Engensis (VM202) were also not shown. These results suggest that administering Engensis (VM202) in the calf muscle of patients with diabetic neuropathy is safe, and that it can serve as a disease modifying drug that can fundamentally improve the conditions of the disease, when the monofilament test results are also considered.

3.8.5 Phase 1/2 Clinical Study on Amyotrophic Lateral Sclerosis

A phase 1/2 clinical study of Engensis (VM202) in subjects with amyotrophic lateral sclerosis (ALS) was approved by the US FDA in October 2013 (approval no.: BB IND 15,761), and it was conducted at the Northwestern Memorial Hospital from February 2014 to September 2015.[64] Engensis (VM202) was designated as an orphan drug by the US FDA in February 2015 (clinicaltrials.gov identifier: NCT02039401).

The purpose of the clinical study was to assess the safety and tolerability of Engensis (VM202) by administering it to bilateral upper and lower limbs in subjects with amyotrophic lateral sclerosis.

The study group was divided into two Engensis (VM202) administration groups. Group 1 administered Engensis (VM202) in the following order: lower limb (19 mg) / upper limb (13 mg) / lower limb (19 mg) / upper limb (13 mg). Group 2 administered the drug in the following order: upper limb (13 mg) / lower limb (19 mg) / upper limb (13 mg) / lower limb (19 mg). A total of 18 subjects were recruited and nine subjects were randomized to each group, and a total of 64 mg of Engensis (VM202) was administered four times (Day 0, Week 1, Week 2, Week 3) at one-week intervals. Safety assessment, the main assessment criterion, was performed on all subjects who had been administered Engensis (VM202), and the adverse events (including serious adverse events and adverse events that can lead to discontinuation of administration) that occurred during the nine-month follow-up period were investigated according to their severity and relation to the study drug or the administration procedure. The progression status of amyotrophic lateral sclerosis was assessed for the assessment of preliminary efficacy, and methods such as ALSFRS-R questionnaire survey, measurement of muscle circumference, forced vital capacity (FVC), and dynamometry were used.

Results of safety assessment: A total of 79 cases of adverse events occurred in 17 subjects (94.4%). Five cases of serious adverse events were reported in three subjects, but they were reported as not being directly related to the drug. All adverse events were resolved without additional treatment during the follow-up period. It was verified that there was no statistically significant difference in the incidence of adverse events between the two groups. The results of measuring the residual quantity of Engensis (VM202) as an assessment of the pharmacodynamics of Engensis (VM202) showed that it was not detected at three months after administration, and changes in the quantity of HGF protein in serum was maintained at a stable level (0.7 to 2.4 ng/ml) during the nine-month follow-up period. There were no subjects diagnosed with proliferative retinopathy during the follow-up period. One subject died of respiratory failure due to the progression of ALS during the follow-up period.

Results of efficacy assessment: The results of analyzing changes in ALSFRS-R showed that the progression slope/month was improved up to three months after the administration of Engensis (VM202). The subject group that responded to Engensis (VM202), defined as having no changes in ALSFRS-R or as having shown improvement, was 47% at month one, 50% at month two, and 24% at month three post-administration. In addition, muscle strength and muscle circumference were also stably maintained up to three months after the administration of Engensis (VM202). These results show that administering 64 mg of Engensis (VM202) is safe, and that it can stop or improve the progression of ALS for two to three months after administration.

3.8.6 Phase 1 Clinical Study on Angina Pectoris

A phase 1 clinical study (open-label, non-comparative, ascending dose, single-center) of Engensis (VM202) in subjects with angina pectoris was approved by the MFDS of South Korea in October 2006 (approval no.: No. 15224), and it was conducted at the Seoul National University Hospital from January 31, 2007 to February 5, 2010.[65] (clinicaltrials.gov identifier: NCT01422772)

Among the patients targeted for coronary artery bypass surgery, the subjects of the clinical study were patients who could not undergo vascular anastomosis due to poor vascular condition in the myocardial region with decreased perfusion and who were expected to have incomplete reperfusion in the corresponding myocardial region.

The purpose of the clinical study was to assess the safety, tolerability, and preliminary efficacy of Engensis (VM202) administered in the myocardial region expected to have incomplete reperfusion.

The study group was composed of a total of three cohorts (cohort 1: 0.5 mg; cohort 2: 1.0 mg; cohort 3: 2.0 mg), and the clinical study was conducted in a total of nine subjects by recruiting three subjects for each cohort. The adverse events (including serious adverse events and adverse events that can lead to discontinuation of administration) that occurred during the six-month follow-up period after the administration of Engensis (VM202) were assessed according to their severity and relation to the investigational product dose or the administration procedure. In addition, the safety endpoints were specified by using a descriptive statistical method (number, mean, median, standard deviation, minimum, and maximum). For the assessment of preliminary efficacy, the changes in cardiac function (global and regional LVEF by cardiac MRI, myocardial MIBI-SPECT, and echocardiography), changes in the size of viable myocardium (myocardial thickness of the site injected with Engensis [VM202] imaged with cardiac MRI, scope of late gadolinium enhancement, motor intensity of local site), and changes in the ischemic myocardial region (changes in myocardial perfusion at rest and under load imaged with myocardial MIBI-SPECT) were assessed for six months after the administration of Engensis (VM202).

Results of safety assessment: The tolerated dose of Engensis (VM202) administered in the ischemic myocardium of subjects with angina pectoris was 2 mg. A total of 112 cases of adverse events occurred in the nine subjects that participated in the clinical study, but there were no adverse events directly related to Engensis (VM202), and most were typical adverse events that were due to the worsening of underlying diseases or that occur after coronary artery bypass surgery. The quantity of HGF protein in blood was maintained at a stable level without any special changes during the follow-up period, and it was verified that antibodies for the HGF protein expressed by Engensis (VM202) were not produced.

Assessment of preliminary efficacy: The results of preliminary efficacy assessment using myocardial MIBI-SPECT, cardiac MRI, and echocardiography showed statistically significant improvement effects on the intramyocardial perfusion (before administration vs. at three or six months after administration, $p < 0.05$) of the Engensis (VM202) administration site, the myocardial thickness at end systolic phase and end diastolic phase of the left ventricle (before administration vs. at three months after administration, $p < 0.05$), and the myocardial wall motion index (before administration vs. at six months after administration, $p < 0.05$).

In the phase 1 clinical study on subjects with angina pectoris, it was verified that the tolerated dose of Engensis (VM202) was 2 mg, and adverse events that were directly related to Engensis (VM202) were not observed. These results show that the method of administering Engensis (VM202) into the ischemic myocardium is safe for patients with angina pectoris who undergo coronary artery bypass surgery, and that it improves the blood flow by inducing angiogenesis within the injected myocardium. In addition, the results also show the potential of the drug to fundamentally improve cardiac functions by recovering the thickness of ischemic myocardium through protective effects on the myocardium.

3.9 Rationale for Establishing Dose of Investigational Product

The administration dose of VM202, the investigational product of this study, has been established based on the results of nonclinical studies, as well as the administration doses and pharmacodynamic assessments in past clinical studies.

Rationale 1. Nonclinical Study Results

(1) Single-dose toxicity study

A single intramuscular dose toxicity study and a single intravenous dose toxicity study were

conducted in rats. Even when a dose of 6.840 mg/kg was administered, toxicity reactions, including death due to the drug were not shown in either males or females.

(2) Repeat-dose toxicity study

A 4-week intermittent intramuscular repeat-dose toxicity study (1.2 mg/kg/day, once a week, 5 doses in total) in rabbits, as well as a 13-week intermittent intramuscular repeat-dose toxicity study (3.420 mg/kg/day, once a month, 4 doses in total) in rats were conducted. Except for minor and transient irritations on the injection site, target organs with toxicological findings were not identified.

Table 3. Toxicity study results

Study title	Animal	Dose	NOAEL[1)]	HED[2)]
A Single Intramuscular Toxicity Study of VM202 in Rats(G04110)	Sprague-Dawley rat	0.068~6.840 mg/kg	>6.840 mg/kg	>1.09 mg/kg (65.66 mg/60 kg)
A Single Intravenous Toxicity Study of VM202 in Rats(G04113)	Sprague-Dawley rat	0.068~6.840 mg/kg	>6.840 mg/kg	>1.09 mg/kg (65.66 mg/60 kg)
28 Day Repeat Exposure Toxicity Study of VM202 in Rabbits(C3881)	New Zealand white rabbits	0.3~1.2 mg/kg (once a week, 4 weeks)	>1.2 mg/kg/day	>0.38 mg/kg (23.04 mg/60 kg)
13-Week Repeat Intramuscular Toxicity Study of VM202 in Rats(G04114)	Sprague-Dawley rat	0.034~3.420 mg/kg (once a month, 4 months)	>3.420 mg/kg	>0.55 mg/kg (32.83 g/60 kg)

1) NOAEL: no-observed-adverse-effect level, 2) HED: human equivalence dose

Rationale 2. Previously Conducted Clinical Studies

Starting with the phase 1 clinical study on angina pectoris in South Korea in 2007, as of 2020, six clinical studies in total have been conducted in South Korea and the United States (see section 3.8). The administration dose in each clinical study is as follows:

Table 4. Administration dose per visit in past clinical studies

Study title	Total dose	Dose VM202 per Visit			
Phase 1 clinical study in patients with critical limb ischemia	~16 mg	Day 0		Day 14	
		8 mg (2.0 ml x 8)		8 mg (2.0 ml x 8)	
Phase 2 clinical study in patients with critical limb ischemia	~16 mg	Day 0	Day 14	Day 28	Day 42
		4 mg (0.5 ml x 16)	4 mg (0.5 ml x 16)	4 mg (0.5 ml x 16)	4 mg (0.5 ml x 16)
Phase 1/2 clinical study in patients with diabetic peripheral neuropathy	~16 mg	Day 0		Day 14	
		8 mg (0.5 ml x 32)		8 mg (0.5 ml x 32)	
Phase 2 clinical study in patients with diabetic peripheral neuropathy	~32 mg	Day 0		Day 14	
		16 mg (0.5 ml x 64)		16 mg (0.5 ml x 64)	
Phase 1/2 clinical study in patients with amyotrophic lateral sclerosis	~64 mg	Day 0	Day 7	Day 14	Day 21
		13 mg (0.5 ml x 52)	19 mg (0.5 ml x 76)	13 mg (0.5 ml x 52)	19 mg (0.5 ml x 76)
Phase 1 clinical study in patients with angina pectoris	~2 mg	0.25 ml x 8			

Rationale 3. Pharmacodynamic Assessment of Investigational Product (VM202)

The results of measuring the residual quantity of VM202 for the pharmacodynamic assessment of the investigational product (VM202) in the phase 1/2 clinical study on patients with amyotrophic lateral sclerosis showed that the drug was not detected at 3 months post-administration of VM202 (see section 3.8.5).

Results for Rationale of Dose Establishment

The results of nonclinical studies showed that the no-observed-adverse-effect levels (NOAEL) of Engensis (VM202) were estimated to be ≥ 6.840 mg/kg in rats and ≥ 1.2 mg/kg in rabbits. No abnormal findings were shown in terms of safety and tolerability when 64 mg of VM202 in total was administered 4 times at one-week intervals (Weeks 0, 1, 2, and 3) in a phase 1/2 clinical study in patients with amyotrophic lateral sclerosis.

Thus, the administration dose of this clinical study in CMT1A patients was established as 14

mg per administration, for a total of 56 mg. Considering the residual quantity results in the phase 1/2 clinical study in patients with amyotrophic lateral sclerosis, an interval of 90 days was placed between the 1st dose and the 3rd dose.

4 Good Clinical Practice

This study shall be conducted in compliance with the approval of Samsung Medical Center Institutional Review Board (IRB) and relevant regulations such as the Declaration of Helsinki, Article 30 of the Regulation on Safety of Medicinal Products, etc. [implemented on March 23, 2013], and the Korean Good Clinical Practice for Medicinal Products. Therefore, the purpose of the study and the characteristics of the investigational product shall be described to the subjects through an information sheet. Only those volunteers who have completed the consent form, knowing the purpose of the study, its risks, etc., shall participate in the study. Furthermore, it shall be explained to the subjects that they may withdraw their consent for study participation, if they wish, at any time throughout the study. The results obtained during the study shall be recorded in the case report form, and confidentiality shall be ensured for all information. If an adverse event occurs, it shall be immediately reported to the person in charge of the clinical study. If necessary, the subject shall be allowed to receive inpatient tests and treatment, and follow-ups shall be performed until the symptoms disappear.

5 Clinical Study Plan

5.1 Purpose of Clinical Study

To evaluate the safety and tolerability of the investigational product (VM202) injected in the weakened lower limb muscles of CMT1A patients.

Primary Objective

To evaluate safety and tolerability following repeated doses of the investigational product (VM202).

Secondary Objectives

To evaluate efficacy following repeated doses of the investigational product (VM202).

(1) Changes in severity of disease
  - CMTNS-v2 (Charcot-Marie-Tooth Neuropathy Score version 2)
- FDS (functional disability scale)

(2) Changes in lower limb function:
  - ONLS (overall neuropathy limitation score) leg scale
  - 10MWT (10-meter walk test)

(3) Changes in fatty infiltration level of lower limb muscles:
  - MRI leg

(4) Nerve regeneration potential:
  - CMAP (compound motor nerve action potential)
  - SNAP (compound sensory nerve action potential)
  - NCV (nerve conduction velocity)

(5) HGF antibody generation by VM202

5.2 Clinical Study Design

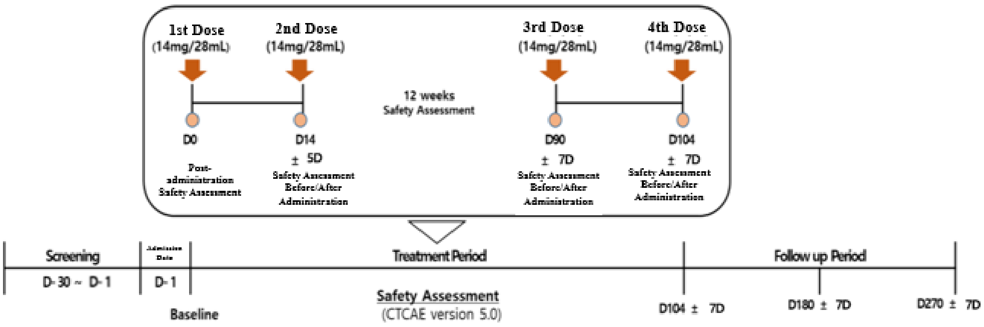

Figure 7. A schematic diagram of clinical study

This is a phase 1/2a, open-label, single-center study to evaluate for 270 days the safety and tolerability of intramuscularly injected VM202 in twelve CMT1A patients with symptoms of lower limb weakening. When subjects who have voluntarily signed the informed consent form are enrolled in this clinical study, they shall undergo tests to determine their eligibility for this clinical study.

Subjects who have satisfied the inclusion/exclusion criteria shall be given the investigational product 4 times (2 administration sessions for 2 weeks followed by a 3-month safety and tolerability assessment, and then 2 administration sessions for 2 weeks) in total for 104 days. Safety and tolerability assessments shall be performed at every visit, and efficacy assessments shall be performed at the 2nd visit (baseline, Day 0, prior to administration) and the 7th visit (termination visit, Day 270).

Although this is a phase 1/2a clinical study, it is intended to evaluate clinically meaningful endpoints in relation to efficacy considering that Charcot-Marie-Tooth disease type 1A (CMT1A) is a rare disease that still does not have an available treatment method.

5.3 Subjects

5.3.1 Target Number of Subjects and Rationale for Calculation

Using this investigational product, clinical studies have been conducted on six diseases in total since 2007 (see Table 2). Although there were no cases in which the drug was applied to CMT1A patients, a total of 18 subjects were recruited in a clinical study on amyotrophic lateral sclerosis (see section 3.8.5) among these studies. As a result of assessing safety, it is intended that this clinical study be conducted with 12 subjects, which is set less than the size of subjects in the study of amyotrophic lateral sclerosis because it is just addition

of the indication and the progression of CMT1A is slow although this clinical study also targets similar symptoms.

5.3.2 Inclusion Criteria

Subjects enrolled in this clinical study must satisfy all of the following inclusion criteria:

1) Males or females ≥ 19 years of age and ≤ 65 years of age
2) Patients with confirmed diagnosis of CMT1A by genetic testing
3) Patients with mild-to-moderate severity assessed by Charcot-Marie-Tooth Neuropathy Score version 2 (CMTNS-v2) with a score > 2 and ≤ 20
4) Individuals with lower limb muscle weakness with minimum dorsiflexion or more
5) Individuals who voluntarily consented to participate in this study and signed the IRB-approved informed consent form after listening to a description on the characteristics of this clinical study prior to all screening tests
6) Individuals who can comply with the requirements in the clinical study
7) In case of females of child-bearing potential, those who test negative in a urine or serum pregnancy test at screening
8) Individuals who practice medically approved contraceptive methods* throughout the clinical study

*Definitions

- Drugs: Oral contraceptives, skin patches, or progestin formulations (implants or injections)
- Barrier methods: Condoms, diaphragms, intrauterine devices (IUDs), vaginal suppositories
- Abstinences: Complete abstinence (However, periodic abstinence (e.g., calendar method, ovulation method, and sympto-thermal method) and self-restraint are not considered as acceptable methods of contraception.)

5.3.3 Exclusion Criteria

Subjects will be excluded from this clinical study if any one of the following criteria is met:

1) Patients with significant respiratory, circulatory, renal, gastrointestinal, hepatic, endocrine, hematologic, psychiatric disorders or other severe diseases, or alcohol or drug addiction who may develop safety issues or cause confusion in the interpretation of the clinical study results as determined by the principal investigator
2) Patients with other neuromuscular diseases or neuropathy-inducing factors: Patients with chronic alcohol addiction, undergoing anticancer chemotherapy, or taking neurotoxic drugs

3) Patients diagnosed with diabetes
4) Patients diagnosed with inflammatory bowel disease
5) Patients with a history of stroke or cerebral ischemic attack within 12 months prior to the screening date
6) Patients with a history of coronary artery disease, such as myocardial infarction and unstable angina pectoris, within 12 months prior to the screening date
7) Morbidly obese patients with body mass index (BMI) ≥ 37
8) Patients who underwent orthopedic surgery (corrective surgery for bone and ligament, artificial joint implantation, osteosynthesis, osteotomy, arthroscopic surgery) in the lower limbs within 6 months prior to the screening date
9) Patients who may be affected by the muscle strength measurement test due to ankle contracture or surgery
10) Patients with uncontrolled hypertension (if systolic blood pressure is ≥ 160 mmHg or diastolic blood pressure is ≥ 100 mmHg at screening)
11) Patients or patient's immediate family members (parents, siblings, offspring) with a history of malignant tumors within the last 5 years prior to the screening date, excluding basal cell carcinoma or squamous cell carcinoma that occurs on the skin (if it is determined that there is no possibility of relapse after resection), or with a family history of familial adenomatous polyposis (FAP) or hereditary nonpolyposis colorectal cancer (HNPCC)
12) Patients who have not completed a national cancer screening program applicable to their sex and age (if it cannot be confirmed that the relevant test was received at a national cancer screening center or a recognized screening center)

However, if it is confirmed that the relevant test was received at a national cancer screening center or a recognized screening center during the screening period, and that the results were within normal range, the patients may participate in the clinical study.

Common to males and females: If a patient is ≥ 50 years of age, the results of a colonoscopy within 5 years prior to the screening must be determined as being within normal range, and if adenomatous polyps are evident, the results of a colonoscopy within 1 year must be determined as being within normal range (inflammatory polyps or hyperplastic polyps are included in the normal range). If a patient is ≥ 40 years of age, the results of a gastroscopy within 2 years prior to the screening must be within normal range. If a patient is ≥ 54 years of age and has a 30 pack-year history of smoking or more, the results of a low-dose chest CT within 2 years prior to the screening must be within normal range. In case of liver cancer,

carriers of hepatitis B or hepatitis C virus and patients with hepatic cirrhosis fall under the exclusion criteria.

Females: For females ≥ 40 years of age, normal range findings must be confirmed in a mammogram within 2 years. For females ≥ 20 years of age, normal range findings must be confirmed in a Pap smear within 2 years.

13) Patients diagnosed with active pulmonary tuberculosis
14) Patients with HBV or HCV
15) Patients who test positive in human immunodeficiency virus (HIV) antibody test
16) Patients in an immunosuppressive state due to treatments such as immunosuppressants, chemotherapy, and radiotherapy
17) Patients with a history of mental disease within 6 months prior to the screening date, which may interfere with participation in the study
18) Patients who must take medications, that are known to have significant drug interactions within 14 days after the first administration of the investigational product or deemed unsuitable by the investigator's judgment
19) Individuals who participated in another clinical study* within 6 months before the time of screening

*Definitions

- Drug: Those who participated in another clinical study within 6 months before the time of screening shall be excluded.
- Medical device: Those who participated in a noninvasive clinical study may participate in this clinical study if the principal investigator determines that the safety or pharmacodynamic assessment will not be affected.

20) Individuals who have shown significant adverse events such as hypersensitivity reactions to the investigational product
21) Pregnant or breastfeeding females
22) Other individuals determined ineligible by the principal investigator to participate in the clinical study due to other reasons including clinical laboratory test results

5.3.4 Assignment of Subject Numbers

Subjects that will be voluntarily participating in this clinical study shall be assigned screening numbers (completion of informed consent form - checking eligibility with inclusion/exclusion criteria), and shall be assigned allocation numbers if they satisfy the inclusion/exclusion criteria.

A screening number shall start with the letter "S" and consist of a five-digit number, and it shall be assigned base on the order of the subjects' written consent at the institution.
For example, the meaning of "S01001" is as follows:

S	01	001
Screening number (Acronym of screening)	Institution (Indicated as "01" since it is a single center)	Screening number

Allocation numbers shall be assigned, in the order of enrollment in the clinical study, to the subjects who have satisfied the inclusion/exclusion criteria after completing all screening tests. The allocation number shall start with the letter "A" followed by a five-digit number.
For example, the meaning of "A01001" is as follows:

A	01	001
Allocation number (Acronym of allocation)	Institution (Indicated as "01" since it is a single center)	Allocation number

5.4 Contraindicated Medications

This clinical study shall be conducted in CMT1A patients with symptoms of weakness in lower limbs, and the list of contraindicated medications for CMT patients presented by the Charcot-Marie-Tooth Association (CMTA) and the CMT Research Foundation is as shown below. Since CMT is a congenital peripheral neuropathy that still does not have a cure, the best method is slowing the progression of disease. Neurotoxic drugs that can accelerate the damage of peripheral nerves shall be contraindicated, and the contraindicated medications are classified into 4 groups as shown in the following Table 5 depending on their risk level.
After the subjects sign the informed consent form, they shall be provided "Appendix 2. Contraindicated Medications" as an addendum.

Table 5. Contraindicated medications

Ingredient Name	Product Name	Indication
Definite High Risk		
**Vinca alkaloids (vincristine)**	Vincristine sulfate injection, Vincran Injection,	Acute leukemia, malignant lymphoma, malignant tumor
**Taxols**		
**Paclitaxel**	Taxol Injection, Paclitaxel injection, Neotax Injection,	Advanced prostate cancer

Ingredient Name	Product Name	Indication
	Sandoz Paclitaxel Injection	
**Docetaxel**	Taxotere Injection, Taxozen Injection, Dotaxel Injection	Breast cancer, prostate cancer, lung cancer
**Cabazitaxel**	Jevtana Injection	Breast cancer, lung cancer, ovarian cancer, esophageal cancer
Moderate to Significant Risk		
**Amiodarone**	Codarone Injection, Codarone Tablet	Ventricular arrhythmia (fibrillation, tachycardia)
**Arsenic trioxide**	Trisenox Injection	Acute promyelocytic leukemia
**Bortezomib**	Velcade Injection, Tezobel Injection, Protezomib Injection	Multiple myeloma
**Brentuximab vedotin**	Adcetris Injection	Hodgkin lymphoma, systemic anaplastic large cell lymphoma
**Cetuximab**	Erbitux Injection	Head and neck cancer, colon cancer
**Cisplatin**	Cisplan Injection, Unistin Injection	Bladder cancer, ovarian cancer, testicular cancer
**Colchicine**	Colchicine Tablets, Colchinine Tablets	Gout prevention, gouty arthritis
**Dapsone**	Dapsone Tablets	Bullous herpetiformis dermatitis, Hansen's disease
**Didanosine; Dideoxyinosine (ddI)**	Videx EC SR Capsule (distribution currently discontinued)	HIV infection
**Dichloroacetate (DCA)**	Dichloroacetate sodium	Chronic lactic acidosis
**Disulfiram**	Alcoholstop (distribution currently discontinued)	Alcohol dependence
**Eribulin mesylate**	Halaven Injection	Metastatic breast cancer
**Ipilimumab**	Yervoy Injection	Melanoma
**Ixabepilone**	Ixempra (distribution currently discontinued)	Metastatic or locally advanced breast cancer
**Leflunomide**	Arava Tablets, Rualba Tablets, Durova Tablets	Rheumatoid arthritis
**Lenalidomide**	Revlimid Capsule, Lenaloma Capsule, Lenalid Tablets	Myelodysplastic syndrome
**Metronidazole**	Flasinyl Tablets, Trizele Injection, Flagyl Injection	Bacterial infection of large intestine, small intestine, vagina, and others; bacteremia, peritonitis, endocarditis
**Misonidazole**	F-18 fluoromisonidazole	PET/PET-CT imaging radiotracer
**Nitrofurantoin; Macrodantin**	Boryung Nitrofurantoin capsules	Urinary tract infection
**Nitrous oxide**		General anesthesia
**Nivolumab**	Opdivo Injection	Melanoma, advanced renal cell carcinoma
**Oxaliplatin**	Eloxatin Injection, Oxapla Injection, Oxaplin Injection	Colorectal cancer
**Pembrolizumab**	Keytruda Injection	Melanoma, non-small cell

Ingredient Name	Product Name	Indication
		lung cancer
**Perhexiline**	Pexsig (not distributed in South Korea)	Angina pectoris
**Pomalidomide**	Pomalyst Capsule	Multiple myeloma
**Pyridoxine**	Licopyri Injection, Vita B6 Injection, Plidoxine Tablets	When overdosed by 10X or more (indication: vitamin B6 deficiency, drug-induced neuropathy); However, there is no issue with intake through food.
**Stavudine (d4T)**	Zerit (distribution currently discontinued)	HIV infection
**Suramin**	Antrypol, Moranyl	African trypanosomiasis, onchocerciasis
**Thalidomide**	Celgenethalidomide Capsule, Taligrov Capsule	Erythema, lepromatous nodules
**Zalcitabine (ddC)**	Hivid (distribution currently discontinued)	HIV infection
**Fluoroquinolones**		
**Ciprofloxacin**	Ciprobay Injection, Qupron Tablet, Cycin Injection	Bacterial infections (skin, bone, blood, urinary tract, respiratory, gastrointestinal tract infections), pneumonia, sinusitis, typhoid
**Enoxacin**	Flumark (distribution currently discontinued)	Urinary tract infection, gonorrhea
**Gatifloxacin**	Gatiflo (distribution currently discontinued)	Pneumonia and bronchitis; paranasal sinus, respiratory, urinary tract infections; sexually transmitted diseases
**Levofloxacin**	Levocacin Tablet, Levoroxin Injection, Levofexin Injection	Infections (skin, respiratory, urinary tract), venereal diseases
**Moxifloxacin**	Avelox Injection, Remox Tablets, Moveloxin Injection	Infections (skin, respiratory)
**Norfloxacin**	Baccidal Tablets, Urekacin Capsule, Newsadal Tablets	Urinary tract infection, prostatitis, gonorrhea
**Ofloxacin**	Fugacin Tablets, Effexin Injection, Ofloxacin Tablets	Infections (urinary tract, skin, bone, cardiac)
**Sparfloxacin**	Sparoxin (distribution currently discontinued)	Lower respiratory tract infection
**Trovafloxacin (Alatrofloxacin)**	Trovan (distribution currently discontinued)	Pneumonia, infections (abdomen, pelvis, skin)
**Gold salts**		
**Auranofin**	Ridaura (distribution currently discontinued)	Rheumatoid arthritis
**Aurothioglucose**	Solganal (distribution currently discontinued)	Rheumatoid arthritis
**Gold sodium thiomalate**	Aurolate (distribution currently discontinued)	Rheumatoid arthritis
Uncertain or Minor Risk		

Ingredient Name	Product Name	Indication
**5-fluorouracil**	Efficil-Injection	Breast cancer, colorectal cancer, gastric cancer, pancreatic cancer
**Doxorubicin**	Adriamycin-PFS Injection, Adriamycin-RDF Injection	Leukemia, lymphoma, malignant tumor
**Almitrine**	Vectarion (distribution currently discontinued)	Acute respiratory failure
**Chloroquine**	Araren phosphate, Araren hydrochloride (distribution currently discontinued)	Amebiasis, malaria
**Cytarabine (Ara-C; cytosine arabinoside); Cytarabine liposomal**	Cytarabine Injection, Cytosar-U Injection, DepoCyt Injection	Leukemia, lymphomatous meningitis
**Ethambutol**	Tambutol Tablets, Myambutol Tablets	Tuberculosis
**Etoposide (VP-16)**	Lastet Capsule, EPS Injection, Etopul Injection	Small cell lung cancer, refractory testicular neoplasm
**Gemcitabine**	Gemzar Injection, Gemtan Injection, Gembin Injection	Non-small cell lung cancer, pancreatic cancer
**Griseofulvin**	Fulvicin Tablets,	Dermatophytosis, tinea capitis, tinea unguium
**Altretamine**	Hexalen (distribution currently discontinued)	Ovarian cancer
**Hydralazine**	Hydralazine HCl Injection	Hypertension
**Ifosfamide**	Holoxan Injection	Testicular germ cell tumor, osteosarcoma, soft tissue sarcoma
**Infliximab**	Remicade Injection, Remsima Injection, Remaloce Injection	Rheumatoid arthritis, Crohn's disease
**Isoniazid (INH)**	Aina Tablet, Yuhanzid Tablets	Tuberculosis
**Lansoprazole**	Lanozol Tablets, Lancid Capsule, Lanster Capsule	Peptic ulcer, gastroesophageal reflux disease, Zollinger-Ellison syndrome
**Mefloquine**	Lariam Tablets	Malaria prevention and treatment
**Omeprazole**	OMP Tablet, Omeprazole Capsule	Peptic ulcer, gastroesophageal reflux disease, Zollinger-Ellison syndrome
**Penicillamine**	Artamin Capsule	
**Phenytoin**	Phenytoin Tablets, Hydantoin Tablets, Phenytoin sodium injection	Epilepsy, convulsion, seizure
**Podophyllin resin**	Podocon-25, Podofin, Podofilm	External genital or perianal warts due to HPV
**Tacrolimus (FK506)**	Prograf Capsule, Tarimus Capsule, Tacrobel Injection	Prevention of organ transplant rejection (liver, kidney, and other organs)
**Zimeldine**	(distribution currently discontinued)	Depression
**α-interferon**	Roferon A Prefilled Injection, Interferon Alpha-2 Injection	Hepatitis B, hepatitis C, HPV warts, Kaposi sarcoma, renal cell carcinoma, malignant

Ingredient Name	Product Name	Indication
		melanoma (skin cancer), non-Hodgkin lymphoma
**Sertraline**	Zoloft Tablets, Seltra Tablets, Traline Tablets	Depression, panic attack, obsessive-compulsive disorder, post-traumatic stress disorder, social disorder
**Statins**		
**Atorvastatin**	Lipitor Tablets, Atosta Tablets, Lipikhan Tablets	Hypercholesterolemia
**Fluvastatin**	Lescol Capsule, Lescol XL Tablets, Xilep Capsule	Hypercholesterolemia
**Lovastatin**	Lovastatin Tablets, Lovalord Tablets, Byrotin Tablets	Hypercholesterolemia
**Pravastatin**	Mevalotin Tablets, Prastan Tablets, Pravastar Tablets	Hypercholesterolemia
**Rosuvastatin**	Crestor Tablets, Rosvatin Tablets, Allstatin Tablets	Hypercholesterolemia
**Simvastatin**	Zocor Tablets, Simvast Tablets, Newvastin Tablets	Hypercholesterolemia
Negligible or Doubtful Risk		
**Allopurinol**	Zyroric Tablets, Allopurinol Tablets	Gout, gouty arthritis, nephrolithiasis, urolithiasis
**Amitriptyline**	Elavil Tablets, Enafon Tablets, Amitriptyline HCl Tablets	Depression
**Chloramphenicol**	Helocetin Injection	Meningitis, typhoid
**Chlorprothixene**	Taractan (distribution currently discontinued)	Psychosis
**Cimetidine**	Tagamet Injection, Cimetidine Tablets H-2 Tablets	Gastric and duodenal ulcers, gastroesophageal reflux
**Clioquinol**	Vioform (ointment, cream, ear drops)	dermatitis, folliculitis, eczema, tinea pedis, tinea cruris, dermatophytosis
**Clofibrate**	Atromid (distribution currently discontinued)	Hyperlipidemia
**Cyclosporin A**	Sandimmun Injection, Cipol Injection, Thymune Injection	Prevention of organ transplant rejection, severe psoriasis and rheumatoid arthritis
**Enalapril**	Ecaril Tablets, Enaprin Tablets, Pril Tablets	Hypertension, congestive heart failure
**Gluthethimide**	Doridem (distribution currently discontinued)	Insomnia
**Lithium**	Lithium carbonate Tablets, Lithan Tablets	Bipolar disorder
**Phenelzine**	Nardil (distribution currently discontinued)	Depression
**Propafenone**	Ritmol Tablets, Rytmonorm SR Capsule, Profenone Tablets	Atrial fibrillation, ventricular arrhythmia
**Sulfonamides**		
**Sulfacetamide**	Rosula Aqueos gel, Sulfaclan lotion	Acne, seborrheic dermatitis, eye and vaginal infections
**Sulfabenzamide**	Sultrin cream	Vaginitis

Ingredient Name	Product Name	Indication
**Sulfadiazine**	Virocide tablets (not distributed in South Korea)	Toxoplasmosis
**Sulfamethoxazole**	Vactoral Tablets	Urinary tract infection, otitis media
**Sulfasalazine**	Sazopin Tablets, Jopirin Enteric Coated Tablets, Salazine Tablets	Rheumatoid arthritis, ulcerative colitis
**Sulfathiazole**	Sultrin cream	Vaginitis
**Sulfisoxazole (Sulfafurazole)**	Gantrisin, Neoxazol, Sulfizole tablet	Urinary tract infection, otitis media

5.5 Termination of Clinical Study

5.5.1 Study Termination for Subjects

If a subject has completed all tests corresponding to the assessment index throughout the study period of 270 days (9 months) according to this clinical study protocol, the subject shall be deemed to have terminated the study.

5.5.2 Subject Drop-out

The subjects may withdraw their consent for study participation at any time during the clinical study, and they shall not face any disadvantages even if they do so. In addition, if the investigator determines that continuing to participate in the clinical study is harmful to a subject or that a subject refuses to follow the instructions of the investigator, the clinical study for the subject may be discontinued.

The study may be suspended for the following reasons:

- Adverse events that require discontinuation of the study (before treatment)
- A subject is lost to follow-up
- Decision of the subject
- Decision of the investigator
- Other reasons

All reasons for the study discontinuation of subjects shall be recorded in the study termination form of the case report form.

5.5.3 Early Termination of Clinical Study

The sponsor has the right to discontinue the clinical study at any time throughout the clinical study for reasons such as safety, ethics, or management issues.

(1) Early discontinuation by the sponsor or investigator

This clinical study may be discontinued early at any time for safety, behavioral, or administrative reasons depending on the decision of the investigator or the sponsor.
The cases in which the investigator or the sponsor early discontinues the clinical study are as follows:

1) If the institution fails to enroll the target number of subjects;
2) If efficacy and safety information that can significantly impact the continuation of the clinical study emerges;
3) If it is difficult to conduct an appropriate clinical study because the institution or the investigator has violated the Korea Good Clinical Practice (KGCP), the clinical study protocol, contractual matters, etc.;
4) If there are other administrative reasons that can have a significant impact on the continuation of the clinical study.

(2) Permanent discontinuation of administration of investigational product

The cases in which administration of the investigational product is permanently discontinued are as follows but are not limited thereto:

1) If the subject or his or her representative withdraws consent;
2) If it is difficult to continue the clinical study due to adverse events;
3) If violations of the inclusion and exclusion criteria are found while conducting the clinical study;
4) If it is determined that taking contraindicated medications or treatment with contraindicated pharmaceuticals is necessary;
5) If contraindicated therapies were performed, or if it is determined that performing contraindicated therapies is necessary;
6) If subject follow-up is not possible;
7) If serious violations of the protocol are found;
8) If another disease is found in the subject that makes it impossible to conduct the tests and procedures performed at regular visits;
9) If the investigator determines that continuing the clinical study is difficult due to other reasons.

In order not to miss the occurrence of adverse events which the investigator does not know about, all methods such as phone calls, letters, and direct visits shall be utilized to

directly contact the subjects who refuse to visit. The reasons for early discontinuation or termination of administration shall be recorded in the case report form. Subjects who have early discontinued or have terminated administration may not participate again.

For subjects who have early terminated the study, the tests for early termination in "Summary of Study Schedule" shall be performed.
If the clinical study has been early discontinued, the following shall be performed:

1) If the principal investigator has early terminated or suspended the clinical study without a prior agreement with the sponsor, the principal investigator shall immediately inform this fact to the sponsor and the Institutional Review Board, and shall submit a detailed statement of reasons for the early termination and suspension.
2) If the sponsor has early terminated or suspended the clinical study, the principal investigator shall immediately inform this fact to the Institutional Review Board, and shall submit a detailed statement of reasons for the early termination and suspension.
3) If the Institutional Review Board has early terminated or suspended the clinical study, the principal investigator shall immediately inform this fact to the sponsor, and shall submit a detailed statement of reasons for the early termination and suspension.
4) If the relevant clinical study has been early terminated or suspended as in 1) through 3) in accordance with regulations, the principal investigator shall immediately inform this fact to the subjects, and shall ensure that appropriate measures are taken and follow-ups are conducted.

For subjects who have been discontinued due to adverse events, follow-ups shall be conducted until the investigator determines that the corresponding adverse events are resolved or the subjects are medically stable. For these subjects, Helixmith Co., Ltd. and the investigators shall try their best to provide standard clinical treatment. Helixmith Co., Ltd. shall provide indemnification, if applicable, in accordance with the subject indemnification policy. In addition, the tests (10 items) mentioned above shall be performed for the safety of the subjects.

If a subject cannot be contacted in follow-ups, the investigator shall attempt to contact (phone call, email, etc.) the subject at least three times. If there is no response from the subject despite this, the investigator shall contact a family member of the subject or his or her primary physician. The evidence of such attempted contact shall be recorded in the source data, and the confirmation of receipt of letters sent to the subject can be an example of such proof.

5.6 Information About and Management of Investigational Product

5.6.1 Information About and Management of Investigational Product

Table 6. Information about and storage conditions of investigational product

Product Name or Code Name	Code name: Engenesis: VM202
Manufacturer of Drug Substance	Manufacturer: Cobra Biologics Ltd.<br>Address: Stephenson Building The Science Park Keele, Staffordshire<br>ST5 5SP UK
Manufacturer of Drug Product	Manufacturer: Ajinomoto Althea, Inc.<br>Address: 11040 Roselle Street San Diego, CA 92121 USA
Dosage Form and Appearance	It is a white or an almost white lyophilized powder contained in a colorless transparent vial that becomes a colorless transparent solution when dissolved with water for injection.
Drug Substances and Their Quantities	In 1 vial (see below)
Storage Method	Store under refrigeration (2–8°C)

In 1 vial

Function	Ingredient	Quantity
Active ingredient	pCK-HGF-X7	2.5 mg/vial
Additive (excipient, stabilizer)	Sucrose (KP)	55.0 mg (1.1%)*
	Sodium chloride (KP)	45.0 mg (0.9%)*

5.6.2 Dose, Administration Route, and Administration Method

Administration route of investigational product: Intramuscular injection

Preparation of investigational product:

The sterilized/frozen investigational product contains 2.5 mg of the active ingredient "pCK-HGF-X7" per vial. The investigator shall dilute (0.5 mg/mL) the investigational product by using 5.0 mL of water for injection, and then 56 intramuscular injections in total shall be performed at 3 sites on the left/right lower limb for each subject as shown in Table 7.

Precautions:

The diluted vial shall be used for only one subject.

Using a needle appropriate for intramuscular injections (e.g., 29 gauge, 1/2 inch or 1 inch in length depending on the muscle type and subcutaneous fat thickness), the injections shall be evenly distributed on the target muscle as shown in Table 8 while avoiding the fascia.

Table 7. Total number of investigational product vials, doses, and injections per subject at each visit

Number of Vials per Visit Day	Number of Injections per Visit Day	Visit day/ Total Administration Dose
7	56	14mg/ 28mL

Table 8. Number of injections and administration dose per target muscle (injection site)

Target Muscle		Administration Dose per Target Muscle (mg), (number of injections per muscle: left/right)				Total Administration Dose (mg), (total number of injections: left/right)
		1st Dose	2nd Dose	3rd Dose	4th Dose	
		D 0	D14	D90	D104	
Lower leg	Peroneus longus	3, (6/6)	3, (6/6)	3, (6/6)	3, (6/6)	12, (24/24)
	Gastrocnemius	6, (12/12)	6, (12/12)	6, (12/12)	6, (12/12)	24, (48/48)
	Tibialis anterior	5, (10/10)	5, (10/10)	5, (10/10)	5, (10/10)	20, (40/40)
**Final Administration Dose (mg) (number of injections for left/right)**		**14, (28/28)**	**14, (28/28)**	**14, (28/28)**	**14, (28/28)**	**56, (112/112)**

See "Appendix 3. Administration Method of Investigational Product" for details.

5.6.3 Labeling and Packaging

A label in Korean describing the following details shall be attached to the packaging container for the investigational product.

- Subject number
- Marked as "For clinical study use only"
- Code name of the product or generic name of the active ingredient
- Clinical study identification code
- Batch number and shelf (effective) life
- Storage method
- Name of clinical study sponsor
- Marked as "Do not use for purposes other than clinical studies"

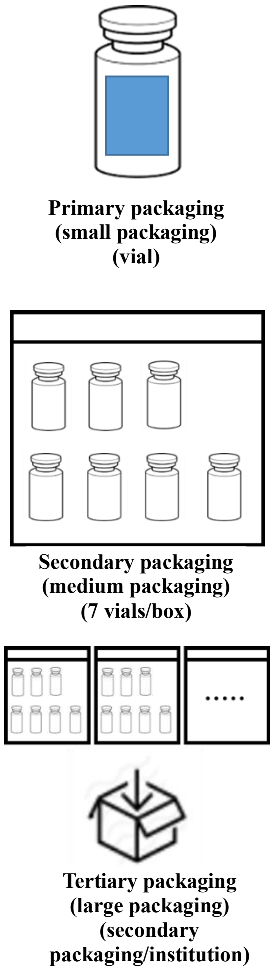

Primary packaging

VM202 is supplied in a frozen state in a sterilized glass vial containing 2.5 mg each.

Secondary packaging

For the subjects to be allocated, seven vials shall be packaged in a box for each administration visit.

Tertiary packaging

The investigational products in secondary packaging shall be packaged in boxes and delivered to the clinical trial pharmacist of the institution.

Figure 8. Summary of investigational product packaging

5.6.4 Handling and Preparation of Investigational Product

The investigational product shall be imported and relabeled by the clinical study sponsor, Helixmith Co., Ltd., and provided to the investigator in charge at the institution. At this time, it shall be prepared only by those who are authorized at the institution, and shall be administered only to the subjects of this clinical study.

5.6.5 Accountability Management, Collection and Disposal of Investigational Product

In accordance with the Korea Good Clinical Practice (KGCP), all investigators shall document and manage records until the investigational product is destroyed.

The investigational product shall be used only under the management of the principal investigator in accordance with the details of this protocol.

The clinical trial pharmacist or a designated individual shall accurately record the date of receipt and details when receiving the investigational product.

As the clinical trial pharmacist shall store and manage the investigational products by recording the allocation number, prescription date, released quantity, etc., in the drug accountability log, the clinical trial pharmacist shall verify the usage status and store a list of receipt, issuance, and return records for the drug used in the clinical study. This accountability log of the investigational product must be verifiable at any time, and the clinical trial pharmacist shall provide a copy of this record to the study sponsor when the study is terminated.

A clinical study monitor shall regularly view the management log to verify the usage details of all investigational products.

Even after termination of the clinical study, the clinical trial pharmacist shall verify the inventory of all investigational products including the investigational products used in the clinical study, unused investigational products, and partially used investigational products, and if there are unused investigational products remaining, they shall be returned to the study sponsor, and the study sponsor shall verify the total quantity in writing.

5.7. Management of Coronavirus Disease-19 (COVID-19)

5.7.1 Selection and enrollment of clinical study subjects

Not applicable because enrollment of all subjects has been completed for this study.

5.7.2 Management of clinical study subjects

Regarding COVID-19, this clinical study is managed as follows:

- Subjects who have been confirmed to have COVID-19 or who have been in close contact with COVID-19 patients, must be quarantined for 14 days whether or not symtons have occurred. After being released from quarantine, the subject continues with the procedures for the visit period.
- Subjects who have been confirmed to have COVID-19 patients after dosing but have recovered prior to their next visit may continue to participate in the study.
- Subjects who have been confirmed to have COVID-19 patients after dosing but have not recovered prior to their next visit must suspend their participation in the study and continue with the procedures for the visit period after recovering. Subjects who have not recovered until Day 270 ± 7 should discontinue their participation in the

study. All reasons for discontinuation should be recorded in the study termination form of the Case Report Form.

- If it is deemed by the investigator that the subject confirmed with COVID-19 is no longer able to participate in the study due to any reason, the investigator shall discontinue the patient's study. All reasons for discontinuation should be recorded in the study termination form of the Case Report Form.

- The fact that subjects of this clinical study have been confirmed to have COVID-19 is recorded as an adverse event on the Case Report Form and classified as Adverse Events of Special Interest (AESI).

5.7.3 Records of Coronavirus Disease-19 (COVID-19)

If the protocol is not complied with due to coronavirus Disease-19 (COVID-19), COVID-19 shall be clearly recorded and managed as a reason for non-compliance on the relevant form of the case report form.

- Adeverse Event Form
- Study Termination From
- Date of visit and Evaluation Form
- Other

5.8 Retrospective Biomarker Study

Blood samples are collected at Day 0, Day 90, Day 180 and Day 270 (or early termination visit) for retrospective biomarker study of CMT. Collected samples will be processed according to detailed procedure in a separate protocol.

5.8.1 Purpose of the study

Changes in peripheral neuropathy biomarker (p62, p75, NCAM)

- Serum p62 (p62/sequestosome-1) concentration
- Serum p75 (p75 neurotrophin receptor) concentration
- Serum NCAM (neural cell adhesion molecule 1) concentration

5.8.2 Method of the study

After explaining the purpose of the biomarker study and preservation period of blood samples to the subject, the blood of the subject who voluntarily signed the consent form for research on human materials, which is a 34th appendix form in the Enforcement Rule of Bioethics and Safety Act, is retrospectively analyzed.

5.8.3 Methods for anonymizing human materials and measures to protect personal information

After the subject's voluntary consent, the collected blood samples are stored with unique numbers after all personal information is deleted. The preservation period of human materials is in accordance with the consent form for research on human materials.

6 Clinical Study Procedures and Assessments

6.1 Visit Schedule and Observation Items

Schedule	Day -30 to Day -1	Day -1	Day 0		Day 14 ± 5		Day 30 ± 7	Day 90 ± 7		Day 104 ± 7		Day 180 ± 7	Day 270 ± 7	Early Termination	Unscheduled Visit
Visit No.	Visit 1	Visit 2 (Hospitalization)			Visit 3		Outpatient follow-up if abnormal test results are present on Day 14 (Second Administration)*	Visit 4		Visit 5		Visit 6	Visit 7		
Specific Notes	Screening	Hospitalization Day[15)]	First Administration		Second Administration			Third Administration		Fourth Administration		Outpatient Follow-up	Outpatient Follow-up		
			Before Administration	After Administration[16)]	Before Administration	After Administration[16)]		Before Administration	After Administration[16)]	Before Administration	After Administration[16)]				
Informed consent form	X														
Subject background survey[1)]	X														
Medical history survey[2)]	X														
Physical examination[3)]	X														
Body measurement (weight measurement)[4)]	X		X		X		X*	X		X		X	X	X	X
Virus serology test[5)]	X														
Complete blood cell count and general blood chemistry tests[6)]	X		X		X		X*	X		X		X	X	X	X
Retrospective biomarker study[7)]			X					X				X	X	X	
Chest X-ray (PA)[8)]	X														
Urinalysis (U/A)[9)]	X		X		X		X*	X		X		X	X	X	X

Schedule	Day -30 to Day -1	Day -1	Day 0		Day 14 ± 5		Day 30 ± 7	Day 90 ± 7		Day 104 ± 7		Day 180 ± 7	Day 270 ± 7	Early Termination	Unscheduled Visit
Visit No.	Visit 1	Visit 2 (Hospitalization)			Visit 3		Outpatient follow-up if abnormal test results are present on Day 14 (Second Administration)*	Visit 4		Visit 5		Visit 6	Visit 7		
Specific Notes	Screening	Hospitalization Day[15]	First Administration		Second Administration			Third Administration		Fourth Administration		Outpatient Follow-up	Outpatient Follow-up		
			Before Administration	After Administration[16]	Before Administration	After Administration[16]		Before Administration	After Administration[16]	Before Administration	After Administration[16]				
Urine or serum pregnancy test[10]	X														
Electrocardiogram[11]	X														
Anti-HGF Ab			X										X	X	
Vital signs	X	X[15]	X	X	X	X	X*	X	X	X	X	X	X	X	X
Concomitant medications survey[12]	X		X		X		X*	X		X		X	X	X	X
Neurologic exam	X		X		X		X*	X		X		X	X	X	
CMTNS-v2	X		X										X	X	
MRI leg[13]			X										X	(X)	
FDS, ONLS (leg), 10MWT			X					X				X	X	X	
CMAP, SNAP, NCV			X										X	X	
Use of assistive device	X												X	X	
IP administration**			X		X			X		X					
Adverse event assessment[14]				X	X	X	X*	X	X	X	X	X	X	X	X

* PI shall determine whether test results are abnormal and whether Day 30 follow-up testing is required.

** IP administration: Administration shall be performed after proceeding with all pre-administration assessment items.

1) Subject background survey: A survey shall be performed on demographic information and history of alcohol and tobacco use, etc.
2) Medical history survey: A survey shall be performed on medical history within 6 months before Visit 1 (screening). However, medical history/treatment history related to cancer shall be surveyed regardless of the time period. Clinically significant medical conditions or abnormalities observed during the period from the obtainment of the informed consent form until the administration of the investigational product shall be deemed as medical history. It shall be surveyed whether the national cancer screening (gastric cancer, colon cancer, liver cancer, lung cancer, cervical cancer, breast cancer) examinations relevant to the patient's age are taken and the results are within the normal range. In case of cervical cancer, if it cannot be confirmed that the national cancer screening examinations were taken within 2 years and the results are within the normal range, an examination and a pap smear shall be performed at the institution to verify normal range results.
3) Physical examination: Information shall be collected for examination items consisting of external appearance, skin, head/neck, chest/lungs, heart, abdomen, urinary/reproductive system, limbs, musculoskeletal system, nervous system, lymph nodes, and other items.
4) Body measurement (weight measurement): Height, weight, and BMI shall be measured. Height and BMI shall be measured only at screening (Visit 1).
5) Virus serology test: HIV, HBsAg, Anti-HBs, Anti-HCV
6) Complete blood cell count and general blood chemistry tests: The laboratory test items are as follows:
   - Complete blood cell count: WBC, RBC, Hb, Hct, MCV, MCH, MCHC, PLT, ESR, MPV, differential count of WBC (Band neutrophil, Segmented neutrophil, Eosinophil, Basophil, Lymphocyte, Monocyte)
   - General blood chemistry test: total protein, albumin, globulin, A/G ratio, cholesterol, total bilirubin, AST, ALT, fasting glucose, BUN, creatinine, estimated GFR, Ca^{2+}, phosphate, Na^{+}, K^{+}, Cl^{-}, CRP, triglyceride, HDL-cholesterol, LDL-cholesterol
7) Retrospective biomarker study : Blood samples are collected at Day 0, Day 90, Day 180 and Day 270 (or early termination visit) for retrospective biomarker study of CMT. Collected samples will be processed according to detailed procedure in a separate protocol. CMT 질환의 후향적 생물학적 표지자 연구를 위하여 Day 0, Day 90, Day 180, Day 270 (또는 조기 종료 방문)에 혈액을 수집한다. 수집된 혈액은 별도의 연구계획서에서 구체적인 연구방법에 따라서 진행될 것이다.
8) Chest X-ray (PA): At Visit 1 (screening), chest PA X-ray shall be performed to verify whether active tuberculosis is present. The results within 1 month (30 days) before Visit 1 may be used.
9) Urinalysis: The laboratory test items are as follows:
   - Color, turbidity, specific gravity, pH, albumin, glucose, ketones, bilirubin, blood, urobilinogen, nitrite, leukocyte esterase, microscopy (RBC, WBC, casts)
10) Urine or serum pregnancy test At Visit 1 (screening), urine or serum pregnancy tests shall be performed in females of childbearing potential (from post-menarche females to females ≥ 50 years of age within 1 year of menopause, or from post-menarche females to females < 50 years of age within 2 years of menopause). However, patients with surgical menopause (hysterectomy, bilateral oophorectomy, etc.) or who underwent sterilization surgery (bilateral tubal ligation, bilateral tubectomy) may be excluded. Menopause refers to the state after 1 year of amenorrhea.
11) Electrocardiogram: The electrocardiogram to be performed at Visit 1 (screening) may use results within 4 weeks before Visit 1 (screening).
12) Concomitant medications survey: All medications and treatments administered within 6 months before Visit 1 (screening) shall be surveyed. Previous medications and previous treatments shall be defined as all previously collected medications and treatments before Visit 2 (first administration of investigational product). Concomitant medications and

treatments shall refer to all medications that have been administered at least once starting from Visit 2 (first administration of investigational product) and throughout the clinical study. The categories of collected medications and treatments shall follow this definition. Whether the medications and treatments being administered should be continued shall be investigated at each visit.

13) MRI leg: The muscles of lower limbs shall be imaged, and the fatty infiltration level of the leg muscles injected with the investigational product shall be measured and evaluated as fat content value (%) at one level for each muscle. Considering the schedule, etc., of the institution, it shall be performed optionally at the early termination visit, and if it is not performed, the reasons shall be recorded in the case report form.
14) Adverse event assessment: At Visits 3, 4, and 5, an assessment of adverse events that have occurred since the last visit shall be performed prior to administering the investigational product, and localized adverse events shall be assessed at 2 ± 1 hours after administering the investigational product, as well as on the day after administration.
15) Hospitalization: Subjects shall be hospitalized on the day before administration of the investigational product and their vital signs shall be measured. If they are not hospitalized, vital signs may be omitted, and the hospitalization day in the case report form shall be recorded the same as the test day prior to administration.
16) After administration: Vital signs and adverse events shall be assessed at 2 ± 1 hours after administering the investigational product. The presence or absence of localized adverse events shall be verified on the day after administering the investigational product.

6.1.1 Visit 1 (screening, from Day -30 to Day -1)

The following items shall be performed on only those subjects that made the screening visit and provided voluntary consent.

- **Completion of informed consent form**
  The responsibilities that should be complied with by a subject throughout the study shall be explained. If a subject decides to voluntarily participate in the study and consents to the various tests and procedures that the subject will be receiving throughout the clinical study, the subject's handwritten signature shall be obtained on an already prepared informed consent form. The subject and the investigator shall sign the informed consent form and fill in the date. The original signed informed consent form shall be stored with the subject's records, and one copy shall be provided to the subject.

- **Assignment of subject screening number**
  See section 5.3.4 Assignment of Subject Numbers

- **Background survey**
  At Visit 1 (screening), a survey shall be performed on demographic information (sex, age, etc.), smoking history, alcohol consumption history, etc.

- **Medical history survey**
  Medical records on the medical history within 6 months before Visit 1 (screening) shall be obtained, and all positive/negative results shall be recorded in detail in the case report form. However, medical history/treatment history related to cancer shall be surveyed regardless of the time period. New results found at Visit 1 (screening) and Visit 2 (before administration of investigational product) shall be considered a part of medical history and shall not be recorded as adverse events. The investigator shall verify whether the diagnosis of CMT1A has been genetically confirmed for the patient.

- **Physical examination**
  This shall be performed at Visit 1 (screening), and it shall include external appearance, skin, head/neck, chest/lungs, heart, abdomen, urinary/reproductive system, limbs, musculoskeletal system, nervous system, lymph nodes, and other items. All abnormal findings shall be recorded in the case report form, and the clinical significance of each finding shall be assessed.

- **Body measurement**
  This shall include height, weight, and BMI (obtained from height in meters and weight in kg that has been rounded up to 1 decimal place from 2 decimal places).

- **Virus serology test**

At Visit 1 (screening), serology tests for the following infective viral diseases shall be performed: HIV, HBsAg, Anti-HBs, Anti-HCV

- **Complete blood cell count**

  This shall be performed at Visit 1 and throughout the clinical study every time a subject makes a visit (shall be performed prior to administering the investigational product at Visits 2, 3, 4, and 5). However, if there is a subject with abnormal findings in the test at Visit 3 (Day 14), a complete blood cell count and a general blood chemistry test may be performed at Day 30 depending on the decision of the principal investigator. The complete blood cell count items are as follows:

  WBC, RBC, Hb, Hct, MCV, MCH, MCHC, PLT, ESR, MPV, differential count of WBC (Band neutrophil, Segmented neutrophil, Eosinophil, Basophil, Lymphocyte, Monocyte)

- **General blood chemistry test**

  This shall be performed every time a subject makes a visit (shall be performed prior to administering the investigational product at Visits 2, 3, 4, and 5). However, if there is a subject with abnormal findings in the test at Visit 3 (Day 14), a complete blood cell count and a general blood chemistry test may be performed at Day 30 depending on the decision of the principal investigator. Since a blood glucose test is included, the subject shall maintain an 8-hour fasting state prior to the test. The general blood chemistry test items are as follows:

  total protein, albumin, globulin, A/G ratio, cholesterol, total bilirubin, AST, ALT, fasting glucose, BUN, creatinine, estimated GFR, Ca^{2+}, phosphate, Na^{+}, K^{+}, Cl^{-}, CRP, triglyceride, HDL-cholesterol, LDL-cholesterol

- **Chest X-ray (PA)**

  At Visit 1 (screening), chest PA X-ray shall be performed to verify whether active tuberculosis is present. The results within 1 month (30 days) before Visit 1 (screening) may be used.

- **Urinalysis with microscopy**

  This shall be performed at Visit 1 and throughout the clinical study every time a subject makes a visit (shall be performed prior to administering the investigational product at Visits 2, 3, 4, and 5). However, if there is a subject with abnormal findings in the test at Visit 3 (Day 14), urinalysis with microscopy may be performed at Day 30 depending on the decision of the principal investigator.
  The urinalysis items are as follows:

  Color, turbidity, specific gravity, pH, albumin, glucose, ketones, bilirubin, blood, urobilinogen,

nitrite, leukocyte esterase, microscopy (RBC, WBC, casts)

- **Urine HCG test for female subjects**

  At Visit 1, female subjects of childbearing potential shall undergo a urine pregnancy test (urine β-HCG, beta-human chorionic gonadotropin). At Visit 1 (screening), urine pregnancy tests shall be performed in females of childbearing potential (from post-menarche females to females ≥ 50 years of age within 1 year of menopause, or from post-menarche females to females < 50 years of age within 2 years of menopause). A serum pregnancy test may serve as a substitute if collection of urine fails. However, patients with surgical menopause (hysterectomy, bilateral oophorectomy, etc.) or who underwent sterilization surgery (bilateral tubal ligation, bilateral tubectomy) may be excluded. Menopause refers to the state after 1 year of amenorrhea. The test result must be negative, and effective methods of contraception shall be documented. The samples shall be immediately discarded when the test is completed. Acceptable methods of contraception are as follows:

  - Drug: Oral contraceptives, skin patches, or progestin formulations (implants or injections)
  - Barrier method: Condoms, diaphragms, intrauterine devices (IUDs), vaginal suppositories
  - Abstinence: Complete abstinence (However, periodic abstinence (e.g., calendar method, ovulation method, and sympto-thermal method) and self-restraint are not considered as acceptable methods of contraception.)

- **Electrocardiogram**

  A 12-lead electrocardiogram shall be performed at Visit 1 (screening). If there are results from a test performed at the same institution within 4 weeks before Visit 1 (screening), they may serve as a substitute.

- **Vital signs**

  Vital signs including blood pressure (measurement shall be taken with the subject in a sitting position after resting for at least 10 minutes prior to measurement), temperature, heart rate per minute, and respiratory rate per minute shall be measured. The measurement results shall be recorded in the subjects' case report forms. This shall be performed at Visit 1 (screening) and throughout the clinical study every time a subject makes a visit (on the drug administration days consisting of Visits 2, 3, 4, and 5, it shall be performed both prior to administration and 2 ± 1 hours after administration). However, if there is a subject with abnormal findings in the test at Visit 3 (Day 14), a follow-up test may be performed at Day 30 depending on the decision of the principal investigator.

- **Concomitant medications survey**

  The brand names and ingredient names of concomitant medications shall be surveyed at every visit and recorded in the case report form. All medications and treatments administered within 6 months before Visit 1 (screening) shall be surveyed. Previous drugs and previous treatments shall be defined as all previously collected medications and treatments before Visit 2 (first

administration of investigational product). Concomitant medications and treatments shall refer to all medications that have been administered at least once starting from Visit 2 (first administration of investigational product) and throughout the clinical study. The categories of collected medications and treatments shall follow this definition. The following shall also be surveyed for each medication:

- Brand name
- Indications for medication administration (reason for administration)
- Dose/strength, administration route, number of administrations
- Start and discontinuation dates of administration (whether to continue when the clinical study is terminated)

- **Neurologic exam**

This shall be performed for CMTNS-v2 measurement, and the investigator shall perform the neurologic exam (motor system: muscle strength; sensory system: nociception, pallesthesia) on a subject as follows:

Table 9. Neurologic exam items and procedures for CMTNS-v2 measurement

Test Item	Procedure
Muscle strength	Reduced muscle strength is referred to as weakness or paresis, and the loss of strength is referred to as paralysis.<br>To measure the muscle strength of limbs, the flexion (C5-6) and extension of wrist joint, extension of wrist (C6-8), grasping test (C7-T1), abduction of finger (C8-T1, ulnar nerve), and opposition of thumb (C8-T1, median nerve) shall be tested in the upper limbs. For the strength of intrinsic hand muscles, only the two muscles of the abductor pollicis brevis (APB) and the first dorsal interosseous (FDI) shall be evaluated, and the stronger of the two shall be scored.<br>In the lower limbs, the flexion (L2-L4), adduction (L2-L4), and extension (S1) of hip joint, the extension (L2-L4) and flexion (L4-S2) of knee, as well as the dorsiflexion (L4-5) and plantar flexion (S1) of ankle shall be tested.<br><br>The results of performing a muscle strength test of the limbs in a subject shall be indicated using the Medical Research Council (MRC) grade*.<br>* MRC grade (Motor power)<br>0: Complete paralysis<br>I: Flicker of contraction possible<br>II: Movement possible if gravity eliminated<br>III: Movement against gravity but not resistance<br>IV: Movement possible against some resistance<br>V: Power normal (it is not normally possible to overcome a normal adult's power)
Nociception	Stimulation shall be applied by alternately using tools with a dull end and a sharp end, and it shall be verified whether these can be distinguished. When comparing the left and right sides of the body, stimulation shall be applied to the same sites. Then, the subject shall be asked whether the sensations are the same.
Pallesthesia	Vibration shall be applied to a low-pitched tuning fork (a Rydel-Seiffer tuning fork shall be used), and this shall be placed on the distal joints of the hands and feet to verify that the subject feels the vibrating sensation. Pallesthesia is the first sensation lost in peripheral nerve disorders.

- **CMTNS-v2**

  Charcot-Marie-Tooth Neuropathy Score-version 2 (CMTNS-v2) is a measurement tool for evaluating the severity of disease.[66] Measurements shall be taken for 9 items which include 3 items for disease symptoms, 4 items for signs, and 2 items for neurophysiological testing. The severity of disease shall be classified according to scores as mild (≤10), moderate (11 to 20), and severe (>20). This clinical study shall target mild to moderate patients. This shall be performed at Visit 1 (screening), Visit 2 (Day 0), and Visit 7 (termination visit, Day 270) (shall be performed prior to administration at Visit 2 [Day 0]). See "Appendix 4. CMTNS-v2" for details.

- **Use of assistive devices and types**

  The assistive devices used by the subjects shall be surveyed at Visit 1 (screening) and at Visit 7 (termination visit, Day 270). Specialized shoes, braces for lower limbs, crutches, canes, walkers, wheelchairs, etc., fall under these devices. Even if new additional assistive devices are used during the clinical study, they shall not be included in adverse events.

- **Confirmation of inclusion/exclusion criteria**

- **Scheduled MRI**

  The muscles of lower limbs shall be imaged, and the fatty infiltration level of the leg muscles injected with the investigational product shall be measured and evaluated as fat content value (%) at one level for each muscle.[67]

  The test days shall be at Visit 2 (Day 0) and Visit 7 (termination visit, Day 270) (shall be performed prior to administration at Visit 2 [Day 0]).

6.1.1.1 Screening Failure

Subjects whose screening test results fail to satisfy the inclusion/exclusion criteria of this clinical study may not participate in the clinical study. The reasons for screening failure of these subjects who are ineligible to participate in the clinical study shall be recorded in the screening log. If screening fails, screening continues until the target number is met.

6.1.2 Visit 2 (Administration 1, Day 0)

6.1.2.1 Before Administration of Investigational Product

The following shall be performed prior to administering the investigational product.

- **Hospitalization (if applicable)**

  Subjects shall be hospitalized on the day before administration of the investigational product and their vital signs shall be measured. If they are not hospitalized, vital signs may be omitted, and the hospitalization day in the case report form shall be recorded the same as the test day prior to administration.

- **Final confirmation of inclusion/exclusion criteria**

- **Assignment of allocation numbers* when inclusion/exclusion criteria are satisfied**

  *: See section 5.3.4 Assignment of Subject Numbers

- **Body measurement (weight measurement)**

  A subject's weight shall be measured at Visit 1 and throughout the clinical study every time a subject makes a visit (shall be performed prior to administering the investigational product at Visits 2, 3, 4, and 5).

- **Complete blood cell count**

  This shall be performed at Visit 1 and throughout the clinical study every time a subject makes a visit (shall be performed prior to administering the investigational product at Visits 2, 3, 4, and 5). However, if there is a subject with abnormal findings in the test at Visit 3 (Day 14), a complete blood cell count and a general blood chemistry test may be performed at Day 30 depending on the decision of the principal investigator. The complete blood cell count items are as follows:

  WBC, RBC, Hb, Hct, MCV, MCH, MCHC, PLT, ESR, MPV, differential count of WBC (Band neutrophil, Segmented neutrophil, Eosinophil, Basophil, Lymphocyte, Monocyte)

- **General blood chemistry test**

  This shall be performed at Visit 1 and throughout the clinical study every time a subject makes a visit (shall be performed prior to administering the investigational product at Visits 2, 3, 4, and 5). However, if there is a subject with abnormal findings in the test at Visit 3 (Day 14), a complete blood cell count and a general blood chemistry test may be performed at Day 30 depending on the decision of the principal investigator. Since a blood glucose test is included, the subject shall maintain an 8-hour fasting state prior to the test. The general blood chemistry test items are as follows:

total protein, albumin, globulin, A/G ratio, cholesterol, total bilirubin, AST, ALT, fasting glucose, BUN, creatinine, estimated GFR, Ca^{2+}, phosphate, Na^{+}, K^{+}, Cl^{-}, CRP, triglyceride, HDL-cholesterol, LDL-cholesterol

- **Sample collection for retrospective biomarker study**
  The serum of subjects shall be collected and stored frozen at -70°C. All blood samples are stored with unique numbers for retrospective biomarker study after all personal information is deleted.

- **Urinalysis with microscopy**
  This shall be performed at Visit 1 and throughout the clinical study every time a subject makes a visit (shall be performed prior to administering the investigational product at Visits 2, 3, 4, and 5). However, if there is a subject with abnormal findings in the test at Visit 3 (Day 14), urinalysis with microscopy may be performed at Day 30 depending on the decision of the principal investigator.
  The urinalysis items are as follows:

  Color, turbidity, specific gravity, pH, albumin, glucose, ketones, bilirubin, blood, urobilinogen, nitrite, leukocyte esterase, microscopy (RBC, WBC, casts)

- **Vital signs**
  Vital signs including blood pressure (measurement shall be taken with the subject in a sitting position after resting for at least 10 minutes prior to measurement), temperature, heart rate per minute, and respiratory rate per minute shall be measured. The measurement results shall be recorded in the subjects' case report forms. This shall be performed at Visit 1 (screening) and throughout the clinical study every time a subject makes a visit (on the drug administration days consisting of Visits 2, 3, 4, and 5, it shall be performed both prior to administration and 2 ± 1 hours after administration). However, if there is a subject with abnormal findings in the test at Visit 3 (Day 14), a follow-up test may be performed at Day 30 depending on the decision of the principal investigator.

- **Concomitant medications survey**
  The brand names and ingredient names of concomitant medications shall be surveyed at every visit and recorded in the case report form. All medications and treatments administered within 6 months before Visit 1 (screening) shall be surveyed. Previous medications and previous treatments shall be defined as all previously collected medications and treatments before Visit 2 (first administration of investigational product). Concomitant medications and treatments shall refer to all medications that have been administered at least once starting from Visit 2 (first administration of investigational product) and throughout the clinical study. The categories of collected medications and treatments shall follow this definition. The following shall also be surveyed for each medication:
  - Brand name

- Indications for medication administration (reason for administration)
- Dose/strength, administration route, number of administrations
- Start and discontinuation dates of administration (whether to continue when the clinical study is terminated)

- **Neurologic exam**

This shall be performed for CMTNS-v2 measurement, and the investigator shall perform the neurologic exam (motor system: muscle strength; sensory system: nociception, pallesthesia) on a subject as follows:

Table 10. Neurologic exam items and procedures for CMTNS-v2 measurement

Test Item	Procedure
Muscle strength	Reduced muscle strength is referred to as weakness or paresis, and the loss of strength is referred to as paralysis.<br>To measure the muscle strength of limbs, the flexion (C5-6) and extension of wrist joint, extension of wrist (C6-8), grasping test (C7-T1), abduction of finger (C8-T1, ulnar nerve), and opposition of thumb (C8-T1, median nerve) shall be tested in the upper limbs. For the strength of intrinsic hand muscles, only the two muscles of the abductor pollicis brevis (APB) and the first dorsal interosseous (FDI) shall be evaluated, and the stronger of the two shall be scored.<br>In the lower limbs, the flexion (L2-L4), adduction (L2-L4), and extension (S1) of hip joint, the extension (L2-L4) and flexion (L4-S2) of knee, as well as the dorsiflexion (L4-5) and plantar flexion (S1) of ankle shall be tested.<br><br>The results of performing a muscle strength test of the limbs in a subject shall be indicated using the Medical Research Council (MRC) grade*.<br>* MRC grade (Motor power)<br>0: Complete paralysis<br>I: Flicker of contraction possible<br>II: Movement possible if gravity eliminated<br>III: Movement against gravity but not resistance<br>IV: Movement possible against some resistance<br>V: Power normal (it is not normally possible to overcome a normal adult's power)
Nociception	Stimulation shall be applied by alternately using tools with a dull end and a sharp end, and it shall be verified whether these can be distinguished. When comparing the left and right sides of the body, stimulation shall be applied to the same sites. Then, the subject shall be asked whether the sensations are the same.
Pallesthesia	Vibration shall be applied to a low-pitched tuning fork (a Rydel-Seiffer tuning fork shall be used), and this shall be placed on the distal joints of the hands and feet to verify that the subject feels the vibrating sensation. Pallesthesia is the first sensation lost in peripheral nerve disorders.

- **CMTNS-v2**

Charcot-Marie-Tooth Neuropathy Score-version 2 (CMTNS-v2) is a measurement tool for

evaluating the severity of disease.[66] Measurements shall be taken for 9 items which include 3 items for disease symptoms, 4 items for signs, and 2 items for neurophysiological testing. The severity of disease shall be classified according to scores as mild (≤10), moderate (11 to 20), and severe (>20). This clinical study shall target mild to moderate patients. This shall be performed at Visit 1 (screening), Visit 2 (Day 0), and Visit 7 (termination visit, Day 270) (shall be performed prior to administration at Visit 2 [Day 0]). See "Appendix 4. CMTNS-v2" for details.

- **Anti-HGF Ab test**

  The antibody test for hepatocyte growth factor shall be performed at Visit 2 (Day 0) and Visit 7 (termination visit, Day 270) (shall be performed prior to administration at Visit 2 [Day 0]). The serum of subjects shall be collected and stored frozen at -70°C. When the collection of blood from all subjects is completed, the samples shall be sent to the central laboratory all at once for analysis.

- **MRI leg**

  The muscles of lower limbs shall be imaged, and the fatty infiltration level of the leg muscles injected with the investigational product shall be measured and evaluated as fat content value (%) at one level for each muscle.[67]

  The test days shall be at Visit 2 (Day 0) and Visit 7 (termination visit, Day 270) (shall be performed prior to administration at Visit 2 [Day 0]).

- **FDS**

  The functional disability scale (FDS) assesses a patient from 0 to 8 points as shown below depending on the patient's mobility.[68] The assessment days shall be at Visit 2 (Day 0), Visit 4 (Day 90), Visit 6 (Day 180), and Visit 7 (termination visit, Day 270) (assessment shall be performed prior to administration at Visit 2 [Day 0] and Visit 4 [Day 90]).

  0=normal;
  1=cramps and fatigability;
  2=inability to run;
  3=possible unaided;
  4=with cane;
  5=with crutch;
  6=with walker;
  7=wheelchair;
  8=bedridden.

- **ONLS leg scale**

  The overall neuropathy limitation scale (ONLS) is a tool for measuring the activity level of patients with peripheral neuropathy.[69] It is scored by separately categorizing arms and legs. Measurement shall be performed only on legs in this clinical study. The measurement day shall

be at Visit 2 (Day 0), Visit 4 (Day 90), Visit 6 (Day 180), and Visit 7 (termination visit, Day 270) (measurement shall be performed prior to administration at Visit 2 [Day 0] and Visit 4 [Day 90]). See "Appendix 5. ONLS Leg scale" for details.

- **10MWT**

  This is a test that measures the time required for a subject to walk 10 meters.[70] A subject shall be made to walk at a desired speed while wearing shoes. The subject shall be allowed to use an assistive device that the subject normally uses, if any. The subject shall be made to walk a corridor that is 14 meters long, and the time taken to walk 10 meters shall be measured with a stopwatch provided by the sponsor (time taken to pass 10 meters excluding 2 meters each for the starting and ending portions). The assessment day shall be at Visit 2 (Day 0), Visit 4 (Day 90), Visit 6 (Day 180), and Visit 7 (termination visit, Day 270) (assessment shall be performed prior to administration at Visit 2 [Day 0] and Visit 4 [Day 90]).

  The starting point at 2 meters, the point for ending measurement at 12 meters, and the point for end of walking at 14 meters shall be marked in advance along the corridor.

  See "Appendix 6. 10MWT (10-meter walk test)" for details.

- **Electroneurography (CMAP, SNAP, NCV)**

  The test days shall be at Visit 2 (Day 0) and Visit 7 (termination visit, Day 270) (shall be performed prior to administration at Visit 2 [Day 0]).

  See "Appendix 7. Nerve Conduction Study (NCS)" for details.

6.1.2.2 Administration of Investigational Product (baseline, Day 0)

The investigator shall administer the investigational product (VM202) on the following sites of a subject's both lower limbs. The number of injections depending on the administration site of VM202 shall be 56 intramuscular injections in total with 28 injections for 3 sites on the left and right lower limbs, respectively. See "Appendix 3. Administration Method of Investigational Product" for details.

- Peroneus longus muscle – 6 injections for left peroneus longus muscle and 6 injections for the right peroneus longus muscle
- Gastrocnemius muscle – 12 injections for the left gastrocnemius muscle and 12 injections for the right gastrocnemius muscle
- Tibialis anterior muscle – 10 injections for the left tibialis anterior muscle and 10 injections for the right tibialis anterior muscle

6.1.2.3 After Administration of Investigational Product (baseline, Day 0)

The following shall be performed after administration of the investigational product.

- **Vital signs**

Vital signs including blood pressure (measurement shall be taken with the subject in a sitting position after resting for at least 10 minutes prior to measurement), temperature, heart rate per minute, and respiratory rate per minute shall be measured. The measurement results shall be recorded in the subjects' case report forms. This shall be performed at Visit 1 (screening) and throughout the clinical study every time a subject makes a visit (on the drug administration days consisting of Visits 2, 3, 4, and 5, it shall be performed both prior to administration and 2 ± 1 hours after administration). However, if there is a subject with abnormal findings in the test at Visit 3 (Day 14), a follow-up test may be performed at Day 30 depending on the decision of the principal investigator.

- **Adverse event assessment**

  Localized adverse events shall be checked at 2 ± 1 hours after administering the investigational product and on the day after administration.

- **Scheduled MRI and electroneurography**

  This shall be scheduled to be performed on the same day as Visit 7 (Day 270) prior to discharge.

6.1.3 Visit 3 (Second Administration, Day 14 ± 5)

6.1.3.1 Before Administration of Investigational Product

The following shall be performed prior to administering the investigational product.

- **Adverse event assessment**

  Information on newly developed or worsened adverse events, or adverse events that disappeared since the last visit shall be collected.

- **Body measurement (weight measurement)**

  A subject's weight shall be measured at Visit 1 and throughout the clinical study every time a subject makes a visit (shall be performed prior to administering the investigational product at Visits 2, 3, 4, and 5).

- **Complete blood cell count**

  This shall be performed at Visit 1 and throughout the clinical study every time a subject makes a visit (shall be performed prior to administering the investigational product at Visits 2, 3, 4, and 5). However, if there is a subject with abnormal findings in the test at Visit 3 (Day 14), a complete blood cell count and a general blood chemistry test may be performed at Day 30 depending on the decision of the principal investigator. The complete blood cell count items are as follows:

  WBC, RBC, Hb, Hct, MCV, MCH, MCHC, PLT, ESR, MPV, differential count of WBC (Band neutrophil,

Segmented neutrophil, Eosinophil, Basophil, Lymphocyte, Monocyte)

- **General blood chemistry test**
  This shall be performed at Visit 1 and throughout the clinical study every time a subject makes a visit (shall be performed prior to administering the investigational product at Visits 2, 3, 4, and 5). However, if there is a subject with abnormal findings in the test at Visit 3 (Day 14), a complete blood cell count and a general blood chemistry test may be performed at Day 30 depending on the decision of the principal investigator. Since a blood glucose test is included, the subject shall maintain an 8-hour fasting state prior to the test. The general blood chemistry test items are as follows:

  total protein, albumin, globulin, A/G ratio, cholesterol, total bilirubin, AST, ALT, fasting glucose, BUN, creatinine, estimated GFR, Ca^{2+}, phosphate, Na^{+}, K^{+}, Cl^{-}, CRP, triglyceride, HDL- cholesterol, LDL-cholesterol

- **Urinalysis with microscopy**
  This shall be performed at Visit 1 and throughout the clinical study every time a subject makes a visit (shall be performed prior to administering the investigational product at Visits 2, 3, 4, and 5). However, if there is a subject with abnormal findings in the test at Visit 3 (Day 14), urinalysis with microscopy may be performed at Day 30 depending on the decision of the principal investigator.
  The urinalysis items are as follows:

  Color, turbidity, specific gravity, pH, albumin, glucose, ketones, bilirubin, blood, urobilinogen, nitrite, leukocyte esterase, microscopy (RBC, WBC, casts)

- **Vital signs**
  Vital signs including blood pressure (measurement shall be taken with the subject in a sitting position after resting for at least 10 minutes prior to measurement), temperature, heart rate per minute, and respiratory rate per minute shall be measured. The measurement results shall be recorded in the subjects' case report forms. This shall be performed at Visit 1 (screening) and throughout the clinical study every time a subject makes a visit (on the drug administration days consisting of Visits 2, 3, 4, and 5, it shall be performed both prior to administration and 2 ± 1 hours after administration). However, if there is a subject with abnormal findings in the test at Visit 3 (Day 14), a follow-up test may be performed at Day 30 depending on the decision of the principal investigator.

- **Concomitant medications survey**
  The brand names and ingredient names of concomitant medications shall be surveyed at every visit and recorded in the case report form. All medications and treatments administered within 6 months before Visit 1 (screening) shall be surveyed. Previous medications and previous

treatments shall be defined as all previously collected medications and treatments before Visit 2 (first administration of investigational product). Concomitant medications and treatments shall refer to all medications that have been administered at least once starting from Visit 2 (first administration of investigational product) and throughout the clinical study. The categories of collected medications and treatments shall follow this definition. The following shall also be surveyed for each medication:

- Brand name
- Indications for medication administration (reason for administration)
- Dose/strength, administration route, number of administrations
- Start and discontinuation dates of administration (whether to continue when the clinical study is terminated)

- **Neurologic exam**

Along with the assessments for the patient's sensation and motor symptoms, the following neurologic exam (motor system: muscle strength; sensory system: nociception, pallesthesia) shall be performed.

Table 11. Neurologic exam items and procedures

Test Item	Procedure
Muscle strength	Reduced muscle strength is referred to as weakness or paresis, and the loss of strength is referred to as paralysis.<br>To measure the muscle strength of limbs, the flexion (C5-6) and extension of wrist joint, extension of wrist (C6-8), grasping test (C7-T1), abduction of finger (C8-T1, ulnar nerve), and opposition of thumb (C8-T1, median nerve) shall be tested in the upper limbs. For the strength of intrinsic hand muscles, only the two muscles of the abductor pollicis brevis (APB) and the first dorsal interosseous (FDI) shall be evaluated, and the stronger of the two shall be scored.<br>In the lower limbs, the flexion (L2-L4), adduction (L2-L4), and extension (S1) of hip joint, the extension (L2-L4) and flexion (L4-S2) of knee, as well as the dorsiflexion (L4-5) and plantar flexion (S1) of ankle shall be tested.<br><br>The results of performing a muscle strength test of the limbs in a subject shall be indicated using the Medical Research Council (MRC) grade*.<br>* MRC grade (Motor power)<br>0: Complete paralysis<br>I: Flicker of contraction possible<br>II: Movement possible if gravity eliminated<br>III: Movement against gravity but not resistance<br>IV: Movement possible against some resistance<br>V: Power normal (it is not normally possible to overcome a normal adult's power)
Nociception	Stimulation shall be applied by alternately using tools with a dull end and a sharp end, and it shall be verified whether these can be distinguished. When comparing the left and right sides of the body, stimulation shall be applied to the same sites. Then, the subject shall be asked whether the sensations are the same.
Pallesthesia	Vibration shall be applied to a low-pitched tuning fork (a Rydel-Seiffer tuning fork shall be used), and this shall be placed on the distal joints of the hands and feet to verify that the subject feels the vibrating sensation. Pallesthesia is

	the first sensation lost in peripheral nerve disorders.

6.1.3.2 Administration of Investigational Product

The investigator shall administer the investigational product (VM202) on the following sites of a subject's both lower limbs. The number of injections depending on the administration site of VM202 shall be 56 intramuscular injections in total with 28 injections for 3 sites on the left and right lower limbs, respectively. See "Appendix 3. Administration Method of Investigational Product" for details.

- Peroneus longus muscle – 6 injections for left peroneus longus muscle and 6 injections for the right peroneus longus muscle
- Gastrocnemius muscle – 12 injections for the left gastrocnemius muscle and 12 injections for the right gastrocnemius muscle
- Tibialis anterior muscle – 10 injections for the left tibialis anterior muscle and 10 injections for the right tibialis anterior muscle

6.1.3.3 After Administration of Investigational Product

The following shall be performed after administration of the investigational product.

- **Vital signs**

  Vital signs including blood pressure (measurement shall be taken with the subject in a sitting position after resting for at least 10 minutes prior to measurement), temperature, heart rate per minute, and respiratory rate per minute shall be measured. The measurement results shall be recorded in the subjects' case report forms. This shall be performed at Visit 1 (screening) and throughout the clinical study every time a subject makes a visit (on the drug administration days consisting of Visits 2, 3, 4, and 5, it shall be performed both prior to administration and 2 ± 1 hours after administration). However, if there is a subject with abnormal findings in the test at Visit 3 (Day 14), a follow-up test may be performed at Day 30 depending on the decision of the principal investigator.

- **Adverse event assessment**

  Localized adverse events shall be checked at 2 ± 1 hours after administering the investigational product and on the day after administration.

6.1.4 Day 30 ± 7 (shall be performed only if follow-up testing is required)

This shall be performed only for subjects who are determined by the principal investigator to take short-term follow-up testing due to abnormal findings in the

hematology test at Visit 2 (Day 14).

- **Adverse event assessment**

  Information on newly developed or worsened adverse events, or adverse events that disappeared since the last visit shall be collected.

- **Body measurement (weight measurement)**

  A subject's weight shall be measured at Visit 1 and throughout the clinical study every time a subject makes a visit (shall be performed prior to administering the investigational product at Visits 2, 3, 4, and 5).

- **Complete blood cell count**

  This shall be performed at Visit 1 and throughout the clinical study every time a subject makes a visit (shall be performed prior to administering the investigational product at Visits 2, 3, 4, and 5). However, if there is a subject with abnormal findings in the test at Visit 3 (Day 14), a complete blood cell count and a general blood chemistry test may be performed at Day 30 depending on the decision of the principal investigator. The complete blood cell count items are as follows:

  WBC, RBC, Hb, Hct, MCV, MCH, MCHC, PLT, ESR, MPV, differential count of WBC (Band neutrophil, Segmented neutrophil, Eosinophil, Basophil, Lymphocyte, Monocyte)

- **General blood chemistry test**

  This shall be performed at Visit 1 and throughout the clinical study every time a subject makes a visit (shall be performed prior to administering the investigational product at Visits 2, 3, 4, and 5). However, if there is a subject with abnormal findings in the test at Visit 3 (Day 14), a complete blood cell count and a general blood chemistry test may be performed at Day 30 depending on the decision of the principal investigator. Since a blood glucose test is included, the subject shall maintain an 8-hour fasting state prior to the test. The general blood chemistry test items are as follows:

  total protein, albumin, globulin, A/G ratio, cholesterol, total bilirubin, AST, ALT, fasting glucose, BUN, creatinine, estimated GFR, Ca^{2+}, phosphate, Na^{+}, K^{+}, Cl^{-}, CRP, triglyceride, HDL-cholesterol, LDL-cholesterol

- **Urinalysis with microscopy**

  This shall be performed at Visit 1 and throughout the clinical study every time a subject makes a visit (shall be performed prior to administering the investigational product at Visits 2, 3, 4, and 5). However, if there is a subject with abnormal findings in the test at Visit 3 (Day 14), urinalysis with microscopy may be performed at Day 30 depending on the decision of the principal investigator.

  The urinalysis items are as follows:

Color, turbidity, specific gravity, pH, albumin, glucose, ketones, bilirubin, blood, urobilinogen, nitrite, leukocyte esterase, microscopy (RBC, WBC, casts)

- **Vital signs**

  Vital signs including blood pressure (measurement shall be taken with the subject in a sitting position after resting for at least 10 minutes prior to measurement), temperature, heart rate per minute, and respiratory rate per minute shall be measured. The measurement results shall be recorded in the subjects' case report forms. This shall be performed at Visit 1 (screening) and throughout the clinical study every time a subject makes a visit (on the drug administration days consisting of Visits 2, 3, 4, and 5, it shall be performed both prior to administration and 2 ± 1 hours after administration). However, if there is a subject with abnormal findings in the test at Visit 3 (Day 14), a follow-up test may be performed at Day 30 depending on the decision of the principal investigator.

- **Concomitant medications survey**

  The brand names and ingredient names of concomitant medications shall be surveyed at every visit and recorded in the case report form. All medications and treatments administered within 6 months before Visit 1 (screening) shall be surveyed. Previous medications and previous treatments shall be defined as all previously collected medications and treatments before Visit 2 (first administration of investigational product). Concomitant medications and treatments shall refer to all medications that have been administered at least once starting from Visit 2 (first administration of investigational product) and throughout the clinical study. The categories of collected medications and treatments shall follow this definition. The following shall also be surveyed for each medication:

  - Brand name
  - Indications for medication administration (reason for administration)
  - Dose/strength, administration route, number of administrations
  - Start and discontinuation dates of administration (whether to continue when the clinical study is terminated)

- **Neurologic exam**

  Along with the assessments for the patient's sensation and motor symptoms, the following neurologic exam (motor system: muscle strength; sensory system: nociception, pallesthesia) shall be performed.

Table 12. Neurologic exam items and procedures

Test Item	Procedure
Muscle strength	Reduced muscle strength is referred to as weakness or paresis, and the loss of strength is referred to as paralysis.<br>To measure the muscle strength of limbs, the flexion (C5-6) and extension of wrist joint, extension of wrist (C6-8), grasping test (C7-T1), abduction of finger

	(C8-T1, ulnar nerve), and opposition of thumb (C8-T1, median nerve) shall be tested in the upper limbs. For the strength of intrinsic hand muscles, only the two muscles of the abductor pollicis brevis (APB) and the first dorsal interosseous (FDI) shall be evaluated, and the stronger of the two shall be scored.<br>In the lower limbs, the flexion (L2-L4), adduction (L2-L4), and extension (S1) of hip joint, the extension (L2-L4) and flexion (L4-S2) of knee, as well as the dorsiflexion (L4-5) and plantar flexion (S1) of ankle shall be tested.<br><br>The results of performing a muscle strength test of the limbs in a subject shall be indicated using the Medical Research Council (MRC) grade*.<br>* MRC grade (Motor power)<br>0: Complete paralysis<br>I: Flicker of contraction possible<br>II: Movement possible if gravity eliminated<br>III: Movement against gravity but not resistance<br>IV: Movement possible against some resistance<br>V: Power normal (it is not normally possible to overcome a normal adult's power)
Nociception	Stimulation shall be applied by alternately using tools with a dull end and a sharp end, and it shall be verified whether these can be distinguished. When comparing the left and right sides of the body, stimulation shall be applied to the same sites. Then, the subject shall be asked whether the sensations are the same.
Pallesthesia	Vibration shall be applied to a low-pitched tuning fork (a Rydel-Seiffer tuning fork shall be used), and this shall be placed on the distal joints of the hands and feet to verify that the subject feels the vibrating sensation. Pallesthesia is the first sensation lost in peripheral nerve disorders.

6.1.5 Visit 4 (Third Administration, Day 90 ± 7)

6.1.5.1 Before Administration of Investigational Product

The following shall be performed prior to administering the investigational product.

- **Adverse event assessment**

  Information on newly developed or worsened adverse events, or adverse events that disappeared since the last visit shall be collected.

- **Body measurement (weight measurement)**

  A subject's weight shall be measured at Visit 1 and throughout the clinical study every time a subject makes a visit (shall be performed prior to administering the investigational product at Visits 2, 3, 4, and 5).

- **Complete blood cell count**

  This shall be performed at Visit 1 and throughout the clinical study every time a subject makes a visit (shall be performed prior to administering the investigational product at Visits 2, 3, 4, and 5). However, if there is a subject with abnormal findings in the test at Visit 3

(Day 14), a complete blood cell count and a general blood chemistry test may be performed at Day 30 depending on the decision of the principal investigator.
The complete blood cell count items are as follows:

WBC, RBC, Hb, Hct, MCV, MCH, MCHC, PLT, ESR, MPV, differential count of WBC (Band neutrophil, Segmented neutrophil, Eosinophil, Basophil, Lymphocyte, Monocyte)

- **General blood chemistry test**

  This shall be performed at Visit 1 and throughout the clinical study every time a subject makes a visit (shall be performed prior to administering the investigational product at Visits 2, 3, 4, and 5). However, if there is a subject with abnormal findings in the test at Visit 3 (Day 14), a complete blood cell count and a general blood chemistry test may be performed at Day 30 depending on the decision of the principal investigator. Since a blood glucose test is included, the subject shall maintain an 8-hour fasting state prior to the test.
  The general blood chemistry test items are as follows:

  total protein, albumin, globulin, A/G ratio, cholesterol, total bilirubin, AST, ALT, fasting glucose, BUN, creatinine, estimated GFR, Ca^{2+}, phosphate, Na^{+}, K^{+}, Cl^{-}, CRP, triglyceride, HDL-cholesterol, LDL-cholesterol

- **Sample collection for retrospective biomarker study**

  The serum of subjects shall be collected and stored frozen at -70°C. All blood samples are stored with unique numbers for retrospective biomarker study after all personal information is deleted.

- **Urinalysis with microscopy**

  This shall be performed at Visit 1 and throughout the clinical study every time a subject makes a visit (shall be performed prior to administering the investigational product at Visits 2, 3, 4, and 5). However, if there is a subject with abnormal findings in the test at Visit 3 (Day 14), urinalysis may be performed at Day 30 depending on the decision of the principal investigator.
  The urinalysis items are as follows:

  Color, turbidity, specific gravity, pH, albumin, glucose, ketones, bilirubin, blood, urobilinogen, nitrite, leukocyte esterase, microscopy (RBC, WBC, casts)

- **Vital signs**

  Vital signs including blood pressure (measurement shall be taken with the subject in a sitting position after resting for at least 10 minutes prior to measurement), temperature, heart rate per minute, and respiratory rate per minute shall be measured. The measurement results shall be recorded in the subjects' case report forms. This shall be performed at Visit 1 (screening) and throughout the clinical study every time a subject makes a visit (on the drug

administration days consisting of Visits 2, 3, 4, and 5, it shall be performed both prior to administration and 2 ± 1 hours after administration). However, if there is a subject with abnormal findings in the test at Visit 3 (Day 14), a follow-up test may be performed at Day 30 depending on the decision of the principal investigator.

- **Concomitant medications survey**

  The brand names and ingredient names of concomitant medications shall be surveyed at every visit and recorded in the case report form. All medications and treatments administered within 6 months before Visit 1 (screening) shall be surveyed. Previous medications and previous treatments shall be defined as all previously collected medications and treatments before Visit 2 (first administration of investigational product). Concomitant medications and treatments shall refer to all medications that have been administered at least once starting from Visit 2 (first administration of investigational product) and throughout the clinical study. The categories of collected medications and treatments shall follow this definition. The following shall also be surveyed for each medication:

  - Brand name
  - Indications for medication administration (reason for administration)
  - Dose/strength, administration route, number of administrations
  - Start and discontinuation dates of administration (whether to continue when the clinical study is terminated)

- **FDS**

  The functional disability scale (FDS) assesses a patient from 0 to 8 points as shown below depending on the patient's mobility.[68] The assessment days shall be at Visit 2 (Day 0), Visit 4 (Day 90), Visit 6 (Day 180), and Visit 7 (termination visit, Day 270) (assessment shall be performed prior to administration at Visit 2 [Day 0] and Visit 4 [Day 90]).

  0=normal;
  1=cramps and fatigability;
  2=inability to run;
  3=possible unaided;
  4=with cane;
  5=with crutch;
  6=with walker;
  7=wheelchair;
  8=bedridden.

- **ONLS leg scale**

  The overall neuropathy limitation scale (ONLS) is a tool for measuring the activity level of patients with peripheral neuropathy.[69] It is scored by separately categorizing arms and legs. Measurement shall be performed only on legs in this clinical study. The measurement day shall be at Visit 2 (Day 0), Visit 4 (Day 90), Visit 6 (Day 180), and Visit 7 (termination visit, Day 270)

(measurement shall be performed prior to administration at Visit 2 [Day 0] and Visit 4 [Day 90]). See "Appendix 5. ONLS Leg scale" for details.

- **10MWT**

This is a test that measures the time required for a subject to walk 10 meters.[70] A subject shall be made to walk at a desired speed while wearing shoes. The subject shall be allowed to use an assistive device that the subject normally uses, if any. The subject shall be made to walk a corridor that is 14 meters long, and the time taken to walk 10 meters shall be measured with a stopwatch provided by the sponsor (time taken to pass 10 meters excluding 2 meters each for the starting and ending portions). The assessment day shall be at Visit 2 (Day 0), Visit 4 (Day 90), Visit 6 (Day 180), and Visit 7 (termination visit, Day 270) (assessment shall be performed prior to administration at Visit 2 [Day 0] and Visit 4 [Day 90]).
The starting point at 2 meters, the point for ending measurement at 12 meters, and the point for end of walking at 14 meters shall be marked in advance along the corridor.
See "Appendix 6. 10MWT (10-meter walk test)" for details.

- **Neurologic exam**

Along with the assessments for the patient's sensation and motor symptoms, the following neurologic exam (motor system: muscle strength; sensory system: nociception, pallesthesia) shall be performed.

Table 13. Neurologic exam items and procedures

Test Item	Procedure
Muscle strength	Reduced muscle strength is referred to as weakness or paresis, and the loss of strength is referred to as paralysis.<br>To measure the muscle strength of limbs, the flexion (C5-6) and extension of wrist joint, extension of wrist (C6-8), grasping test (C7-T1), abduction of finger (C8-T1, ulnar nerve), and opposition of thumb (C8-T1, median nerve) shall be tested in the upper limbs. For the strength of intrinsic hand muscles, only the two muscles of the abductor pollicis brevis (APB) and the first dorsal interosseous (FDI) shall be evaluated, and the stronger of the two shall be scored.<br>In the lower limbs, the flexion (L2-L4), adduction (L2-L4), and extension (S1) of hip joint, the extension (L2-L4) and flexion (L4-S2) of knee, as well as the dorsiflexion (L4-5) and plantar flexion (S1) of ankle shall be tested.<br><br>The results of performing a muscle strength test of the limbs in a subject shall be indicated using the Medical Research Council (MRC) grade*.<br>* MRC grade (Motor power)<br>0: Complete paralysis<br>I: Flicker of contraction possible<br>II: Movement possible if gravity eliminated<br>III: Movement against gravity but not resistance<br>IV: Movement possible against some resistance<br>V: Power normal (it is not normally possible to overcome a normal adult's power)
Nociception	Stimulation shall be applied by alternately using tools with a dull end and a sharp end, and it shall be verified whether these can be distinguished. When

	comparing the left and right sides of the body, stimulation shall be applied to the same sites. Then, the subject shall be asked whether the sensations are the same.
Pallesthesia	Vibration shall be applied to a low-pitched tuning fork (a Rydel-Seiffer tuning fork shall be used), and this shall be placed on the distal joints of the hands and feet to verify that the subject feels the vibrating sensation. Pallesthesia is the first sensation lost in peripheral nerve disorders.

6.1.5.2 Administration of Investigational Product

The investigator shall administer the investigational product (VM202) on the following sites of a subject's both lower limbs. The number of injections depending on the administration site of VM202 shall be 56 intramuscular injections in total with 28 injections for 3 sites on the left and right lower limbs, respectively. See "Appendix 3. Administration Method of Investigational Product" for details.

- Peroneus longus muscle – 6 injections for left peroneus longus muscle and 6 injections for the right peroneus longus muscle
- Gastrocnemius muscle – 12 injections for the left gastrocnemius muscle and 12 injections for the right gastrocnemius muscle
- Tibialis anterior muscle – 10 injections for the left tibialis anterior muscle and 10 injections for the right tibialis anterior muscle

6.1.5.3 After Administration of Investigational Product

The following shall be performed after administration of the investigational product.

- **Vital signs**

  Vital signs including blood pressure (measurement shall be taken with the subject in a sitting position after resting for at least 10 minutes prior to measurement), temperature, heart rate per minute, and respiratory rate per minute shall be measured. The measurement results shall be recorded in the subjects' case report forms. This shall be performed at Visit 1 (screening) and throughout the clinical study every time a subject makes a visit (on the drug administration days consisting of Visits 2, 3, 4, and 5, it shall be performed both prior to administration and 2 ± 1 hours after administration). However, if there is a subject with abnormal findings in the test at Visit 3 (Day 14), a follow-up test may be performed at Day 30 depending on the decision of the principal investigator.

- **Adverse event assessment**

  Localized adverse events shall be checked at 2 ± 1 hours after administering the investigational product and on the day after administration.

6.1.6 Visit 5 (Fourth Administration, Day 104 ± 7)

6.1.6.1 Before Administration of Investigational Product

The following shall be performed prior to administering the investigational product.

- **Adverse event assessment**
  Information on newly developed or worsened adverse events, or adverse events that disappeared since the last visit shall be collected.

- **Body measurement (weight measurement)**
  A subject's weight shall be measured at Visit 1 and throughout the clinical study every time a subject makes a visit (shall be performed prior to administering the investigational product at Visits 2, 3, 4, and 5).

- **Complete blood cell count**
  This shall be performed at Visit 1 and throughout the clinical study every time a subject makes a visit (shall be performed prior to administering the investigational product at Visits 2, 3, 4, and 5). However, if there is a subject with abnormal findings in the test at Visit 3 (Day 14), a complete blood cell count and a general blood chemistry test may be performed at Day 30 depending on the decision of the principal investigator.
  The complete blood cell count items are as follows:

  WBC, RBC, Hb, Hct, MCV, MCH, MCHC, PLT, ESR, MPV, differential count of WBC (Band neutrophil, Segmented neutrophil, Eosinophil, Basophil, Lymphocyte, Monocyte)

- **General blood chemistry test**
  This shall be performed at Visit 1 and throughout the clinical study every time a subject makes a visit (shall be performed prior to administering the investigational product at Visits 2, 3, 4, and 5). However, if there is a subject with abnormal findings in the test at Visit 3 (Day 14), a complete blood cell count and a general blood chemistry test may be performed at Day 30 depending on the decision of the principal investigator. Since a blood glucose test is included, the subject shall maintain an 8-hour fasting state prior to the test.
  The general blood chemistry test items are as follows:

  total protein, albumin, globulin, A/G ratio, cholesterol, total bilirubin, AST, ALT, fasting glucose, BUN, creatinine, estimated GFR, Ca^{2+}, phosphate, Na^{+}, K^{+}, Cl^{-}, CRP, triglyceride, HDL-cholesterol, LDL-cholesterol

- **Urinalysis with microscopy**
  This shall be performed at Visit 1 and throughout the clinical study every time a subject makes a visit (shall be performed prior to administering the investigational product at Visits

2, 3, 4, and 5). However, if there is a subject with abnormal findings in the test at Visit 3 (Day 14), urinalysis may be performed at Day 30 depending on the decision of the principal investigator. The urinalysis items are as follows:

Color, turbidity, specific gravity, pH, albumin, glucose, ketones, bilirubin, blood, urobilinogen, nitrite, leukocyte esterase, microscopy (RBC, WBC, casts)

- **Vital signs**

  Vital signs including blood pressure (measurement shall be taken with the subject in a sitting position after resting for at least 10 minutes prior to measurement), temperature, heart rate per minute, and respiratory rate per minute shall be measured. The measurement results shall be recorded in the subjects' case report forms. This shall be performed at Visit 1 (screening) and throughout the clinical study every time a subject makes a visit (on the drug administration days consisting of Visits 2, 3, 4, and 5, it shall be performed both prior to administration and 2 ± 1 hours after administration). However, if there is a subject with abnormal findings in the test at Visit 3 (Day 14), a follow-up test may be performed at Day 30 depending on the decision of the principal investigator.

- **Concomitant medications survey**

  The brand names and ingredient names of concomitant medications shall be surveyed at every visit and recorded in the case report form. All medications and treatments administered within 6 months before Visit 1 (screening) shall be surveyed. Previous medications and previous treatments shall be defined as all previously collected medications and treatments before Visit 2 (first administration of investigational product). Concomitant medications and treatments shall refer to all medications that have been administered at least once starting from Visit 2 (first administration of investigational product) and throughout the clinical study. The categories of collected medications and treatments shall follow this definition. The following shall also be surveyed for each medication:
  - Brand name
  - Indications for medication administration (reason for administration)
  - Dose/strength, administration route, number of administrations
  - Start and discontinuation dates of administration (whether to continue when the clinical study is terminated)

- **Neurologic exam**

  Along with the assessments for the patient's sensation and motor symptoms, the following neurologic exam (motor system: muscle strength; sensory system: nociception, pallesthesia) shall be performed.

Table 14. Neurologic exam items and procedures

Test Item	Procedure
Muscle	Reduced muscle strength is referred to as weakness or paresis, and the loss of

strength	strength is referred to as paralysis.<br>To measure the muscle strength of limbs, the flexion (C5-6) and extension of wrist joint, extension of wrist (C6-8), grasping test (C7-T1), abduction of finger (C8-T1, ulnar nerve), and opposition of thumb (C8-T1, median nerve) shall be tested in the upper limbs. For the strength of intrinsic hand muscles, only the two muscles of the abductor pollicis brevis (APB) and the first dorsal interosseous (FDI) shall be evaluated, and the stronger of the two shall be scored.<br>In the lower limbs, the flexion (L2-L4), adduction (L2-L4), and extension (S1) of hip joint, the extension (L2-L4) and flexion (L4-S2) of knee, as well as the dorsiflexion (L4-5) and plantar flexion (S1) of ankle shall be tested.<br><br>The results of performing a muscle strength test of the limbs in a subject shall be indicated using the Medical Research Council (MRC) grade*.<br>* MRC grade (Motor power)<br>0: Complete paralysis<br>I: Flicker of contraction possible<br>II: Movement possible if gravity eliminated<br>III: Movement against gravity but not resistance<br>IV: Movement possible against some resistance<br>V: Power normal (it is not normally possible to overcome a normal adult's power)
Nociception	Stimulation shall be applied by alternately using tools with a dull end and a sharp end, and it shall be verified whether these can be distinguished. When comparing the left and right sides of the body, stimulation shall be applied to the same sites. Then, the subject shall be asked whether the sensations are the same.
Pallesthesia	Vibration shall be applied to a low-pitched tuning fork (a Rydel-Seiffer tuning fork shall be used), and this shall be placed on the distal joints of the hands and feet to verify that the subject feels the vibrating sensation. Pallesthesia is the first sensation lost in peripheral nerve disorders.

6.1.6.2 Administration of Investigational Product

The investigator shall administer the investigational product (VM202) on the following sites of a subject's both lower limbs. The number of injections depending on the administration site of VM202 shall be 56 intramuscular injections in total with 28 injections for 3 sites on the left and right lower limbs, respectively. See "Appendix 3. Administration Method of Investigational Product" for details.

- Peroneus longus muscle – 6 injections for left peroneus longus muscle and 6 injections for the right peroneus longus muscle
- Gastrocnemius muscle – 12 injections for the left gastrocnemius muscle and 12 injections for the right gastrocnemius muscle
- Tibialis anterior muscle – 10 injections for the left tibialis anterior muscle and 10 injections for the right tibialis anterior muscle

6.1.6.3 After Administration of Investigational Product

The following shall be performed after administration of the investigational product.

- **Vital signs**

  Vital signs including blood pressure (measurement shall be taken with the subject in a sitting position after resting for at least 10 minutes prior to measurement), temperature, heart rate per minute, and respiratory rate per minute shall be measured. The measurement results shall be recorded in the subjects' case report forms. This shall be performed at Visit 1 (screening) and throughout the clinical study every time a subject makes a visit (on the drug administration days consisting of Visits 2, 3, 4, and 5, it shall be performed both prior to administration and 2 ± 1 hours after administration). However, if there is a subject with abnormal findings in the test at Visit 3 (Day 14), a follow-up test may be performed at Day 30 depending on the decision of the principal investigator.

- **Adverse event assessment**

  Localized adverse events shall be checked at 2 ± 1 hours after administering the investigational product and on the day after administration.

6.1.7 Visit 6 (Interim Visit, Day 180 ± 7)

- **Adverse event assessment**

  Information on newly developed or worsened adverse events, or adverse events that disappeared since the last visit shall be collected.

- **Complete blood cell count**

  This shall be performed at Visit 1 and throughout the clinical study every time a subject makes a visit (shall be performed prior to administering the investigational product at Visits 2, 3, 4, and 5). However, if there is a subject with abnormal findings in the test at Visit 3 (Day 14), a complete blood cell count and a general blood chemistry test may be performed at Day 30 depending on the decision of the principal investigator.

  The complete blood cell count items are as follows:

  WBC, RBC, Hb, Hct, MCV, MCH, MCHC, PLT, ESR, MPV, differential count of WBC (Band neutrophil, Segmented neutrophil, Eosinophil, Basophil, Lymphocyte, Monocyte)

- **General blood chemistry test**

  This shall be performed at Visit 1 and throughout the clinical study every time a subject makes a visit (shall be performed prior to administering the investigational product at Visits 2, 3, 4, and 5). However, if there is a subject with abnormal findings in the test at Visit 3 (Day 14), a complete blood cell count and a general blood chemistry test may be performed at Day 30 depending on the decision of the principal investigator. Since a blood glucose test is included, the subject shall maintain an 8-hour fasting state prior to the test.

  The general blood chemistry test items are as follows:

total protein, albumin, globulin, A/G ratio, cholesterol, total bilirubin, AST, ALT, fasting glucose, BUN, creatinine, estimated GFR, Ca^{2+}, phosphate, Na^{+}, K^{+}, Cl^{-}, CRP, triglyceride, HDL-cholesterol, LDL-cholesterol

- **Sample collection for retrospective biomarker study**

  The serum of subjects shall be collected and stored frozen at -70°C. All blood samples are stored with unique numbers for retrospective biomarker study after all personal information is deleted.

- **Body measurement (weight measurement)**

  A subject's weight shall be measured at Visit 1 and throughout the clinical study every time a subject makes a visit (shall be performed prior to administering the investigational product at Visits 2, 3, 4, and 5).

- **Urinalysis with microscopy**

  This shall be performed at Visit 1 and throughout the clinical study every time a subject makes a visit (shall be performed prior to administering the investigational product at Visits 2, 3, 4, and 5). However, if there is a subject with abnormal findings in the test at Visit 3 (Day 14), urinalysis may be performed at Day 30 depending on the decision of the principal investigator. The urinalysis items are as follows:

  Color, turbidity, specific gravity, pH, albumin, glucose, ketones, bilirubin, blood, urobilinogen, nitrite, leukocyte esterase, microscopy (RBC, WBC, casts)

- **Vital signs**

  Vital signs including blood pressure (measurement shall be taken with the subject in a sitting position after resting for at least 10 minutes prior to measurement), temperature, heart rate per minute, and respiratory rate per minute shall be measured. The measurement results shall be recorded in the subjects' case report forms. This shall be performed at Visit 1 (screening) and throughout the clinical study every time a subject makes a visit (on the drug administration days consisting of Visits 2, 3, 4, and 5, it shall be performed both prior to administration and 2 ± 1 hours after administration). However, if there is a subject with abnormal findings in the test at Visit 3 (Day 14), a follow-up test may be performed at Day 30 depending on the decision of the principal investigator.

- **Concomitant medications survey**

  The brand names and ingredient names of concomitant medications shall be surveyed at every visit and recorded in the case report form. All medications and treatments administered within 6 months before Visit 1 (screening) shall be surveyed. Previous medications and previous treatments shall be defined as all previously collected medications and treatments before Visit 2 (first administration of investigational product). Concomitant medications and treatments shall

refer to all medications that have been administered at least once starting from Visit 2 (first administration of investigational product) and throughout the clinical study. The categories of collected medications and treatments shall follow this definition. The following shall also be surveyed for each medication:

- Brand name
- Indications for medication administration (reason for administration)
- Dose/strength, administration route, number of administrations
- Start and discontinuation dates of administration (whether to continue when the clinical study is terminated)

- **FDS**

  The functional disability scale (FDS) assesses a patient from 0 to 8 points as shown below depending on the patient's mobility.[68] The assessment days shall be at Visit 2 (Day 0), Visit 4 (Day 90), Visit 6 (Day 180), and Visit 7 (termination visit, Day 270) (assessment shall be performed prior to administration at Visit 2 [Day 0] and Visit 4 [Day 90]).

  0=normal;
  1=cramps and fatigability;
  2=inability to run;
  3=possible unaided;
  4=with cane;
  5=with crutch;
  6=with walker;
  7=wheelchair;
  8=bedridden.

- **ONLS leg scale**

  The overall neuropathy limitation scale (ONLS) is a tool for measuring the activity level of patients with peripheral neuropathy.[69] It is scored by separately categorizing arms and legs. Measurement shall be performed only on legs in this clinical study. The measurement day shall be at Visit 2 (Day 0), Visit 4 (Day 90), Visit 6 (Day 180), and Visit 7 (termination visit, Day 270) (measurement shall be performed prior to administration at Visit 2 [Day 0] and Visit 4 [Day 90]). See "Appendix 5. ONLS Leg scale" for details.

- **10MWT**

  This is a test that measures the time required for a subject to walk 10 meters.[70] A subject shall be made to walk at a desired speed while wearing shoes. The subject shall be allowed to use an assistive device that the subject normally uses, if any. The subject shall be made to walk a corridor that is 14 meters long, and the time taken to walk 10 meters shall be measured with a stopwatch provided by the sponsor (time taken to pass 10 meters excluding 2 meters each for the starting and ending portions). The assessment day shall be at Visit 2 (Day 0), Visit 4 (Day 90), Visit 6 (Day

180), and Visit 7 (termination visit, Day 270) (assessment shall be performed prior to administration at Visit 2 [Day 0] and Visit 4 [Day 90]).
The starting point at 2 meters, the point for ending measurement at 12 meters, and the point for end of walking at 14 meters shall be marked in advance along the corridor.
See "Appendix 6. 10MWT (10-meter walk test)" for details.

- **Neurologic exam**

Along with the assessments for the patient's sensation and motor symptoms, the following neurologic exam (motor system: muscle strength; sensory system: nociception, pallesthesia) shall be performed.

Table 15. Neurologic exam items and procedures

Test Item	Procedure
Muscle strength	Reduced muscle strength is referred to as weakness or paresis, and the loss of strength is referred to as paralysis.<br>To measure the muscle strength of limbs, the flexion (C5-6) and extension of wrist joint, extension of wrist (C6-8), grasping test (C7-T1), abduction of finger (C8-T1, ulnar nerve), and opposition of thumb (C8-T1, median nerve) shall be tested in the upper limbs. For the strength of intrinsic hand muscles, only the two muscles of the abductor pollicis brevis (APB) and the first dorsal interosseous (FDI) shall be evaluated, and the stronger of the two shall be scored.<br>In the lower limbs, the flexion (L2-L4), adduction (L2-L4), and extension (S1) of hip joint, the extension (L2-L4) and flexion (L4-S2) of knee, as well as the dorsiflexion (L4-5) and plantar flexion (S1) of ankle shall be tested.<br><br>The results of performing a muscle strength test of the limbs in a subject shall be indicated using the Medical Research Council (MRC) grade*.<br>* MRC grade (Motor power)<br>0: Complete paralysis<br>I: Flicker of contraction possible<br>II: Movement possible if gravity eliminated<br>III: Movement against gravity but not resistance<br>IV: Movement possible against some resistance<br>V: Power normal (it is not normally possible to overcome a normal adult's power)
Nociception	Stimulation shall be applied by alternately using tools with a dull end and a sharp end, and it shall be verified whether these can be distinguished. When comparing the left and right sides of the body, stimulation shall be applied to the same sites. Then, the subject shall be asked whether the sensations are the same.
Pallesthesia	Vibration shall be applied to a low-pitched tuning fork (a Rydel-Seiffer tuning fork shall be used), and this shall be placed on the distal joints of the hands and feet to verify that the subject feels the vibrating sensation. Pallesthesia is the first sensation lost in peripheral nerve disorders.

6.1.8 Visit 7 (Termination Visit, Day 270 ± 7) and Early Termination

- **Adverse event assessment**

Information on newly developed or worsened adverse events, or adverse events that disappeared since the last visit shall be collected.

- **Complete blood cell count**
  This shall be performed at Visit 1 and throughout the clinical study every time a subject makes a visit (shall be performed prior to administering the investigational product at Visits 2, 3, 4, and 5). However, if there is a subject with abnormal findings in the test at Visit 3 (Day 14), a complete blood cell count and a general blood chemistry test may be performed at Day 30 depending on the decision of the principal investigator.
  The complete blood cell count items are as follows:

  WBC, RBC, Hb, Hct, MCV, MCH, MCHC, PLT, ESR, MPV, differential count of WBC (Band neutrophil, Segmented neutrophil, Eosinophil, Basophil, Lymphocyte, Monocyte)

- **General blood chemistry test**
  This shall be performed at Visit 1 and throughout the clinical study every time a subject makes a visit (shall be performed prior to administering the investigational product at Visits 2, 3, 4, and 5). However, if there is a subject with abnormal findings in the test at Visit 3 (Day 14), a complete blood cell count and a general blood chemistry test may be performed at Day 30 depending on the decision of the principal investigator. Since a blood glucose test is included, the subject shall maintain an 8-hour fasting state prior to the test.
  The general blood chemistry test items are as follows:

  total protein, albumin, globulin, A/G ratio, cholesterol, total bilirubin, AST, ALT, fasting glucose, BUN, creatinine, estimated GFR, Ca^{2+}, phosphate, Na^{+}, K^{+}, Cl^{-}, CRP, triglyceride, HDL-cholesterol, LDL-cholesterol

- **Sample collection for retrospective biomarker study**
  The serum of subjects shall be collected and stored frozen at -70°C. All blood samples are stored with unique numbers for retrospective biomarker study after all personal information is deleted.

- **Body measurement (weight measurement)**
  A subject's weight shall be measured at Visit 1 and throughout the clinical study every time a subject makes a visit (shall be performed prior to administering the investigational product at Visits 2, 3, 4, and 5).

- **Urinalysis with microscopy**
  This shall be performed at Visit 1 and throughout the clinical study every time a subject makes a visit (shall be performed prior to administering the investigational product at Visits 2, 3, 4, and 5). However, if there is a subject with abnormal findings in the test at Visit 3 (Day 14), urinalysis may be performed at Day 30 depending on the decision of the principal

investigator. The urinalysis items are as follows:

Color, turbidity, specific gravity, pH, albumin, glucose, ketones, bilirubin, blood, urobilinogen, nitrite, leukocyte esterase, microscopy (RBC, WBC, casts)

- **Vital signs**

  Vital signs including blood pressure (measurement shall be taken with the subject in a sitting position after resting for at least 10 minutes prior to measurement), temperature, heart rate per minute, and respiratory rate per minute shall be measured. The measurement results shall be recorded in the subjects' case report forms. This shall be performed at Visit 1 (screening) and throughout the clinical study every time a subject makes a visit (on the drug administration days consisting of Visits 2, 3, 4, and 5, it shall be performed both prior to administration and 2 ± 1 hours after administration). However, if there is a subject with abnormal findings in the test at Visit 3 (Day 14), a follow-up test may be performed at Day 30 depending on the decision of the principal investigator.

- **Concomitant medications survey**

  The brand names and ingredient names of concomitant medications shall be surveyed at every visit and recorded in the case report form. All medications and treatments administered within 6 months before Visit 1 (screening) shall be surveyed. Previous medications and previous treatments shall be defined as all previously collected medications and treatments before Visit 2 (first administration of investigational product). Concomitant medications and treatments shall refer to all medications that have been administered at least once starting from Visit 2 (first administration of investigational product) and throughout the clinical study. The categories of collected medications and treatments shall follow this definition. The following shall also be surveyed for each medication:

  - Brand name
  - Indications for medication administration (reason for administration)
  - Dose/strength, administration route, number of administrations
  - Start and discontinuation dates of administration (whether to continue when the clinical study is terminated)

- **Neurologic exam**

  This shall be performed for CMTNS-v2 measurement, and the investigator shall perform the neurologic exam (motor system: muscle strength; sensory system: nociception, pallesthesia) on a subject as follows:

Table 11. Neurologic exam items and procedures for CMTNS-v2 measurement

Test Item	Procedure
Muscle strength	Reduced muscle strength is referred to as weakness or paresis, and the loss of strength is referred to as paralysis.<br>To measure the muscle strength of limbs, the flexion (C5-6) and

	extension of wrist joint, extension of wrist (C6-8), grasping test (C7-T1), abduction of finger (C8-T1, ulnar nerve), and opposition of thumb (C8-T1, median nerve) shall be tested in the upper limbs. For the strength of intrinsic hand muscles, only the two muscles of the abductor pollicis brevis (APB) and the first dorsal interosseous (FDI) shall be evaluated, and the stronger of the two shall be scored.<br>In the lower limbs, the flexion (L2-L4), adduction (L2-L4), and extension (S1) of hip joint, the extension (L2-L4) and flexion (L4-S2) of knee, as well as the dorsiflexion (L4-5) and plantar flexion (S1) of ankle shall be tested.<br><br>The results of performing a muscle strength test of the limbs in a subject shall be indicated using the Medical Research Council (MRC) grade*.<br>* MRC grade (Motor power)<br>0: Complete paralysis<br>I: Flicker of contraction possible<br>II: Movement possible if gravity eliminated<br>III: Movement against gravity but not resistance<br>IV: Movement possible against some resistance<br>V: Power normal (it is not normally possible to overcome a normal adult's power)
Nociception	Stimulation shall be applied by alternately using tools with a dull end and a sharp end, and it shall be verified whether these can be distinguished. When comparing the left and right sides of the body, stimulation shall be applied to the same sites. Then, the subject shall be asked whether the sensations are the same.
Pallesthesia	Vibration shall be applied to a low-pitched tuning fork (a Rydel-Seiffer tuning fork shall be used), and this shall be placed on the distal joints of the hands and feet to verify that the subject feels the vibrating sensation. Pallesthesia is the first sensation lost in peripheral nerve disorders.

- **CMTNS-v2**

Charcot-Marie-Tooth Neuropathy Score-version 2 (CMTNS-v2) is a measurement tool for evaluating the severity of disease.[66] Measurements shall be taken for 9 items which include 3 items for disease symptoms, 4 items for signs, and 2 items for neurophysiological testing. The severity of disease shall be classified according to scores as mild (≤10), moderate (11 to 20), and severe (>20). This clinical study shall target mild to moderate patients. This shall be performed at Visit 1 (screening), Visit 2 (Day 0), and Visit 7 (termination visit, Day 270) (shall be performed prior to administration at Visit 2 [Day 0]). See "Appendix 4. CMTNS-v2" for details.

- **Anti-HGF Ab test**

The antibody test for hepatocyte growth factor shall be performed at Visit 2 (Day 0) and Visit 7 (termination visit, Day 270) (shall be performed prior to administration at Visit 2 [Day 0]). The serum of subjects shall be collected and stored frozen at -70°C. When the collection of blood from all subjects is completed, the samples shall be sent to the central laboratory for batch analysis.

- **MRI leg**

The muscles of lower limbs shall be imaged, and the fatty infiltration level of the leg muscles injected with the investigational product shall be measured and evaluated as fat content value (%) at one level for each muscle.[69]

The test days shall be at Visit 2 (Day 0) and Visit 7 (termination visit, Day 270) (shall be performed prior to administration at Visit 2 [Day 0]). Considering the schedule, etc., of the institution, it shall be performed optionally at the early termination visit and unscheduled visits, and if it is not performed, the reasons shall be recorded in the case report form.

- **FDS**

The functional disability scale (FDS) assesses a patient from 0 to 8 points as shown below depending on the patient's mobility.[68] The assessment days shall be at Visit 2 (Day 0), Visit 4 (Day 90), Visit 6 (Day 180), and Visit 7 (termination visit, Day 270) (assessment shall be performed prior to administration at Visit 2 [Day 0] and Visit 4 [Day 90]).

0=normal;
1=cramps and fatigability;
2=inability to run;
3=possible unaided;
4=with cane;
5=with crutch;
6=with walker;
7=wheelchair;
8=bedridden.

- **ONLS leg scale**

The overall neuropathy limitation scale (ONLS) is a tool for measuring the activity level of patients with peripheral neuropathy.[69] It is scored by separately categorizing arms and legs. Measurement shall be performed only on legs in this clinical study. The measurement day shall be at Visit 2 (Day 0), Visit 4 (Day 90), Visit 6 (Day 180), and Visit 7 (termination visit, Day 270) (measurement shall be performed prior to administration at Visit 2 [Day 0] and Visit 4 [Day 90]). See "Appendix 5. ONLS Leg scale" for details.

- **10MWT**

This is a test that measures the time required for a subject to walk 10 meters.[70] A subject shall be made to walk at a desired speed while wearing shoes. The subject shall be allowed to use an assistive device that the subject normally uses, if any. The subject shall be made to walk a corridor that is 14 meters long, and the time taken to walk 10 meters shall be measured with a stopwatch provided by the sponsor (time taken to pass 10 meters excluding 2 meters each for the starting and ending portions). The assessment day shall be at Visit 2 (Day 0), Visit 4 (Day 90), Visit 6 (Day 180), and Visit 7 (termination visit, Day 270) (assessment shall be performed prior to administration at Visit 2 [Day 0] and Visit 4 [Day 90]).

The starting point at 2 meters, the point for ending measurement at 12 meters, and the point for end of walking at 14 meters shall be marked in advance along the corridor.
See "Appendix 6. 10MWT (10-meter walk test)" for details.

- **Electroneurography (CMAP, SNAP, NCV)**
  The test days shall be at Visit 2 (Day 0) and Visit 7 (termination visit, Day 270) (shall be performed prior to administration at Visit 2 [Day 0]).
  See "Appendix 7. Nerve Conduction Study (NCS)" for details.

- **Use of assistive devices and types**
  The assistive devices used by the subjects shall be surveyed at Visit 1 (screening) and at Visit 7 (termination visit, Day 270). Specialized shoes, braces for lower limbs, crutches, canes, walkers, wheelchairs, etc., fall under these devices. Even if new additional assistive devices are used during the clinical study, they shall not be included in adverse events.

6.1.9 Unscheduled Visits

- **Adverse event assessment**
  Information on newly developed or worsened adverse events, or adverse events that disappeared since the last visit shall be collected.

- **Complete blood cell count**
  This shall be performed at Visit 1 and throughout the clinical study every time a subject makes a visit (shall be performed prior to administering the investigational product at Visits 2, 3, 4, and 5). However, if there is a subject with abnormal findings in the test at Visit 3 (Day 14), a complete blood cell count and a general blood chemistry test may be performed at Day 30 depending on the decision of the principal investigator. The complete blood cell count items are as follows:

  WBC, RBC, Hb, Hct, MCV, MCH, MCHC, PLT, ESR, MPV, differential count of WBC (Band neutrophil, Segmented neutrophil, Eosinophil, Basophil, Lymphocyte, Monocyte)

- **General blood chemistry test**
  This shall be performed at Visit 1 and throughout the clinical study every time a subject makes a visit (shall be performed prior to administering the investigational product at Visits 2, 3, 4, and 5). However, if there is a subject with abnormal findings in the test at Visit 3 (Day 14), a complete blood cell count and a general blood chemistry test may be performed at Day 30 depending on the decision of the principal investigator. Since a blood glucose test is included, the subject shall maintain an 8-hour fasting state prior to the test. The general blood chemistry test items are as follows:

total protein, albumin, globulin, A/G ratio, cholesterol, total bilirubin, AST, ALT, fasting glucose, BUN, creatinine, estimated GFR, Ca^{2+}, phosphate, Na^{+}, K^{+}, Cl^{-}, CRP, triglyceride, HDL-cholesterol, LDL-cholesterol

- **Body measurement (weight measurement)**
  A subject's weight shall be measured at Visit 1 and throughout the clinical study every time a subject makes a visit (shall be performed prior to administering the investigational product at Visits 2, 3, 4, and 5).

- **Urinalysis with microscopy**
  This shall be performed at Visit 1 and throughout the clinical study every time a subject makes a visit (shall be performed prior to administering the investigational product at Visits 2, 3, 4, and 5). However, if there is a subject with abnormal findings in the test at Visit 3 (Day 14), urinalysis with microscopy may be performed at Day 30 depending on the decision of the principal investigator.
  The urinalysis items are as follows:

  Color, turbidity, specific gravity, pH, albumin, glucose, ketones, bilirubin, blood, urobilinogen, nitrite, leukocyte esterase, microscopy (RBC, WBC, casts)

- **Vital signs**
  Vital signs including blood pressure (measurement shall be taken with the subject in a sitting position after resting for at least 10 minutes prior to measurement), temperature, heart rate per minute, and respiratory rate per minute shall be measured. The measurement results shall be recorded in the subjects' case report forms. This shall be performed at Visit 1 (screening) and throughout the clinical study every time a subject makes a visit (on the drug administration days consisting of Visits 2, 3, 4, and 5, it shall be performed both prior to administration and 2 ± 1 hours after administration). However, if there is a subject with abnormal findings in the test at Visit 3 (Day 14), a follow-up test may be performed at Day 30 depending on the decision of the principal investigator.

- **Concomitant medications survey**
  The brand names and ingredient names of concomitant medications shall be surveyed at every visit and recorded in the case report form. All medications and treatments administered within 6 months before Visit 1 (screening) shall be surveyed. Previous medications and previous treatments shall be defined as all previously collected medications and treatments before Visit 2 (first administration of investigational product). Concomitant medications and treatments shall refer to all medications that have been administered at least once starting from Visit 2 (first administration of investigational product) and throughout the clinical study. The categories of collected medications and treatments shall follow this definition. The following shall also be surveyed for each medication:

- Brand name
- Indications for medication administration (reason for administration)
- Dose/strength, administration route, number of administrations
- Start and discontinuation dates of administration (whether to continue when the clinical study is terminated)

6.2 Assessment Items

1) Safety and tolerability assessment items

(1) Adverse events

- All adverse events that manifest after administration of the investigational product shall be collected.
- At Visits 3, 4, and 5 (2nd, 3rd, and 4th administration sessions of the investigational product), adverse events shall be assessed and collected before and after administration of the investigational product.

(2) Laboratory tests (complete blood cell count/general blood chemistry/urinalysis tests)

(3) Vital signs

2) Efficacy assessment items

(1) Changes in severity of disease

- CMTNS-v2 (Charcot-Marie-Tooth Neuropathy Score version 2)
- FDS (functional disability scale)

(2) Changes in lower limb function

- ONLS (overall neuropathy limitation score) leg scale
- 10MWT (10-meter walk test)

(3) Changes in fatty infiltration level of lower limb muscles

- MRI leg

(4) Nerve regeneration potential

- CMAP (compound motor nerve action potential)
- SNAP (compound sensory nerve action potential)
- NCV (nerve conduction velocity)

(5) HGF antibody generation by VM202

7 Adverse Event

7.1 Definition

An adverse event (AE) is an unfavorable and unintended symptom (e.g., motion sickness), sign (e.g., hepatomegaly), or clinically meaningful anomaly (e.g., abnormal laboratory finding) that occurred during the clinical study, whether or not caused by the study intervention.

Adverse Drug Reaction (ADR)
An Adverse Drug Reaction (ADR) is a harmful and unintended reaction that occurred at any dose of the investigational product, of which the causality with the investigational product cannot be denied.

Adverse Events of Special Interest, AESI
An adverse event of special interest (serious or nonserious) is one of scientific and medical concern specific to the sponsor's product or program, for which ongoing monitoring and rapid communication by the investigator to the sponsor can be appropriate.

Serious AE·ADR
A Serious AE·ADR is an AE or ADR that occurred at any dose of the investigational product, which results in any of the following:

(1) Death or life-threatening;
(2) Inpatient hospitalization or prolongation of existing hospitalization;
(3) Permanent or significant disability/incapacity;
(4) Congenital anomaly/birth defect;
(5) Other medically significant events including drug dependence/abuse or hematologic disease, etc.

The term “life-threatening” refers to an event in which the subject was at risk of instant death at the time of the event.

When the subject has been stayed at the emergency room for treatment for more than 24 hours, the criteria for hospitalization are considered to be met. Hospitalization scheduled before the initial investigational product administration or hospitalization for cosmetic surgery is not considered an AE or SAE. Elective surgery during which no AEs occur, subsequent hospitalization from such surgery, and hospitalization in care hospitals for recovery are not considered SAEs. However, unscheduled hospitalizations or hospitalizations resulting from AEs

are considered SAEs.

If an event occurs that is medically considered to have a significant effect on the safety and health of the subject, even if not listed above, it should be determined whether to consider it an SAE according to the medical judgment of the physician in charge and relevant experts, and appropriate interventions should be taken accordingly.

Unexpected Adverse Event

An unexpected adverse event refers to an AE that differs from or is not stated in terms of the degree or aspects of the ADR, considering available drug-related information, such as the Investigator's Brochure or attachments of the investigational product.
An expected event refers to a case where an AE which occurred even before the use of the investigational product is observed, which is not accompanied by medical history or concomitant medications and is described in the Investigator's Brochure.

Expected Adverse Event

An expected adverse event refers to an AE that has been observed and confirmed in previous studies and described in the available information related to the product such as the Investigator's Brochure, the protocol or insert, etc., which does not include cases accompanied by past medical history or concomitant medications, but includes cases where predicted AEs repeatedly occur over time due to certain disease conditions.

7.2 Expected Adverse Event

In this clinical study, predicted side effects can be classified into two categories: "AE due to Charcot-Marie-Tooth," the underlying disease; and "AE that may occur after investigational product administration."

Figure 9 is intended as reference for the investigator evaluating the cause or causality of AEs. The investigator must take appropriate interventions against AEs that occur during the clinical study, evaluate the causality according to the algorithm of "Appendix 8. Method of assessing causality of adverse events," and report the AE.

AEs such as pain, dyspnea, skin ulcer, or muscle cramps may be observed both after the administration of the investigational product and as symptoms of the underlying disease, Charcot-Marie-Tooth. The investigator must carefully determine whether the AE is caused by the underlying disease or by administration of the investigational product.

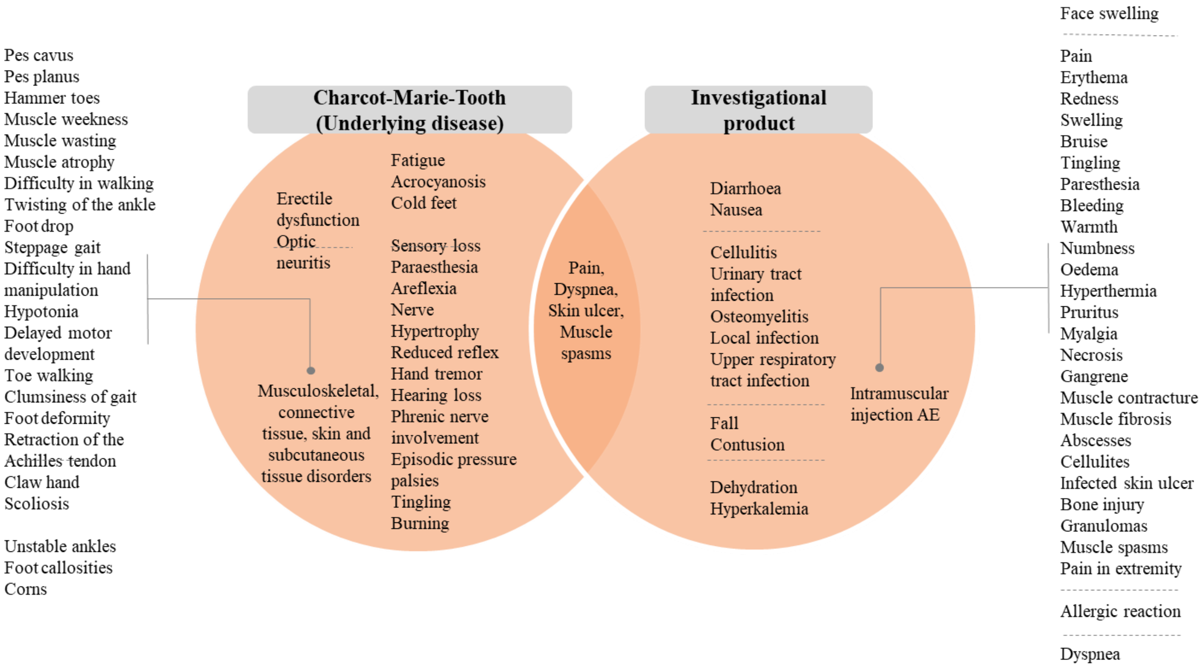

Figure 9 AEs that may occur during clinical study

7.2.1 Adverse events that may be caused by Charcot-Marie-Tooth, the underlying disease

Since this clinical study targets patients with mild to moderate severity of Charcot-Marie-Tooth disease, the progression or deterioration of the diseases may result in the report of the following AEs, other than pain, the most common symptom of this disease.[71][72][73][74][75][76][77]

Table 16 Adverse events that may be caused by Charcot-Marie-Tooth

System of Organ	Adverse event
General disorders and administration site conditions	Pain<br>Fatigue<br>Difficulty in walking<br>Steppage gait<br>Ulceration
Cardiac disorders	Acrocyanosis
Vascular disorders	Cold feet
Musculoskeletal and connective tissue disorders	Pes cavus<br>Pes planus<br>Hammer toes<br>Muscle weakness<br>Muscle wasting<br>Muscle atrophy<br>Twisting of the ankle

System of Organ	Adverse event
	Difficulty in hand manipulation<br>Cramp<br>Scoliosis<br>Toe walking<br>Unstable ankles<br>Foot deformity<br>Retraction of the Achilles tendon<br>Claw hand
Skin and subcutaneous tissue disorders)	Foot callosities<br>Corns
Nervous system disorders	Delayed motor development
	Clumsiness of gait
	Foot drop<br>Hypotonia<br>Sensory loss<br>Paraesthesia<br>Areflexia<br>Nerve hypertrophy<br>Reduced reflexes<br>Hand tremor<br>Phrenic nerve involvement<br>Episodic pressure palsies<br>Tingling<br>Burning<br>Optic neuritis
Respiratory, thoracic and mediastinal disorders	Respiratory failure
Reproductive system and breast disorders	Erectile dysfuction
Ear and labyrinth disorders	Hearing loss

7.2.2 Adverse events that may be caused by administration of investigational product

The investigational product is administered via intramuscular injection in the lower extremities, which may cause adverse events. In addition, the investigator must note that the predicted adverse events described in the Investigator's Brochure may occur.

1) Adverse events due to intramuscular injection

Since the investigational product used in this clinical trial is administered via intramuscular injection in the lower extremities, adverse events may temporarily occur due to intramuscular injection.[78][79][80][81][82][83] Injection Site Reaction is defined as the adverse event that is observed at musculoskeletal or skin around injection site within 24 hours after the intramuscular injection of IP.

Table 17 Adverse events caused by intramuscular injection [78-83]

System of Organ	Adverse event
General disorders	Facial swelling
Musculoskeletal and skin disorder	Pain at injection sites<br>Erythema at injection sites<br>Redness at injection sites<br>Swelling at injection sites<br>Tingling at injection sites<br>Bleeding at injection sites<br>Warmth at injection sites<br>Numbness at injection sites<br>Oedema at injection sites<br>Hyperthermia at injection sites<br>Pruritus at injection sites<br>Myalgia at injection sites<br>Necrosis at injection sites<br>Gangrene at injection sites<br>Muscle contracture at injection sites<br>Muscle fibrosis at injection sites<br>Abscesses at injection sites<br>Cellulites at injection sites<br>Bone injury<br>Granulomas
Immune system disorder	Allergic reaction
Respiratory, thoracic and mediastinal disorders	Dyspnea

2) Predicted adverse events described in the Investigator's Brochure

The investigational product has been used in clinical studies for indications including critical limb ischemia (CLI), painful diabetic peripheral neuropathy (DPN), ischemic heart disease (IHD), and amyotrophic lateral sclerosis (ALS). The most frequent (5% or more) AEs in the previous clinical studies are as follows. As the same investigational product is used in this clinical study, the investigator must note that the following AEs may occur:

Table 18 Predicted adverse events described in the Investigator's Brochure

System of Organ	Adverse event
Gastrointestinal disorder	Diarrhoea<br>Nausea
Infections and infestations	Cellulitis<br>Urinary tract infection<br>Osteomyelitis<br>Localized infection<br>Upper respiratory tract infection
Injury, poisoning and procedural complications	Falls<br>Contusion
Musculoskeletal and connective tissue disorders	Muscle spasms<br>Pain in extremity

System of Organ	Adverse event
Metabolism and nutrition disorders	Dehydration<br>Hyperkalemia
Skin and subcutaneous tissue disorders	Skin ulcer<br>Infected skin ulcer<br>pain at injection sites<br>Injection site paresthesia<br>Injection site itching<br>Injection site erythema<br>Injection site bruising

7.2.3 Summary of AEs that occurred in clinical studies on other indications using the investigational product

Prior to this clinical study, the same investigational product was used in the phase 1 and phase 2 clinical studies for CLI, phase 1/2 and phase 2 clinical studies for painful DPN, phase 1/2 clinical study for ALS, and phase 1 clinical study for angina pectoris.

Among the AEs that occurred in each clinical study, the ones considered to be caused by the investigational product are as follows. AEs considered to be caused by the investigation product means AEs that have been judged to be Definitely Related, Probably Related, Possibly Related, and Unlikely.

The investigator should become familiar with the AEs determined to have the following causality and use them as reference for judging the causality with the investigational product used in this clinical study. Attachment 9 is a table that summarizes all AEs that occurred during administration of the investigational product in other indications regardless of causality. The investigator may use that data as reference if necessary.

1) Phase 1 clinical study for CLI

Forty-one AEs occurred to 12 subjects who were administered with the investigational product, and among them, a total of four AEs were reported as having a causality with the investigational product.

Table 19 AEs that were reported as having causality in CLI phase1

System of Organ	Adverse event	VM202(n=12)		
		n	%	case(s)
General disorders and administration site conditions	Injection site bruising[1]	1	11.11%	1
Neoplasms benign,	Metastases to Liver[3]	1	11.11%	1

System of Organ	Adverse event	VM202(n=12)		
		n	%	case(s)
malignant and unspecified	Small Cell Lung Cancer – Stage Unspecified[3]	1	11.11%	1
	Colon Cancer[2]	1	11.11%	1

[1]Probably Related, [2]Possibly Related, [3]Unlikely

* SAE

2) Phase 2 clinical study for CLI

Four hundred and thirty-eight AEs occurred to 49 out of 52 subjects who were administered with the investigational product, and a total of 22 AEs which were reported as having a causality with the investigational product occurred to 41 subjects who were administered with the investigational product.

Table 20 AEs that were reported as having causality in CLI phase2

System of Organ	Adverse event	VM202(n=41)		
		n	%	case(s)
General disorders and administration site conditions	Asthenia[2]	1	1.92%	1
	Injection site pain[2]	1	1.92%	1
	Injection site reaction[1,2]	2	3.85%	3
	Pain[2]	1	1.92%	1
Eye disorders	Diabetic retinal oedema[2]	1	1.92%	1
	Macular cyst[2]	1	1.92%	1
Infections and infestations	Vulvovaginal candidiasis[2]	1	1.92%	1
Investigations	Blood glucose increase[2]	1	1.92%	1
	Blood creatinine increased[2]	1	1.92%	1
	Blood urea increased[2]	1	1.92%	1
Musculoskeletal and connective tissue disorders	Muscle spasms[2]	1	1.92%	1
	Musculoskeletal stiffness[2]	1	1.92%	1
	Pain in extremity[2]	3	5.77%	3
Skin and subcutaneous tissue disorders	Telangiectasia[2]	1	1.92%	1
	Dermatitis contact[2]	1	1.92%	1
Nerve system disorders	Spider vein[2]	1	1.92%	1
	Deep vein thrombosis[2*]	1	1.92%	1
	Dysgeusia[2]	1	1.92%	1

[1] Definitely Related, [2] Possibly Related, *SAE

3) Phase 1/2 clinical study for painful DPN

Fifteen AEs occurred to 7 out of 12 subjects who were administered with the investigational product, and among them, a total of 6 AEs were reported as having a causality with the investigational product.

Table 21 AEs that were reported as having causality in painful DPN phase1/2

System of Organ	Adverse event	VM202(n=12)		
		n	%	Case(s)
General disorders and administration site conditions	Pain at injection sites[1]	1	8.33%	1
Gastrointestinal disorders	Diarrhea[1]	1	8.33%	1
	Dry mouth[1]	1	8.33%	1
Eye disorders	Dry eyes[1]	1	8.33%	2
	Parafoveal retinal vascular leakage OU[1]	1	8.33%	1

[1] Possibly Related

4) Phase 2 clinical study for painful DPN

Two hundred and two AEs occurred to 69 out of 103 subjects who were administered with the investigational product, and among them, a total of 48 AEs which were reported as having a causality with the investigational product occurred to 82 subjects who were administered with the investigational product.

Table 22. AEs that were reported as having causality in painful DPN phase 2

System of Organ	Adverse event	VM202(n=82)		
		n	%	Case(s)
General disorders and administration site conditions	Ankle/leg edema[1,2]	3	3.65%	3
	Chest pain[1,2]	3	3.65%	3
	Fever[1]	1	1.21%	1
	Malaise[1]	1	1.21%	1
	Weakness[1]	1	1.21%	1
Gastrointestinal disorders	Nausea[1,2]	4	4.87%	5
Musculoskeletal and connective tissue disorders	Calf pain/ spasm[1]	1	1.21%	2
	Foot cramps/ pain[1,2]	5	6.09%	12
	Leg cramps/ pain[1,2]	2	2.43%	3
Eye disorders	Non proligerative diabetic retinopathy[1]	1	1.21%	1
	Posterior vitreous detachment[1]	1	1.21%	1
Investigation	Abnormal lab values[1,2]	8	9.75%	8
Neoplasms benign, malignant and unspecified	Breast tumor, benign [1,2]	2	2.43%	2
Nervous system disorders	Worsening peripheral neuropathy[1]	1	1.21%	1
	Headache[1,2]	2	2.43%	2
Metabolism and nutrition disorders	Hypoglycemia[1,2,]	2	2.43%	2

[1]Possibly Related, [2] Not Related

[1,2] Simultaneous marking indicates cases in which both Possibly Related and Not Related were reported.

5) Phase 1/2 clinical study for ALS

Fifty-seven AEs occurred to 17 out of 18 subjects who were administered with the investigational product, and among them, a total of 1 AE was reported as having a causality with the investigational product.

Table 23 AEs that were reported as having causality in ALS phase 1/2

System of Organ	Adverse event	VM202(n=18)		
		n	%	Case(s)
General disorders and administration site conditions	Increased weakness both lower extremities [1]	1	5.55%	1

[1] Possibly Related

6) Phase 1 clinical study for Ischemic heart disease

Fifty-eight AEs occurred to 9 subjects who were administered with the investigational product, but none were reported as having a causality with the investigational product.

7.3 Precautions

VM202 must not be used on subjects with severe infection. Women of childbearing potential who are administered with VM202 must be careful to avoid pregnancy.

7.4 Criteria and methods for assessing AEs

All AEs occurring during the 270-day (9 months) follow-up period from the first investigational product administration must be recorded in the AE report of the case report form, regardless of severity.

Abnormal lab test results and abnormal findings of vital signs that are considered clinically significant shall be recorded as AEs. They include the following information:

- Description of the AE
- Onset Date (start date)
- Resolved Date (end date)
- Frequency of occurrence
- Severity
- Seriousness (Yes/No)
- Treatment for the AE
- Outcomes
- Relationship with the investigational product, administration procedure of the investigational product, and underlying diseases

If an AE or SAE that occurs within the nine-month follow-up period is not resolved within this period and additional follow-up is required, follow-up shall be conducted until the AE is resolved. The content of the data collection is the same as the items collected during the nine-month follow-up. All AEs and SAEs must be tracked until they are resolved, or until the condition is stabilized. The investigator must maintain records of additional studies that may reveal the circumstances and causes of AEs or SAEs, including additional laboratory tests, investigations, histological tests and consultations with other medical experts. The sponsor or a designated person may request the investigator to carry out or plan additional tests or assessments.

7.4.1 Causality with investigational product

When an AE occurs, the investigator evaluates its relationship with the investigational product in accordance with the following criteria and describes the investigator's opinion. When doing so, AEs except for those "not related" are categorized as ones in which the relationship with the investigational product cannot be excluded ("related"). Appendix 8 is an adaptation of Table 17 below, which is the general standard for assessing causality, for this clinical study, and the investigator evaluates causality by referring to this algorithm.

Table 24 Causality with investigational product

Definitely related	▪ There is evidence that the investigational product has been administered and the time sequence of the AE onset is reasonable;<br>▪ The AE is most probably caused by the investigational product compared with any other reason;<br>▪ The AE disappears when administration of the investigational product is suspended;<br>▪ Re-administration (performed only if possible) result is positive;<br>▪ The AE shows a pattern consistent with the information already known about the investigational product or drugs of the same family.
Probably related	▪ There is evidence that the investigational product has been administered and the time sequence of the AE onset is reasonable;<br>▪ The AE is more probably caused by the investigational product

	than for any other reason;<br>▪ The AE disappears when administration of the investigational product is suspended.
Possibly related	▪ There is evidence that the investigational product has been administered and the time sequence of the AE onset is reasonable;<br>▪ The AE is caused by the investigational product at the same level as other probable reasons;<br>▪ The AE disappears when administration of the investigational product is suspended (if performed);
Unlikely	▪ There is evidence that the investigational product has been administered;<br>▪ There are other causes with higher possibility than the investigational product;<br>▪ The administration suspension (if performed) result is negative or ambiguous;<br>▪ The re-administration (if performed) result is negative or ambiguous;
Not related	▪ The subject has not been administered with the investigational product;<br>▪ The time sequence of the administration and the AE onset is not reasonable;<br>▪ There are other obvious causes for the AE;
Unassessable	▪ There is insufficient evidence to determine the relationship;<br>▪ Information is insufficient or conflicting to determine, and cannot be supplemented or verified;

7.4.2 Severity of AE

Severity of an AE is categorized according to NCI-CTCAE version5.0. AEs that cannot be categorized by the NCI-CTCAE are assessed by the following five grades:

Table 25 General classification in NCI-CTCAE V5.0

Grade	Description of Severity
**1**	Mild: No symptoms or mild symptoms; only clinical or diagnostic observations possible; no therapeutic intervention is needed.
**2**	Moderate: Minimal, local, or non-invasive treatment needed; daily activities, including meal preparation and shopping, are limited.
**3**	Severe: Medically meaningful but is not immediately life-threatening; hospitalization or prolongation of hospitalization; disability; not bedridden but daily activities, such as bathing, putting on or taking off clothes, eating, going to the bathroom, and taking medication, are limited.
**4**	Life-threatening consequences: immediate treatment is needed.
**5**	Death: AE related to death

7.4.3 Reporting, collection, and recording of AEs

The principal investigator must educate the sub-investigator and subjects or representatives of the subject about all adverse events that may occur after the administration of the investigational product and educate them to report all reactions that occur after administration.

1) All AEs that occur from administration of investigational product to follow-up visits (until end of the clinical trial) are collected. However, if a sign, symptom, or disease that occurred before the administration is worsened after the administration, it shall also be considered an AE.
2) An AE must be reported including the name of the AE, start date and end date, severity, actions related to investigational product, progress, causality with the investigational product, remedial treatment, and whether it is an SAE.
3) When documenting an AE, the investigator uses a comprehensive diagnosis or symptom name using standard medical terminology instead of each symptom or sign.
4) AEs are observed for the study period of nine months (day 0 to 270). If an AE or SAE that occurs within the nine-month follow-up period is not resolved within this period, follow-up shall be conducted until the AE is resolved.

7.4.3.1 Collection, and recording of AESI(Adverse Events of Special Interest)

All AESIs that occur throughout the clinical trial are collected, the following events are classified as AESI, and the Helixmith PV team manages them as safety information data.

1) Coronavirus Disease-19 (COVID-19)
2) Injection Site Reactions

7.4.4 Reporting and recording of SAEs

7.4.4.1 Reporting of SAEs

The Investigator prepares and submits the "SAE Report Form" through the eCRF system within 24 hours of recognizing any SAEs regardless of causality with the investigational product. When the Investigator's electronic signature and submission are complete, a notification e-mail is sent to Dt&SanoMedics PV team and Helixmith Co., Ltd. If the SAE Report cannot be prepared and submitted through the eCRF system due to unavoidable circumstances, the "SAE Report Form" is prepared and submitted to the safety information contact person below by fax or email:

Safety reporting contact information

Contact person	Dt&SanoMedics PV Team
Fax	+82-2-566-3222
e-mail	pv@dtnsm.com
address	15th Floor, 126, Teheran-ro, Gangnam-gu, Seoul, South Korea

The initial SAE Report must include the following four minimal elements of information:

1) An identifiable reporter
2) An identifiable patient
3) A suspect drug
4) A serious adverse event

The following are the responsibilities of each person in charge regarding the SAEs occurring during the clinical study period:

1) Principal Investigator

When an SAE occurs, the principal investigator immediately reports it to the Sponsor (within 24 hours of recognizing). When important additional information regarding the SAE is available later, the principal investigator must submit an additional report including details, within 24 hours of recognizing the additional information. Or, in case of a suspected unexpected serious adverse reaction, it must be expeditiously reported to the Sponsor and IRB. When a death case is reported, the principal investigator must provide additional information such as the autopsy report (only if an autopsy was conducted) and death certificate to the Sponsor and IRB.

2) Sub-Investigator(s)

When an SAE occurs, the sub-investigator(s) must immediately report it to the principal investigator and Sponsor and later submit an additional report containing details. Or, in case of a suspected unexpected serious adverse reaction, it must be expeditiously reported to the principal Investigator, Sponsor, and IRB.

3) Institutional Review Board (IRB)

The IRB requires the principal investigator to take necessary actions if there is a suspected unexpected serious adverse reaction or any new information that may negatively affect the safety of subjects or operation of the clinical study.

4) Sponsor

(1) The Sponsor reports any suspected unexpected serious adverse reactions to other relevant investigators, the IRB (only if the Investigator has not reported them to the IRB or there is a need to change content of the report), and the Ministry of Food and Drug Safety (MFDS) within 15 days of receiving a report from the principal investigator or sub-investigator(s) on or recognizing them. Or, in the event of death or life-threatening, the Sponsor must report on it within seven days of receiving a report on or recognizing it, and report further detailed information within eight days of the initial reporting. When submitting a SUSAR report, information received from the principal investigator or sub-investigator(s) shall be attached.

(2) The Sponsor must periodically report additional safety information related to the above report until the SUSAR is concluded (the disappearance of the SUSAR or inability to follow up). The investigator must actively cooperate in providing data and information regarding the report.

7.4.4.2 Reporting Suspected Unexpected Serious Adverse Reaction (SUSAR)

The Sponsor has the obligation to continuously conduct assessment on the safety of

the investigational product used in the clinical study.

The Sponsor must expeditiously report any SUSARs to the other relevant investigators, the IRB, and the MFDS within the period specified as follows:

(1) The Sponsor must expeditiously report any SUSARs to the other relevant investigators, the MFDS, and, when necessary, the IRB within the period specified as follows:

A) Death or life-threatening: Within seven days of receiving a report on or recognizing the event. Or, if the initial report lacks any of the information required in the attached Form 77, the ADR Report, including the name of the ADR, final observation result, and ADR summary, an additional report must be submitted with detailed information on the ADR within 15 days of receiving report on or recognizing the event.

B) Others: within 15 days of the Sponsor receiving report on or recognizing the event.

(2) If there is additional information on the adverse drug reaction reported under subsection (1), the Sponsor must report it until the adverse drug reaction is concluded (referring to the disappearance of the ADR or the inability to conduct a follow-up).

(3) When the Sponsor intends to report the ADR to the Minister of Food and Drug Safety pursuant to subsection (1), an ADR summary, including CIOMS-I form, must be attached to Form 77, the ADR report.

7.4.5 Handling AE

All AEs which occur during the study period must be recorded in detail in the Case Report Form, including symptoms and signs, start date/end date, duration, severity, treatment and results, and causality with the investigational product even if they are not related to the investigational product. In addition, if possible, the AE shall be observed until it is recovered to pre-administration or baseline level, or until the investigator can determine that the AE has been normalized, or until further observation is deemed unnecessary.

Handling of the AE is categorized as follows:

1) Drug administration maintained
2) Drug administration interrupted
3) Drug administration permanently discontinued

4) N/A: The subject has deceased, or administration is terminated when the AE occurs
5) Unknown

Treatments of the AE are categorized as follows:

1) Perform drug treatment of the AE
2) Perform non-drug treatment of the AE
3) Perform drug/non-drug treatment of the AE
4) No drug/non-drug treatment of the AE

Results of the AE are categorized as follows:

1) Recovered (resolved)
2) Recovering (being resolved)
3) Not recovered (not resolved)
4) Recovered (resolved) with sequelae
5) Death possibly related to the AE
6) Death not related to the AE
7) Unknown

7.4.6 Handling related to investigational product

During the clinical study, the principal investigator and sub-investigator(s) must make every effort to secure the safety of the subjects and take prompt and appropriate interventions to minimize AEs when SAEs occur. The principal investigator may discontinue the clinical study in consultation with the Sponsor. Even when not related to the investigational product, all AEs that occur during the clinical study period must be recorded in detail in the Case Report Form, with the symptoms and signs, start date/end date of occurrence, duration, severity, treatment and results, and causality with the investigational product. In addition, if possible, cases shall be observed until the AE is recovered to the pre-administration or baseline level, or until the investigator can determine that the AE has been normalized, or until further observation is deemed unnecessary.

When an AE occurs, the following interventions shall be taken:

1) Dose maintained
2) Dose increased
3) Dose reduced
4) Drug administration interrupted
5) Drug administration permanently discontinued

6) N/A: The subject has deceased, or administration is terminated when the AE occurs
7) Unknown

7.4.7 Pregnancy report

Female subjects of childbearing potential and male subjects who are sexually active with women of childbearing potential must practice appropriate contraception until the 7th visit (last visit, Day 270) after the last administration of the investigational product.
The appropriate contraception methods acknowledged in this clinical study are:

- Hormonal contraceptives
- Insertion of an Intrauterine device or system
- Double barrier method*(spermicide and condom with vaginal diaphragm, vaginal sponge, or cervical cap)
  * Both male (condom) and female (vaginal diaphragm, vaginal sponge or cervical cap) must use contraceptive devices together with spermicide.
- Sterilization surgery (vasectomy, bilateral tubal ligation, etc.)
- Complete abstinence: If preferred by the subject and matched with everyday lifestyle. [Periodic abstinence and coitus interruptus are not acknowledged as contraception.]

The Sponsor has the obligation to follow up on the results of pregnancy reported by female and male subjects during the clinical study. If it is not suspected that the investigational product interfered with the efficacy of appropriate contraception and contraceptives, the pregnancy itself is not considered an AE. In addition, voluntary induced abortion without complications, other than therapeutic abortion, is not considered an AE.

Within 24 hours of becoming aware of pregnancy, the Investigator shall prepare an initial pregnancy report and report it to the Sponsor. The Investigator shall track and document the process and results of all pregnancies even if the subject withdraws consent or terminates the clinical study. In addition, the investigator shall prepare a pregnancy result report and report it to the Sponsor within 24 hours of becoming aware of the results of any pregnancy (e.g., spontaneous labor, spontaneous abortion, etc.).

All SAEs (e.g., severe maternal complications, premature birth, therapeutic abortion, ectopic pregnancy, stillbirth, neonatal death, congenital anomaly, birth defects, etc.) that occurred from beginning of pregnancy to four weeks after childbirth shall be recorded in the SAE report and must be reported immediately to the PVA of DT&SanoMedics Co., Ltd., following the procedure in 7.4 Adverse Events.

8 Statistical Method

8.1 Analysis Set

Efficacy assessment shall use the intention-to-treat (ITT) populations as the analysis sets, and analysis shall be performed auxiliarily in the per-protocol (PP) sets. The definition of each analysis set for the assessment of safety, tolerability, and efficacy is as follows:

8.1.1 Subject Set to Be Included in Safety and Tolerability Assessment Analyses: Safety set

Among all subjects evaluated for their eligibility during the screening period, those subjects whose safety can be assessed after being administered the investigational product shall be included in the safety analysis. The subjects included in the safety analysis shall be analyzed based on the information of the actually administered investigational product. In addition, the demographic data (sex, age, etc.) and background factors (medical history, previous drug treatment history, etc.) shall be analyzed in the safety set.

8.1.2 Subject Set to Be Included in Efficacy Assessment Analysis: Intention-to-treat (ITT) set

Subjects who have been administered the investigational product and have undergone efficacy assessment at least once shall be included in the ITT analysis set, regardless of protocol violations, compliance with visit schedule, etc.

8.1.3 Subject Set to Be Included in Efficacy Assessment Analysis: Per-protocol (PP) set

PPS shall include those subjects in the ITT set who have completed the clinical study according to the protocol without major protocol violations. Subjects who fall under the following shall be defined as PPS. Compliance with No. ① eligibility shall be determined in a data review meeting prior to datalock.

① Subjects who have not violated the inclusion and exclusion criteria (eligible patients)

② Subjects who have completed all visits

8.1.4 Subgroup Analysis Population

When conducting the adverse events and efficacy assessments, subgroup analysis shall be performed by considering the following items:

- Sex (male, female)
- Age (≤ median, > median)
- Baseline BMI (≤ median, > median)
- Presence or absence of medical history
- Presence or absence of concomitant drugs
- Disease severity (CMTNS-v2) (mild, moderate)

If there are only a few subjects in each set who correspond to the above subgroup, analysis shall not be performed for the applicable subgroup. For example, if ≤ 30% of the total number of subjects is male, no separate analysis for sex shall be performed, and whether a subgroup analysis should be performed shall be determined in a data review meeting prior to datalock.

8.2 Handling of Missing Values

For missing values due to early termination of subjects, they shall not be imputed and shall be analyzed using the available data set.

8.3 Classification of Subjects

Screen Failure - This is a subject who has signed the informed consent form in the clinical study but failed to satisfy the inclusion/exclusion criteria in the screening process. No follow-up for safety or pharmacodynamic assessment shall be conducted, neither shall any other clinical study procedure be performed.

Evaluable Subject - These are all of the subjects who have been administered the investigational product by participating in the clinical study. They shall be classified according to the definition of "8.1 Analysis Set" and analyzed.

Lost to Follow-up - This is a subject who has been administered the investigational product, but has not completed the planned visits. These include subjects who have withdrawn consent as well as subjects who have refused further participation in the clinical study and failed to respond to all attempts at contacting the subjects. Analysis shall be performed according to "8.2. Handling of Missing Values."

8.4 Statistical Analysis Method

8.4.1 General Principles of Statistical Analysis

Since this is a phase 1/2a clinical study and the number of subjects is small, the data from the treatment period and the follow-up period shall be descriptively compared and reviewed. For continuous variables, descriptive statistics (the number of subjects, mean, standard deviation, median, maximum, minimum) shall be presented, while frequency and percent shall be presented for categorical variables.

If necessary or if comparisons are possible, statistical testing shall be performed. The p-value shall be presented with four decimal places when testing, and a two-tailed test shall be performed under a significance level of 0.05. Adverse events shall be compared by calculating the 95% confidence interval. In addition, in descriptive statistics, values below the decimal point shall be presented with two decimal places.

8.4.2 Basic Information on Subjects and Disease

The demographic information (e.g., age, sex, etc.) of subjects as well as underlying characteristics prior to treatment (e.g., medical history, concomitant medications, etc.) shall be summarized.

Medical history shall be encoded using the system organ classes (SOCs) and preferre d terms (PTs) according to the latest version of the medical dictionary for regulatory activities (MedDRA), and the frequencies and percentages shall be presented.

Previous and concomitant medications shall be classified into the anatomical main gr oups and therapeutic subgroups according to the latest version of the anatomical th erapeutic chemical system (ATC CODE), and the frequencies and percentages shall be presented. In addition, previous therapies and concomitant therapies shall be encode d using the system organ classes (SOCs) and preferred terms (PTs) according to the l atest version of the medical dictionary for regulatory activities (MedDRA), and the fre quencies and percentages shall be presented.

8.4.3 Primary Endpoints

(1) Adverse events

: Summarization and analysis of adverse events shall be performed on treatment-emergent adverse events (TEAEs).

The frequencies and percentages shall be presented for the occurrence of treatment-emergent adverse events (TEAEs), adverse drug reactions (ADRs), serious adverse events (SAEs) and adverse events of special interests(AESIs), etc.

The adverse events, adverse drug reactions, and serious adverse events shall be encoded using the system organ classes (SOCs) and preferred terms (PTs) according to the latest

version of the medical dictionary for regulatory activities (MedDRA). The number of subjects with onset, the incidence, the number of cases, etc., shall be presented for the encoded adverse events.

(2) Laboratory tests and vital signs

: For continuous variables, descriptive statistics (mean, standard deviation, median, minimum, maximum) shall be presented for each visit. Frequencies and percentages shall be presented for categorical variables. The frequencies and percentages for normal shifts, not clinically significant (NCS) abnormal shifts, and clinically significant (CS) abnormal shifts shall be presented for each visit. The subjects who have been assessed as clinically significant (CS) at each visit shall be presented in a list.

8.4.4 Secondary Endpoints

(1) Severity of disease

This shall be measured using Charcot-Marie-Tooth Neuropathy Score version 2 (CMTNS-v2) and the functional disability scale (FDS).

: Descriptive statistics (frequency, percentage) for the changes in CMTNS-v2 and FDS at the termination visit (V7) compared with the baseline (V2) shall be presented. A contingency table shall be prepared for the differences before and after administration, and analysis shall be performed using the McNemar's test.

In addition, descriptive statistics (mean, standard deviation, median, minimum, maximum) shall be presented for the changes in FDS at each visit (v4, v6, v7) compared with the baseline (v2), and analysis shall be performed using a paired t-test (wilcoxon signed rank test if the assumption of normal distribution is not satisfied) for the differences at each visit compared with the baseline.

(2) Lower limb function

Changes in lower limb function shall be assessed using the overall neuropathy limitation score (ONLS) and the 10-meter walk test (10MWT).

: Descriptive statistics (mean, standard deviation, median, minimum, maximum) shall be presented for the changes in lower limb function (ONLS leg scale, 10MWT)) at each visit (V4, V6, V7) compared with the baseline (V2), and analysis shall be performed using a paired t-test (Wilcoxon signed rank test if the assumption of normal distribution is not satisfied) for the differences at each visit compared with the baseline.

(3) Degree of Fatty infiltration of lower limb muscles

The muscles of lower limbs shall be imaged with MRI leg scan, and the degree of fatty infiltration of the leg muscles injected with the investigational product shall be measured and evaluated as fat content value (%) at one level for each muscle.

: Descriptive statistics (mean, standard deviation, median, minimum, maximum) shall be presented for the changes in the degree of fatty infiltration of lower limb muscles at the termination visit (V7) compared with the baseline (V2), and analysis shall be performed for the differences before and after administration by using a paired t-test (Wilcoxon signed rank test if the assumption of normal distribution is not satisfied).

(4) Nerve regeneration potential

Nerve conduction studies including compound motor nerve action potential (CMAP), compound sensory nerve action potential (SNAP), and nerve conduction velocity (NCV) shall be performed.

: Descriptive statistics (mean, standard deviation, median, minimum, maximum) shall be presented for the changes in nerve conduction studies (CMAP, SNAP, NCV) at the termination visit (V7) compared with the baseline (V2), and analysis shall be performed for the differences before and after administration by using a paired t-test (Wilcoxon signed rank test if the assumption of normal distribution is not satisfied).

(5) HGF antibody production by Engensis (VM202)

The presence or absence of HGF antibody (anti-HGF Ab) in blood shall be verified with the ELISA method. A preliminary assessment shall be performed on whether antibody production is correlated between the subject group determined to have increased muscle mass and improved function, and the subject group not determined as such.

However, if antibodies in blood were not produced in any of the subjects, the results shall not be presented.

: Descriptive statistics (frequency, percentage) shall be presented for the changes in HGF antibody production at the termination visit (V7) compared with the baseline (V2). A contingency table shall be prepared for the differences before and after administration, and analysis shall be performed using the McNemar’s test.

9. Document Management

9.1 Case Report Form

Relevant documents (RDs) refer to the subject's records that shall be stored at the institution. Most source documents are charts of the investigator, and all information recorded in case report forms (CRFs) shall be consistent with the corresponding relevant documents.

This clinical study shall use electronic CRFs (eCRFs), and the development, maintenance, and data management of the eCRFs shall be performed by the contract research organization designated by Helixmith Co., Ltd. The entry and revision of data shall be performed by a person authorized by the principal investigator, and the final review and signing shall be performed by the principal investigator. The principal investigator shall guarantee that the information recorded in the CRF is true by signing, and shall be responsible for the accuracy and reliability of the information recorded in the CRF in all cases. When the entered data are revised, the revision details shall be automatically saved and the deletion of previously entered data shall not be allowed. If necessary, a copy of the eCRF shall be submitted, and HELIXMITH CO., LTD. shall store the original eCRF for three years from the termination date of the clinical study.

9.2 Recording and Collecting

The electronic data capture (EDC) system shall comply with Part 11 of Title 21 of the Code of Federal Regulations (21 CFR Part 11) and the guidelines for handling and management of electronic data of clinical studies. A data management plan (DMP) that defines all procedures related to DM tasks and the roles of relevant staff shall be established to document all procedures and output.

The EDC system shall be accessible only when authorized, and all actions such as inputting, revising, storing, and deleting the electronic case report form (eCRF) through the EDC system shall be tracked and recorded. Data validation to resolve omissions of data, as well as invalid, illogical, and inconsistent data shall be performed through computer programming and manual checking.

The principal investigator's electronic signature shall guarantee that the data entered in the eCRF are accurate, complete, interpretable, and timely. After termination of the study, copies of the eCRF shall be saved in electronic storage media and delivered to each institution, and they shall be stored in the same manner as other basic documents. The final database shall be output in the SAS format and sent to the person in charge of statistics.

9.3 Access to, Protection of, and Storage of Records

All data related to this study shall be stored in a restricted area, and only the delegate of the institution, the sponsor (or delegate), and designated persons of supervising regulatory authorities shall be allowed to view them. For confidentiality of the subjects' information, only the subjects' initials and identification numbers shall be recorded in all reports and data related to the study to identify the subjects. The investigator shall continually verify that the subject identification numbers are consistent, and this information shall be handled in accordance with professional confidentiality standards.

If there is a request from the Institutional Review Board or the Ministry of Food and Drug Safety, the investigator shall allow viewing of the documents related to the clinical study, and shall actively cooperate with requests such as submission of copies and verification of details. In addition, if a visitation has been notified by the Institutional Review Board or regulatory authorities including the Ministry of Food and Drug Safety, the investigator shall immediately inform the sponsor (or delegate), and may delegate his/her authority to the sponsor.

The investigator shall provide the following documents to the sponsor (or delegate) prior to initiating the study and the copies shall be stored in the trial master file.

- CVs and medical licenses (within two years) of the principal investigator and all co-investigators
- Copies of all clinical study approval letters issued by the Institutional Review Board (Matters related to the changing progress while conducting this clinical study shall be regularly submitted to the Institutional Review Board or shall be submitted in accordance with the policies of the Institutional Review Board.)
- Subject informed consent forms approved by the Institutional Review Board
- The signature page of this clinical study protocol that has been dated and signed by the principal investigator

All records related to the clinical study shall be stored for three years from the termination date of the clinical study.

The sponsor shall inform the principal investigator and the director of the institution in writing regarding the necessity of data storage and the storage period. If it is determined that storage is no longer required, the sponsor shall inform this fact in writing to the principal investigator

and the director of the institution.

The investigator shall not destroy any document without a notification from the sponsor. If transferring from the current institution to a different one, the principal investigator shall delegate document management to a person who takes his/her place, and inform the sponsor of the name of the delegated person as well as information on the document storage location. If documents related to the study are damaged or lost due to mistakes or accidents, the investigator shall promptly inform this to the sponsor.

10 Quality Control and Assurance

The sponsor and the contract research organization shall conduct the clinical study and prepare documents consistently in accordance with standardized methods based on the standard operating procedures (SOPs). Since compliance with regulations is crucial in clinical studies, the regulations of relevant regulatory agencies and the Korean Good Clinical Practice (KGCP) shall be complied with.

An audit of the reliability of this clinical study may be performed by the sponsor at any time during the clinical study or after its completion. The sponsor shall notify this fact in advance to the investigator selected to be audited, and the notified investigator shall provide cooperation to facilitate the audit. An audit shall be performed to establish the reliability of data collected in the relevant clinical study. Information related to the clinical study including the informed consent forms, case report forms, source documents, medical records, and regulatory documents shall be reviewed, and whether the clinical study is being conducted in accordance with the clinical study protocol, the sponsor's SOPs, the KGCP, and relevant regulations shall be verified. After an audit, a brief meeting shall be held to inform the investigator of the issues found in the auditing process, and a report shall be made using a standardized report form.

11 Informed Consent

The investigator of the clinical study has the responsibility to describe to the subject all information on the clinical study (purpose of the clinical study, tasks to perform when participating in the clinical study, potential benefits and risks, etc.), as well as the responsibility to obtain the informed consent form completed voluntarily by the subject. If there is a need to amend the included informed consent form, it shall first be approved by the Institutional Review Board. Actions related to the clinical study must not be performed until the subject has carefully read the informed consent form and has dated and signed the form in his/her own handwriting. The signed original informed consent form shall be stored at the institution, and a copy of the informed consent form shall be issued to the subject. All details on this subject consent procedure shall be recorded in a chart. The informed consent form shall be written in a way that can be easily understood by the subjects.

12 Approval of Clinical Study Protocol

The clinical study may start after submitting the clinical study protocol and relevant documents to the Ministry of Food and Drug Safety and the Institutional Review Board, and obtaining approval.

Before starting the clinical study, the clinical study protocol, informed consent form, and investigator's brochure shall be submitted to and approved by the Institutional Review Board. In addition, the clinical study investigator shall prepare and submit documents related to the principal investigator's statement. When the principal investigator signs the documents related to his/her statement, a promise to keep the responsibility of conducting the clinical study in accordance with relevant regulations is made. When approval letters for the clinical study protocol, informed consent form, and signature page of the clinical study protocol are issued by the Institutional Review Board, the investigator shall submit these to the sponsor before the investigational product is delivered to the institution. The institution shall accurately record and store all details of the approval letters including the documents that were prepared and reported in relation to the Institutional Review Board. The sponsor shall receive information on the members (names, positions or titles, affiliations, IRB number) of the Institutional Review Board before the investigational product and related articles are delivered to the institution.

In accordance with the regulations of the Ministry of Food and Drug Safety or health authorities, the details related to subject recruitment advertisements must be approved by the Institutional Review Board before starting the clinical study. The investigator shall submit these first to the sponsor for confirmation before submitting them to the Institutional Review Board to obtain approval.
In accordance with the regulations of the Institutional Review Board, the investigator has the responsibility to report serious adverse events that occur in the subjects to the Institutional Review Board. Once a report is submitted, a copy shall be delivered to the sponsor and the contract research organization.

Clinical study progress reports shall be submitted following the interval established based on the policy of the Institutional Review Board. In addition, if the clinical study has been completed (including early termination), the principal investigator shall report this to the Institutional Review Board. A close-out report shall be submitted within the timeline established based on the policy of the Institutional Review Board after termination of the study. The close-out report shall be prepared by including clinical study protocol violations, number of recruited subjects, number of evaluated subjects, subjects who were suspended or dropped out from the study and their reasons, adverse event details, and the principal investigator's final comments on the outcomes.

13 Confidentiality of Subjects' Records

Confidentiality for the information of subjects participating in this clinical study shall strictly maintained by all individuals related to the clinical study in accordance with KGCP and the Personal Information Protection Act.

The subjects shall be informed that all clinical study data will be stored in a computer and kept strictly confidential. The signed informed consent forms shall be kept by the principal investigator. The principal investigator shall store relevant records by keeping a list of subject numbers and subject names. The informed consent forms and the list of subjects shall be stored at the institution for 3 years from the termination date of the clinical study.

The investigator shall maintain confidentiality for all information on the clinical study, and may not, for any reason, provide relevant information to a third party (individual not related to the clinical study) without a written consent of the sponsor. However, information may be disclosed to work-related individuals who have agreed to maintain confidentiality.

14 Monitoring of Clinical Study

The sponsor may delegate duties or roles related to this study to a contract research organization. The clinical monitor authorized by the sponsor has the responsibility to supervise the progress of the clinical study. In addition, the clinical monitor shall visit the institution before the enrollment of subjects and also make regular visits, and shall have accurate knowledge of the clinical study in progress through phone calls and correspondence.

While visiting the institution, the monitor shall prepare to collect case report forms by reviewing source documents to verify the accuracy and completeness of the information used in completing the case report forms. All source documents shall contain all of the information required to complete the case report forms. All data and source documents recorded during this clinical study are subject to audit by the Ministry of Food and Drug Safety or other regulatory agencies.

The clinical monitor shall make a close-out visit for the termination of the clinical study. The close-out visit shall be performed to complete preparation of all regulatory records and reports, to arrange and collect the investigational product and study-related articles, and to clearly define the investigator's responsibilities after the termination of the clinical study.

15 Measures for Protection of Subjects' Safety

This clinical study shall be conducted scientifically and ethically in accordance with the KGCP as well as the relevant laws and regulations. Furthermore, this clinical study shall be conducted in accordance with the Declaration of Helsinki to respect the dignity as well as the rights and interests of human beings and not to cause disadvantages to the subjects. The Institutional Review Board shall evaluate/approve this clinical study protocol in accordance with the KGCP, and shall regularly evaluate whether the clinical study is being conducted according to the clinical study protocol.

The investigator shall verify the eligibility for study participation by checking the health status of each subject prior to enrollment in the clinical study. In addition, the investigator shall try his/her best to gain sufficient knowledge of the investigational product and ensure the safety of the subjects.

If an adverse event due to the clinical study occurs, appropriate medical interventions shall be taken until the subject recovers. The sponsor shall provide indemnification for injuries due to the investigational product in accordance with the subject indemnification policy.

16 Amendment of Clinical Study Protocol

Neither the principal investigator nor Helixmith Co., Ltd. may amend the details of this protocol during the clinical study without the consent of the other party. After the start of the clinical study, amendments shall be made only in exceptional cases. If an amendment is to be made in the clinical study protocol, all parties concerned shall agree in written form by providing their signatures. Amendments that may impact the safety of the subjects or the validity of the study may be implemented only if they are approved by the Ministry of Food and Drug Safety or the Institutional Review Board (IRB).

If the protocol is to be amended to immediately remove risk factors shown in a medical emergency that has occurred in a subject, it is recommended to discuss the matter with Helixmith Co., Ltd. or an individual designated by Helixmith Co., Ltd. These events shall be reported to the Ministry of Food and Drug Safety and the Institutional Review Board as quickly as possible.

17 Clinical Study Report (CSR)

When the clinical study is completed or early terminated, the investigator shall accurately prepare a CSR and submit it to the Institutional Review Board and the sponsor within one year. The CSR shall be submitted after all monitoring issues have been resolved in the EDC system.

18 Presentation and Publication of Clinical Study Report

The data obtained from the results of this study are intellectual properties of the clinical study sponsor, and they may be presented only when all data have been analyzed and the study results are available. All results obtained from this study may not be presented or published by the principal investigator or other investigators without a prior approval of the sponsor. When the study is terminated, the sponsor or a delegate designated by the sponsor shall prepare a clinical study report.

19 References

[1] Saporta AS, Sottile SL, Miller LJ, Feely SM, Siskind CE, Shy ME. Charcot-Marie-Tooth disease subtypes and genetic testing strategies. *Ann Neurol* 2011; 69:22–33.

[2] VanPaassen BW, van der Kooi AJ, van Spaendonck-Zwarts KY, Verhamme C, de Visser FB, de Visser M. PMP22 related neuropathies: Charcot-Marie-Tooth disease type 1A and Hereditary Neuropathy with liability to Pressure Palsies. *Orphanet J Rare Diseases* 2014; 9:38

[3] Szigeti K, Lupsk JR. Charcot–Marie–Tooth disease. *European J Human Genetics* 2009; 17:703-710

[4] Nam SH, Choi BO. Clinical and genetic aspects of Charcot-Marie-Tooth disease subtypes. *Precision and Future Medicine* 2019; 3(2): 43-68.

[5] Lupski JR, de Oca-Luna RM, Slaugenhaupt S, Pentao L, Guzzetta V, Trask BJ, Saucedo-Cardenas O, Barker DF, Killian JM, Garcia CA, Chakravarti A, Patel PI. DNA duplication associated with Charcot-Marie-Tooth disease type 1A, *Cell* 1991; 66(2): 219-232

[6] Dyck PJ, Lambert EH. Lower motor and primary sensory neuron diseases with peroneal muscular atrophy. I. Neurologic, genetic, and electrophysiologic findings in hereditary polyneuropathies. *Arch Neurol.* 1968; 18(6):603–618.

[7] Juneja M, Burns J, Saporta MA, et al. Challenges in modelling the Charcot-Marie-Tooth neuropathies for therapy development. *J Neurol, Neurosurg & Psychiatry* 2019; 90:58-67.

[8] Kim HS, Yoon YC, Choi BO, Jin W, Cha JG. Muscle fat quantification using magnetic resonance imaging: case-control study of Charcot-Marie-Tooth disease patients and volunteers. *J Cachexia Sarcopenia Muscle*. 2019;10(3):574-585.

[9] Vallat J-M, Sindou P, Preux P-M, Tabaraud F, Milor A-M, Couratier P, Leguern E, Brice A. Ultrastructural PMP22 expression in inherited demyelinating neuropathies. *Ann Neurol* 1996; 3 9:813-817.

[10] Roa BB, Garcia CA, Suter U, et al. Charcot-Marie-Tooth Disease Type 1A - Association with a Spontaneous Point Mutation in the PMP22 Gene. *N Engl J Med* 1993; 329:96-101

[11] Sancho S, Young P, Suter U, Regulation of Schwann cell proliferation and apoptosis in PMP22-deficient

mice and mouse models of Charcot–Marie–Tooth disease type 1A, *Brain* 2001; 124(11): 2177–2187

[12] Kim, YH, Kim, YH, Shin, YK, Jo YR, Park DK, Song MY, Uoon BA, Nam SH, Kim JH, Choi BO, Shin HY, Kim SW, Kim Sh, Hong YB, Kim JK, Park HT. p75 and neural cell adhesion molecule 1 can identify pathologic Schwann cells in peripheral neuropathies. *Ann Clin Transl Neurol* 2019; 6: 1292–1301.

[13] Pablo Sánchez-Martín, Tetsuya Saito, Masaaki Komatsu. p62/SQSTM1: 'Jack of all trades' in health and cancer. *FEBS J.* 2019, 286(1):8-23

[14] Jenny Fortun, William A. Dunn Jr, Shale Joy, Jie Li, and Lucia Notterpek. Emerging Role for Autophagy in the Removal of Aggresomes in Schwann Cells. *J. Neurosci.* 2003. 23(33) 10672-10680.

[15] Pantera H, Shy ME, Svaren J. Regulating PMP22 expression as a dosage sensitive neuropathy gene. *Brain Research* 2020; 1726, Article 146491

[16] Sereda MW, Meyer zu Hörste G, Suter U, Uzma N, Nave KA. Therapeutic administration of progesterone antagonist in a model of Charcot-Marie-Tooth disease (CMT-1A). *Nat Med,* 2003; 9: 1533–1537

[17] Passage E, Norreel J, Noack-Fraissignes P, Sanguedolce V, Pizant J, Thirion X, Robaglia-Schulpp A, Pellissier JF, Fontés M. Ascorbic acid treatment corrects the phenotype of a mouse model of Charcot-Marie-Tooth disease. *Nat Med* 2004; 10(4): 396–401

[18] Pareyson D, Reilly MM, Schenone A, Fabrizi GM, Cavallaro T, Santoro L, Vita G, Quattrone A, Padua L, Gemignani F, Visioli F, Laurà M, Radice D, Calabrese D, Hughes RA, Solari A; CMT-TRIAAL; CMT-TRAUK groups. Ascorbic acid in Charcot-Marie-Tooth disease type 1A (CMT-TRIAAL and CMT-TRAUK): a double-blind randomized trial. *Lancet Neurol.* 2011; 10(4):320-328.

[19] Khajavi M, Shiga K, Wiszniewski W, He F, Shaw CA, Yan J, Wensel TG, Snipes GJ, Lupski JR. Oral curcumin mitigates the clinical and neuropathologic phenotype of the Trembler-J mouse: a potential therapy for inherited neuropathy. *Am J Hum Genet* 2007; 81(3):438–453.

[20] Patzkó A, Bai Y, Saporta MA, Katona I, Wu X, Vizzuso D, Feltri ML, Wang S, Dillon LM, Kamholz J, Kirschner D, Sarkar FH, Wrabetz L, Shy ME. Curcumin derivatives promote Schwann cell differentiation and improve neuropathy in R98C CMT1B mice. *Brain* 2012; 135(Pt12): 3551-3566.

[21] Sahenk Z, Nagaraja HN, McCracken BS, King WM, Freimer ML, Cedarbaum JM, Mendell JR. NT-3 promotes nerve regeneration and sensory improvement in CMT1A mouse models and in patients. *Neurol*

2005; 65: 681-689.

[22] Vita G, Vita GL, Stancanelli C, Gentile L, Russo M, Mazzeo A. Genetic neuromuscular disorders: living the era of a therapeutic revolution. Part 1: peripheral neuropathies. *Neurol Sci* 2019; 40(4): 661-669

[23] Chumakov I, Milet A, Cholet N, Primas1 G, Boucard1 A, Pereira1 Y, Graudens1 E, Mandel1 J, Laffaire1 J, Foucquier1 J, Glibert1 F, Bertrand1 V, Nave K-A, Sereda MW, Vial1 E, Guedj1 M, Hajj1 R, Nabirotchkin1 S, Cohen D Polytherapy with a combination of three repurposed drugs (PXT3003) down-regulates Pmp22 over-expression and improves myelination, axonal and functional parameters in models of CMT1A neuropathy. *Orphanet J Rare Dis* 2014; 9, 201

[24] McCorquodale D, Pucillo EM, Johnson NE. Management of Charcot-Marie-Tooth disease: improving long-term care with a multidisciplinary approach. *J Multidiscip Healthc.* 2016; 9:7–19.

[25] Crosbie J, Burns J, Ouvrier RA. Pressure characteristics in painful pes cavus feet resulting from Charcot–Marie–Tooth disease, *Gait & Posture.* 2008; 28(4): 545-551

[26] Ramdharry GM, Day BL, Reilly MM, Marsden JF. Foot drop splints improve proximal as well as distal leg control during gait in Charcot-Marie-Tooth Disease. *Muscle Nerve* 2012; 46: 512-519.

[27] Weimer LH, Podwall D. Medication-induced exacerbation of neuropathy in Charcot Marie Tooth Disease. *J of Neurological Sciences* 2006; 242(1–2): 47-54

[28] Sun AP, Tang L, Liao Q, Zhang H, Zhang YS, Zhang J. Coexistent Charcot-Marie-Tooth type 1A and type 2 diabetes mellitus neuropathies in a Chinese family. *Neural Regen Res* 2015; 10(10): 1696–1699.

[29] Sman AD, Hackett D, Fiatarone Singh M, Fornusek C, Menezes MP, Burns J. Systematic review of exercise for Charcot-Marie-Tooth disease. *J Peripher Nerv Syst* 2015; 20: 347-362.

[30] Bussolino F, Di Renzo MF, Ziche M, Bocchietto E, Olivero M, Naldini L, Gaudino G, Tamagnone L, Coffer A, Comoglio PM. Hepatocyte growth factor is a potent angiogenic factor which stimulates endothelial cell motility and growth. *J Cell Biol* 1992 ;119(3):629-41

[31] Matsumoto K, Nakamura T. Emerging multipotent aspects of hepatocyte growth factor. *J Biochem* 1996; 119:591-600.

[32] Jayasankar V, Woo YJ, Pirolli TJ, et al. Induction of angiogenesis and inhibition of apoptosis by hepatocyte growth factor effectively treats postischemic heart failure. *J Card Surg* 2005; 20:93-101

[33] Nakagami H, Kaneda Y, Ogihara T, Morishita R. Hepatocyte growth factor as potential cardiovascular therapy. *Expert Rev Cardiovasc Ther* 2005; 3:513-9.

[34] Nakamura T, Sakai K, Nakamura T, Matsumoto K. Hepatocyte growth factor twenty years on: Much more than a growth factor. *J Gastroenterol Hepatol* 2011; 26 Suppl 1:188-202.

[35] Nakamura T, Mizuno S. The discovery of hepatocyte growth factor (HGF) and its significance for cell biology, life sciences and clinical medicine. *Proc Jpn Acad Ser B Phys Biol Sci* 2010; 86(6):588-610

[36] Hashimoto N, Yamanaka H, Fukuoka T, et al. Expression of HGF and c-Met in the peripheral nervous system of adult rats following sciatic nerve injury. *Neuroreport* 2001; 12:1403-7.

[37] Giacobini P, Messina A, Wray S, et al. Hepatocyte growth factor acts as a motogen and guidance signal for gonadotropin hormone-releasing hormone-1 neuronal migration. *J Neurosci* 2007; 27:431-45.

[38] Kato N, Nemoto K, Nakanishi K, et al. Nonviral gene transfer of human hepatocyte growth factor improves streptozotocin-induced diabetic neuropathy in rats. *Diabetes* 2005; 54:846-54

[39] Koike H, Morishita R, Iguchi S, Aoki M, Matsumoto K, Nakamura T, Yokoyama C, Tanabe T, Oghihara T, Kaneda Y. Enhanced angiogenesis and improvement of neuropathy by cotransfection of human hepatocyte growth factor and prostacyclin synthase gene. *FASEB J* 2003; 17:779-81.

[40] Tsuchihara T, Ogata S, Nemoto K, Okabayashi T, Nakanishi K, Kato N, Morishita R, Kaneda Y, Uenoyama M, Suzuki S, Amako M, Kawai T, Arino H. Nonviral Retrograde Gene Transfer of Human Hepatocyte Growth Factor Improves Neuropathic Pain-related Phenomena in Rats. *Mol Ther* 2009 Jan;17(1): 42-50.

[41] Tönges L, Ostendorf T, Lamballe F, Genestine M, Dono R, Koch J.-C, Bähr M, Maina F, Lingor P. Hepatocyte growth factor protects retinal ganglion cells by increasing neuronal survival and axonal regeneration in vitro and in vivo. *Journal of Neurochemistry* 2011, 117: 892-903

[42] Kitamura K, Iwanami A, Nakamura M, Yamane J, Watanabe K, Suzuki Y, Miyazawa D, Shibata S, Funakoshi H, Miyatake S, Coffin RS, Nakamura T, Toyama Y, Okano H. Hepatocyte growth factor promotes endogenous repair and functional recovery after spinal cord injury. *J Neurosci. Res* 2007; 85: 2332-2342

[43] Sheehan SM, Tatsumi R, Temm-Grove CJ, Allen RE. HGF is an autocrine growth factor for skeletal muscle satellite cells in vitro. *Muscle Nerve* 2000; 23: 239-245.

[44] Gal-Levi R, Leshem Y, Aoki S, Nakamura T, Halevy O. Hepatocyte growth factor plays a dual role in

regulating skeletal muscle satellite cell proliferation and differentiation. *Biochimica et Biophysica Acta (BBA) - Molecular Cell Research* 1998; 1402 (1): 39-51.

[45] Jessen KR, Mirsky R. Schwann Cell Precursors; Multipotent Glial Cells in Embryonic Nerves. *Frontiers in Molecular Neuroscience* 2019; 12: 69

[46] Nho B, Lee J, Lee J, Ko KR, Lee SJ, Kim S. Effective control of neuropathic pain by transient expression of hepatocyte growth factor in a mouse chronic constriction injury model. *FASEB J* 2018 ;32(9):5119-5131.

[47] Sun W, Funakoshi H, Nakamura T. Overexpression of HGF retards disease progression and prolongs life span in a transgenic mouse model of ALS. *The Journal of neuroscience: the official journal of the Society for Neuroscience* 2002; 22:6537-48.

[48] Kadoyama K, Funakoshi H, Ohya W, Nakamura T. Hepatocyte growth factor (HGF) attenuates gliosis and motoneuronal degeneration in the brainstem motor nuclei of a transgenic mouse model of ALS. *Neuroscience research* 2007; 59: 446-56.

[49] Tyndall SJ, Walikonis RS. Signaling by hepatocyte growth factor in neurons is induced by pharmacological stimulation of synaptic activity. *Synapse* 2007; 61:199-204.

[50] Akita H, Takagi N, Ishihara N, Takagi K, Murotomi K, Funakoshi H, Matsumoto K, Nakamura T, i Takeo S. Hepatocyte growth factor improves synaptic localization of the NMDA receptor and intracellular signaling after excitotoxic injury in cultured hippocampal neurons. *Experimental Neurology* 2008; 210(1): 83-94.

[51] Shang J, Deguchi K, Ohta Y, Liu N, Zhang X, Tian F, Yamashita T, Ikeda Y, Matsuura T, Funakoshi H, Nakamura T, Abe K. Strong neurogenesis, angiogenesis, synaptogenesis, and antifibrosis of hepatocyte growth factor in rat's brain after transient middle cerebral artery occlusion. *J Neuroscience Res* 2011; 89:86-95.

[52] Ishihara N, Takagi N, Niimura M, Takagi K, Nakano M, Tanonaka K, Funakoshi H, Matsumoto K, Nakamura T, Takeo S. Inhibition of apoptosis-inducing factor translocation is involved in protective effects of hepatocyte growth factor against excitotoxic cell death in cultured hippocampal neurons. *J Neurochemistry* 2006; 95:1277-86.

[53] Ishigaki A, Aoki M, Nagai M, Warita H, Kato S, Kato M, Nakamura T, Funakoshi H, Itoyama Y. Intrathecal Delivery of Hepatocyte Growth Factor From Amyotrophic Lateral Sclerosis Onset Suppresses Disease

Progression in Rat Amyotrophic Lateral Sclerosis Model. *J Neuropatho & Experimental Neurol* 2007; 66 (11): 1037–1044.

[54] Genestine M, Caricati E, Fico A, Richelme S, Hassani H, Sunyach C, Lamballe F, Panzica GC, Pettmann B, Helmbacher F, Raoul C, Maina F, Dono R. Enhanced neuronal Met signaling levels in ALS mice delay disease onset. *Cell death & disease* 2011; 2: e130.

[55] Bottaro DP, Rubin JS, Faletto DL, Chan AM, Kmiecik TE, Vande Woude GF, Aaronson SA. Identification of the hepatocyte growth factor receptor as the c-met proto-oncogene product. *Science* 1991; 251(4995):802-4.

[56] Kawaida K, Matsumoto K, Shimazu H, Nakamura T. Hepatocyte growth factor prevents acute renal failure and accelerates renal regeneration in mice. *Proc Natl Acad Sci U S A* 1994; 91:4357-61.

[57] Liu ML, Mars WM, Zarnegar R, Michalopoulos GK. Uptake and distribution of hepatocyte growth factor in normal and regenerating adult rat liver. *Am J Pathol* 1994; 144:129-40.

[58] Gille J, Khalik M, Konig V, Kaufmann R. Hepatocyte growth factor/scatter factor (HGF/SF) induces vascular permeability factor (VPF/VEGF) expression by cultured keratinocytes. *J Invest Dermatol* 1998; 111:1160-5.

[59] Morishita R, Aoki M, Yo Y, Ogihara T. Hepatocyte growth factor as cardiovascular hormone: role of HGF in the pathogenesis of cardiovascular disease. *Endocr J* 2002; 49:273-84.

[60] Henry TD, Hirsch AT, Goldman J, Wang YL, Lips DL, McMillan WD, Duval S, Biggs TA, Keo HH. Safety of a non-viral plasmid-encoding dual isoforms of hepatocyte growth factor in critical limb ischemia patients: a phase I study. *Gene therapy* 2011; 18(8):788-94

[61] Kibbe MR, Hirsch AT, Mendelsohn FO, Davies MG, Pham H, Saucedo J, Marston W, Pyun WB, Min SK, Peterson BG, Comerota A, Choi D, Ballard J, Bartow RA, Losordo DW, Sherman W, Driver V, Perin EC. Safety and efficacy of plasmid DNA expressing two isoforms of hepatocyte growth factor in patients with critical limb ischemia. *Gene therapy* 2016; 23(3):306-12.

[62] Ajroud-Driss S, Christiansen M, Allen JA, Kessler JA. Phase 1/2 open-label dose-escalation study of plasmid DNA expressing two isoforms of hepatocyte growth factor in patients with painful diabetic peripheral neuropathy. *Molecular therapy* 2013; 21(6):1279-86.

[63] Kessler JA, Smith AG, Cha BS, Choi SH, Wymer J, Shaibani A, Ajroud-Driss S, Vinik A; VM202 DPN-II

Study Group. Double-blind, placebo-controlled study of HGF gene therapy in diabetic neuropathy. *Ann Clin Transl Neurol* 2015; 2(5):465-78

[64] Sufit RL, Ajroud-Driss S, Casey P, Kessler JA. Open label study to assess the safety of VM202 in subjects with amyotrophic lateral sclerosis. *Amyotrophic Lateral Sclerosis and Frontotemporal Degeneration* 2017; 18(3-4): 269-278

[65] Kim JS, Hwang HY, Cho KR, Park EA, Lee W, Paeng JC, Lee DS, Kim HK, Sohn DW, Kim KB. Intramyocardial transfer of hepatocyte growth factor as an adjunct to CABG: phase I clinical study. *Gene therapy* 2013; 20(7):717-22.

[66] Murphy SM, Herrmann DN, McDermott MP, Scherer SS, Shy ME, Reilly MM, Pareyson D. Reliability of the CMT neuropathy score (second version) in Charcot-Marie-Tooth disease. *J Peripher Nerv Syst* 2011; 16(3): 191-198

[67] Kim HS, Yoon YC, Choi BO, Jin W, Cha JG. Muscle fat quantification using magnetic resonance imaging: case–control study of Charcot–Marie–Tooth disease patients and volunteers. *J Cachexia, Sarcopenia and Muscle* 2019; 10: 574– 585.

[68] Birouk N, Gouider R, Le Guern E, Gugenheim M, Tardieu S, Maisonobe T, Le Forestier N, Agid Y, Brice A, Bouche P. Charcot-Marie-Tooth disease type 1A with 17p11.2 duplication. Clinical and electrophysiological phenotype study and factors influencing disease severity in 119 cases. *Brain* 1997; 120:813- 823.

[69] Graham RC, Hughes RAC. A modified peripheral neuropathy scale: The Overall Neuropathy Limitations Scale. *J Neurol Neurosurg & Psychiatry* 2006; 77: 973-976.

[70] Graham JE, Ostir GV, Kuo YF, Fisher SR, Ottenbacher KJ. Relationship Between Test Methodology and Mean Velocity in Timed Walk Tests: A Review. *Arch Phys Med Rehabil* 2008; 89(5): 865-872

[71] Pareyson D, Marchesi C. Diagnosis, natural history, and management of Charcot–Marie–Tooth disease. *Lancet Neurol* 2009; 8: 654–67

[72] Banchs I, Casasnovas C, Albertí A, De Jorge L, Povedano M, Montero J, Martínez-Matos JA, Volpini V. Diagnosis of Charcot-Marie-Tooth Disease. *J Biomed and Biotech* 2009; 985415

[73] El-Abassi R, England JD, Carter GT. Charcot-Marie-Tooth Disease: An Overview of Genotypes, Phenotypes, and Clinical Management Strategies. *Phys Med & Rehab* 2014; 6:342-355

[74] Bassam BA. Charcot–Marie–Tooth Disease Variants—Classification, Clinical, and Genetic Features and Rational Diagnostic Evaluation. *J Clin Neuromusc Dis* 2014; 15:117–128

[75] Casasnovas C, Cano LM, Alberti A, Céspedes M, Rigo G. Charcot-Marie-Tooth Disease. *Foot & Ankle Specialist* 2008; *1*(6): 350–354.

[76] Vallata JM, Mathis S, Funalot B. The various Charcot–Marie–Tooth diseases. *Curr Opin Neurol* 2013; 26(5):473-80

[77] Young P, De Jonghe P, Stögbauer F, Butterfass-Bahloul T. Treatment for Charcot-Marie-Tooth disease (Review). *Cochrane Database Syst Rev* 2008;2008(1):CD006052.

[78] Greenblatt DJ, Allen MD. Intramuscular injection-site complications. *JAMA* 1978; 240:542-4.

[79] Müller-Vahl H. Adverse reactions after intramuscular injections. *Lancet* 1983:1050.

[80] Mishra P, Stringer MD. Sciatic nerve injury from intramuscular injection: a persistent and global problem. *Int J Clin Pract* 2010; 64(11):1573-1579.

[81] Tak SR, Dar GN, Halwai MA, Mir MR. Post-injection nerve injuries in Kashmir Menace overlooked. *J Research in Med Sci* 2008; 13(5) 244-247.

[82] Rodger MA, King L. Drawing up and administering intramuscular injections: a review of the literature. *J Adv Nurs* 2000; 31(3): 574-82

[83] Cocoman A, Murray J. Intralmuscular injection: a review of best practice for mental health nurses. *J Psychiatr Ment Health Nurs* 2008; 15(5): 424–434

[84] Blum L, Korner-Bitensky N. Usefulness of the Berg Balance Scale in Stroke Rehabilitation: A Systematic Review. *Phys Ther* 2008; 88(5): 559–566

[85] Pareyson D, Scaioli V, Laurà M. Clinical and electrophysiological aspects of Charcot-Marie-Tooth disease. *Neuromolecular Med* 2006; 8(1-2):3–22
